# Genome-by-Trauma Exposure Interactions in Adults With Depression in the UK Biobank

**DOI:** 10.1001/jamapsychiatry.2022.2983

**Published:** 2022-09-28

**Authors:** Melisa Chuong, Mark J. Adams, Alex S. F. Kwong, Chris S. Haley, Carmen Amador, Andrew M. McIntosh

**Affiliations:** 1 Institute of Genetics & Cancer, University of Edinburgh, Edinburgh, United Kingdom; 2 Department of Psychiatry, University of Edinburgh, Edinburgh, United Kingdom

## Abstract

**Importance:**

Self-reported trauma exposure has consistently been found to be a risk factor for major depressive disorder (MDD), and several studies have reported interactions with genetic liability. To date, most studies have examined gene-environment interactions with trauma exposure using genome-wide variants (single-nucleotide variations [SNVs]) or polygenic scores, both typically capturing less than 3% of phenotypic risk variance.

**Objective:**

To reexamine genome-by-trauma interaction associations using genetic measures using all available genotyped data and thus, maximizing accounted variance.

**Design, Setting, and Participants:**

The UK Biobank study was conducted from April 2007 to May 1, 2016 (follow-up mental health questionnaire). The current study used available cross-sectional genomic and trauma exposure data from UK Biobank. Participants who completed the mental health questionnaire and had available genetic, trauma experience, depressive symptoms, and/or neuroticism information were included. Data were analyzed from April 1 to August 30, 2021.

**Exposures:**

Trauma and genome-by-trauma exposure interactions.

**Main Outcomes and Measures:**

Measures of self-reported depression, neuroticism, and trauma exposure with whole-genome SNV data are available from the UK Biobank study. Here, a mixed-model statistical approach using genetic, trauma exposure, and genome-by-trauma exposure interaction similarity matrices was used to explore sources of variation in depression and neuroticism.

**Results:**

Analyses were conducted on 148 129 participants (mean [SD] age, 56 [7] years) of which 76 995 were female (52.0%). The study approach estimated the heritability (SE) of MDD to be approximately 0.160 (0.016). Subtypes of self-reported trauma exposure (catastrophic, adult, childhood, and full trauma) accounted for a significant proportion of the variance of MDD, with heritability (SE) ranging from 0.056 (0.013) to 0.176 (0.025). The proportion of MDD risk variance accounted for by significant genome-by-trauma interaction revealed estimates (SD) ranging from 0.074 (0.006) to 0.201 (0.009). Results from sex-specific analyses found genome-by-trauma interaction variance estimates approximately 5-fold greater for MDD in male participants (0.441 [0.018]) than in female participants (0.086 [0.009]).

**Conclusions and Relevance:**

This cross-sectional study used an approach combining all genome-wide SNV data when exploring genome-by-trauma interactions in individuals with MDD; findings suggest that such interactions were associated with depression manifestation. Genome-by-trauma interaction accounts for greater trait variance in male individuals, which points to potential differences in depression etiology between the sexes. The methodology used in this study can be extrapolated to other environmental factors to identify modifiable risk environments and at-risk groups to target with interventions.

## Key Points

**Question:**

Are genome-by-trauma exposure interactions associated with major depressive disorder?

**Findings:**

In this cross-sectional study, a mixed linear model design was implemented using 148 129 UK Biobank participants with available genomic and trauma exposure data to explore depression and neuroticism variance attributable to genomic, trauma exposure, and genome-by-trauma exposure interaction associations. Findings suggest genome-by-trauma exposure interactions can explain up to 20% of variation in major depressive disorder and more often in male vs female participants.

**Meaning:**

Exploring mechanisms underlying genome-by-trauma exposure interactions may be useful in identifying at-risk individuals and intervention targets; insight into these mechanisms may provide explanations for depression prevalence differences across the different sexes.

## Teaser

This cross-sectional study investigates if genome-by-trauma exposure interactions are associated with major depressive disorder.

## Introduction

Depression is a highly prevalent psychiatric disorder with a lifetime risk of approximately 16%^1,2^ and is a leading cause of disability worldwide.^3^ Twin studies provide moderate heritability estimates of 30% to 40%^4^ suggesting that both genetic and environmental factors are influential. Furthermore, meta-analyses of genome-wide association studies (GWAS) have uncovered many single-nucleotide variations (SNVs) associated with depression; however, SNV-based heritability estimates of 5% to 10%^5,6^ are much lower than estimates obtained from twin studies, the difference being termed *missing heritability*. Explanations for missing heritability include heterogeneity in sample processing, phenotyping methodology, and phenotype heterogeneity across medical systems, countries, and cultures within GWAS used in meta-analyses, as well as inflation of twin-based heritability estimates attributable to shared environmental effects and gene-environment interplay.^7,8,9,10^

Self-reported trauma exposure has been found to play a role in depression, with case-control studies suggesting individuals diagnosed with major depressive disorder (MDD) report higher levels of trauma exposure.^11,12,13^ In turn, trauma exposure in childhood has been consistently associated with adverse outcomes including increased risk of MDD in adolescence and adulthood.^14,15^ These findings suggest the role of trauma exposure in depression’s etiology needs further exploration. Moreover, research has shown self-reported trauma exposure and MDD are genetically correlated, suggesting shared genetic risk factors for both^16^ or potentially more complex interplay effects on depression manifestation.

One key form of interplay that has been explored is the interaction effect of genetics and trauma exposure on depression manifestation. Gene-by-environment interactions refer to the differential associations between an environmental exposure on traits in individuals with differing genotypes. This can be conceptualized as an individual’s genetic sensitivity to certain environments, which may result in an exacerbated risk of a disorder.^17,18^ Minimal evidence of interactions has been yielded from studies exploring SNV-by-trauma associations.^16,19,20,21,22^

Moreover, research using polygenic scores (PGSs)—genetic measures that can be calculated for each individual by identifying, weighting, and summing genotyped risk variants found to be associated with depression^23,24^—have yielded inconsistent findings. Some studies have highlighted sex differences^25^ and found significant interaction associations with MDD outcomes,^16,18,25,26,27^ whereas some replication attempts reported null findings.^28,29,30^ An explanation for inconsistent findings may lie in the predictive accuracy and validity of PGSs.^10^ PGSs build on the information provided by GWAS, which still have limited statistical power for detecting trait associated genetic variants and their effect sizes.^24,31,32^ These power limitations of GWAS are greater for traits that have a substantial environmental component, such as depression, as opposed to traits with higher genetic etiology.^33^ This is reflected in the fact that current PGSs capture less than 3%^5,6^ of the phenotypic variance proposed for depression.

One way to circumvent the issues associated with PGSs is to make use of genetic measures that capture greater variance. Genomic similarity matrices have been able to do this by using all genotyped SNVs.^34^ These matrices capture genetic similarity between individuals within a sample based on the number of genotyped SNVs they have in common, including matrices representing genetic, environmental, and interaction association similarity within a sample. In mixed linear models, these matrices can provide estimates of the genetic, environmental, and genome-by-environment interaction components of trait variance.^35^

Here, we estimated the contribution of trauma exposure and its interaction with genetic variation to depression and neuroticism. We chose to explore neuroticism as this trait has been shown to have a greater genetic component^36,37^ and has a strong phenotypic link with MDD, suggesting the exploration of neuroticism to be useful in understanding the genetic etiology of both of these traits.^38,39^ As the existing literature has highlighted the role of trauma exposure in sex differences observed in MDD, we also explored these associations in male and female participants separately.^40^ Here we show that using the entirety of genotyped genetic variants can improve statistical power in the exploration of genome-by-trauma interactions. More importantly, our findings suggest that genome-by-trauma interactions may play a much larger role in depression manifestation than previously thought.

## Methods

Data for the current study were obtained from the UK Biobank (UKB), a national study that explored environmental and genetic determinants of health. Individuals in UKB were recruited from 22 centers across the UK. The UKB study received ethical approval from the NHS National Research Ethics Service and has approval from the North West Multi-Centre Research Ethics Committee. This study has been approved by the UKB Access Committee. Participants in the UKB study provided written informed consent. In the current study, we explored variance components of Composite International Diagnostic Inventory (CIDI) depression, broad depression, and neuroticism. Information on participants, phenotypes, and genotyped data is available in the eMethods and eTables 1-14 in the Supplement. Data analyzed were limited to individuals with self-reported White British ethnicity to avoid confounding of genetic effects, which may arise owing to population substructure observed in admixed populations. This study followed the Strengthening the Reporting of Observational Studies in Epidemiology (STROBE) reporting guidelines.

### Covariance Matrices

Covariance matrices were used to explore genetic, environmental, and genome-by-environment components of variation. As the number of participants with available depression/neuroticism and trauma information is large, analyzing the pairwise covariance matrices jointly for the whole cohort was computationally intractable. To work around these computational issues, participants were split into 5 different clusters based on the geographic location of their recruitment centers (north, midnorth, midsouth, southwest, and southeast UK region) and all subsequent analyses were replicated across the 5 clusters. Information on how clusters were formed, and their demographic characteristics is available in eFigure 2 and eTables 23 to 31 in the Supplement.

#### Genomic Similarity Matrices 

Genomic similarity matrices (G) represent the expected genetic similarity between individuals. We computed 1 G for all individuals in each of the geographical clusters using genetic variants that passed quality control (eMethods and eAppendix 2 in the Supplement) using GCTA, version 1.91.4beta.^34^ Supplementary Gs were computed using only unrelated individuals (individuals with genomic similarity values <0.05).

#### Trauma Environmental Similarity Matrices 

Trauma environmental similarity matrices (E) represent similarity between individuals based on their trauma eigenvector principal components (PCs) (eMethods and eTables 15-22 in the Supplement). Separate Es were computed by calculating participant similarity based on their full trauma, childhood, adult, and catastrophic trauma eigenvectors. Supplementary Es were computed using PC1 of full trauma, as this accounted for the greatest variance in our outcome phenotypes.

Evidence suggests that gene-environment correlations and interactions often co-occur and disregarding 1 effect can result in biased findings.^41^ To control for genetic covariance between trauma exposure and our phenotypes of interest (depression/neuroticism), the impact of G was removed from our trauma variables. Trauma eigenvectors were precorrected for the full G, by regressing each trauma PC on G. The residuals obtained from these analyses were used to compute an additional supplementary E. If the presence of gene-environment correlations bias our results, we expect estimates of variance attributable to G × E to be different to estimates observed from models using E matrices without controlling for covariance between G and E.

We used OSCA, version 0.45 default algorithm 1^42^ to compute these matrices. More information on available algorithms is available in eAppendix 1 and eTable 32 in the Supplement.

#### Gene-Environment Interaction Similarity Matrices 

Gene-environment interaction similarity matrices (G × E) represent shared genome-by-trauma interactions. These were computed by multiplying G and Es using a cell-by-cell (Hadamard) product.^35,43,44^

### Statistical Analysis

#### Genetic Correlations

Using the first eigenvector (PC1) for the subtypes of trauma, the SNV heritability of each trauma variable was explored. Heritability estimates of the trauma variables were obtained by fitting the trauma variables as the dependent variable and Gs as random effects within a mixed linear model framework (estimates of trauma PC1 variance attributable to G) noted in model 1. The genetic correlations between trauma PC1 and depression variables were explored using the moment-based method, Haseman-Elston regression analyses, where SEs were calculated using a leave-one-individual-out jackknife technique. Age, sex, genotyping array, and the first 15 principal components of the G were included as covariates.

#### Variance Components Analyses

Variance components of depression/neuroticism were explored within mixed linear model frameworks. Four models were explored with varying levels of complexity:

1. *y* = *Xβ *+ *g* + *ε*

2. *y* = *Xβ* + *e* + *ε*

3. *y* = *Xβ* + *g* + *e* + *ε*

4. *y* = *Xβ* + *g* + *e* + *g* × *e* + *ε*

Where *y* is a *n × *1 vector of observed depression/neuroticism phenotypes; *β* is a vector of fixed effects (which include age, sex, genotyping array, and the first 15 principal components of the full sample G), and *X* is its design matrix; *g* is an *n × *1 vector of SNV effects (representing additive genetic effects) with *g* approximately Norm(*0*,*G*[*σ* for *G*]^2^); *e *is an *n × *1 vector representing common environmental effects of childhood, adult, catastrophic, or all trauma with *e* approximately Norm(*0*,*E*[*σ* for *E*]^2^); *g* × *e* is an *n × *1 vector representing interactions between genetic and trauma effects with *g* × *e* approximately Norm(*0*,*G* × *E*[σ for *G* × *E*]^2^); and *ε* is an *n × *1 vector of residual effects.

Estimates of variance attributable to the G, E, and G × E components are obtained from analyses using CIDI and broad depression as dependent variables and are converted to the liability scale within GCTA.^34^

We used the prevalence rates observed within the whole sample, which were in line with prevalence rates obtained from external data.^45^ Prevalence rates used were 0.28 and 0.35 for joint sex analyses, 0.35 and 0.43 for female participant analyses, and 0.19 and 0.27 for male participant analyses for CIDI and broad depression, respectively. We conducted the analyses using different prevalence estimates (0.16, 0.20, 0.28) as sensitivity analyses. Although estimates are slightly different, the overall pattern of significance remain unchanged (eTable 46 in the Supplement).

Analyses were repeated using only unrelated individuals. All analyses were replicated across the 5 geographic cluster samples using GCTA, version 1.91.4beta,^34^ and results (estimates of variance components) were meta-analyzed using R package metafor (R Project for Statistical Computing).

Note that interactions between main effects (G and E) with covariates were not included in these mixed linear models. This would have required additional matrices capturing covariate similarity, and thus, multiple additional matrices to be included in the models, which would have made analyses computationally intractable. Analyses were conducted for male and female participants separately, which is aimed to work around these computational issues (eTables 42-45 in the Supplement). Data were analyzed from April 1 to August 30, 2021.

## Results

Analyses were conducted among the 148 129 participants (mean [SD] age, 56 [7] years; 76 995 female [52.0%]; 71 134 male [48.0%]). Initial analyses explored SNV heritability of the first PC of full trauma and subcategories of trauma. Trauma variables meta-analyzed SNV heritability (SE) estimates including the following: full trauma, 0.17 (0.008); childhood trauma, 0.15 (0.008); adult trauma, 0.063 (0.008); and catastrophic trauma, 0.11 (0.008) (eTable 33 in the Supplement). Similar results were obtained when using only unrelated individuals.

All genetic correlations between PC1 of trauma variables and depression/neuroticism phenotypes are shown in Table. Genetic correlations between the PC1 of trauma variables and broad depression/neuroticism phenotypes were modest; in contrast, we observed stronger genetic correlations between trauma variables and CIDI depression. Results from each cluster can be found in eTable 34 in the Supplement.

**Table.** Genetic Correlations Between Trauma and Depression/Neuroticism Phenotypes

Phenotype	Trauma, estimate (SE)			
	Full	Childhood	Adult	Catastrophic
Depression				
CIDI	0.632 (0.085)	0.605 (0.091)	0.647 (0.134)	0.536 (0.104)
Broad	0.39 (0.082)	0.337 (0.09)	0.358 (0.132)	0.31 (0.098)
Neuroticism	0.333 (0.064)	0.332 (0.071)	0.274 (0.105)	0.204 (0.081)

Abbreviation: CIDI, Composite International Diagnostic Inventory.

**Figure 1.**
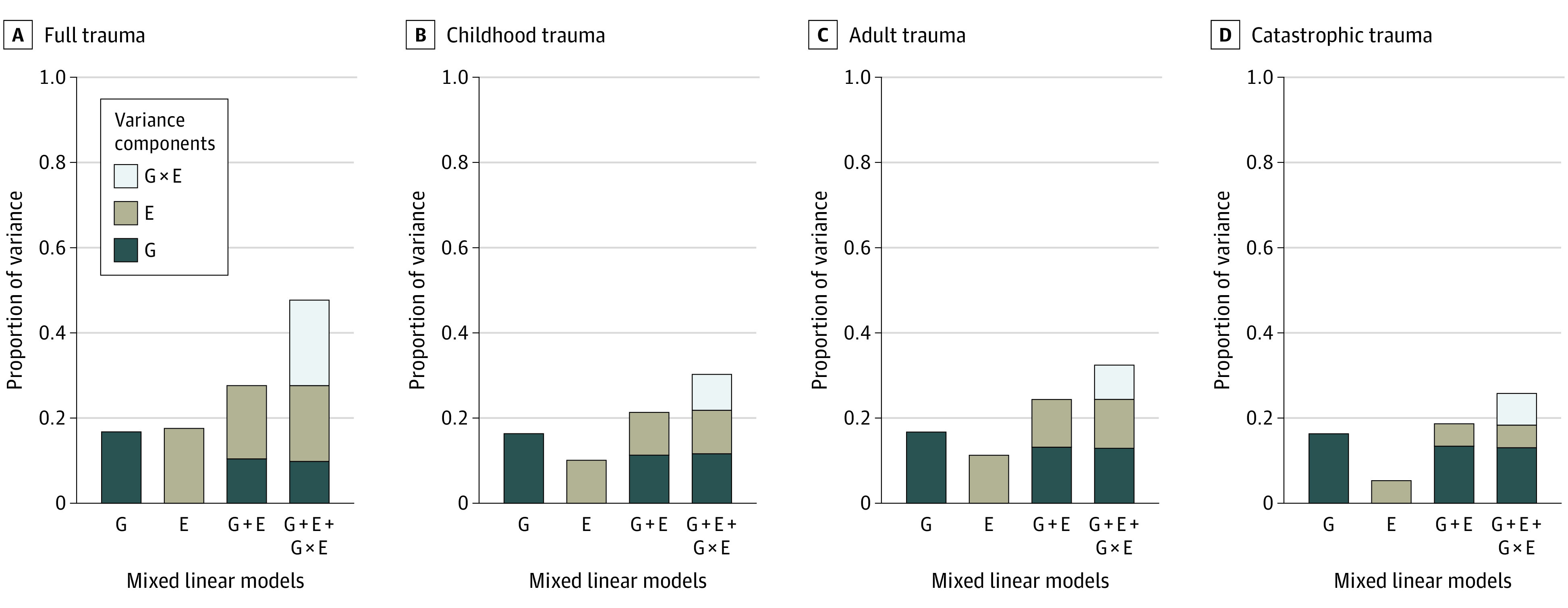
Proportion of Composite International Diagnostic Inventory (CIDI) Depression Variance Explained by Genetic, Environmental, and Interaction Sources in UK Biobank The proportion of variance explained is specified on the y-axis. The sources of variation are genomic (G), environmental (E), and gene-by-environment interactions (G × E), and their corresponding models are specified on the x-axis. E represents trauma exposure. Facets show the different trauma exposures explored.

Figure 1 shows the estimates for the proportion of CIDI depression variance explained by the different sources included in the mixed linear models (results are the meta-analysis of the 5 UKB subsamples). All estimates for proportion of variance explained by all components were statistically significant. Log-likelihood ratio tests (LRTs) suggested that the inclusion of trauma (E) and genome-by-trauma (G × E) interaction components improve model fit. Full details of these analyses, including estimates, SEs, LRT values, as well as results using broad depression and neuroticism as dependent variables, can be found in eTables 35-38 in the Supplement.

Heritability (SE) estimates (ie, proportion of phenotypic variance accounted for by the G) of CIDI depression were stable across the different UKB subsamples, approximately 0.16 (0.016). The meta-analyzed estimates (SE) for proportion of variance attributable to trauma exposure (Es) were 0.18 (0.025) for full trauma, 0.101 (0.027) for childhood trauma, 0.113 (0.03) for adult trauma, 0.05 (0.013) for catastrophic trauma. The meta-analyzed estimates (SE) for proportion of variance attributable to the interaction effect (G × E) were highest when exploring full trauma 0.201 (0.009) and were 0.084 (0.006), 0.081 (0.005), and 0.074 (0.006) when exploring childhood, adult, and catastrophic trauma separately, respectively.

Similar results were obtained when using only unrelated individuals as well as when mixed linear models used Es computed from trauma eigenvectors precorrected for the full sample G (eTables 39-40 in the Supplement). In contrast, although model fit, compared with models excluding Es, was significantly improved, we observed smaller estimates and LRT values when mixed linear models used Es computed from only PC1 of full trauma items (eTable 41 in the Supplement). Significant, smaller estimates of variance components were observed for broad depression and neuroticism (Tables 35-38 in the Supplement).

**Figure 2.**
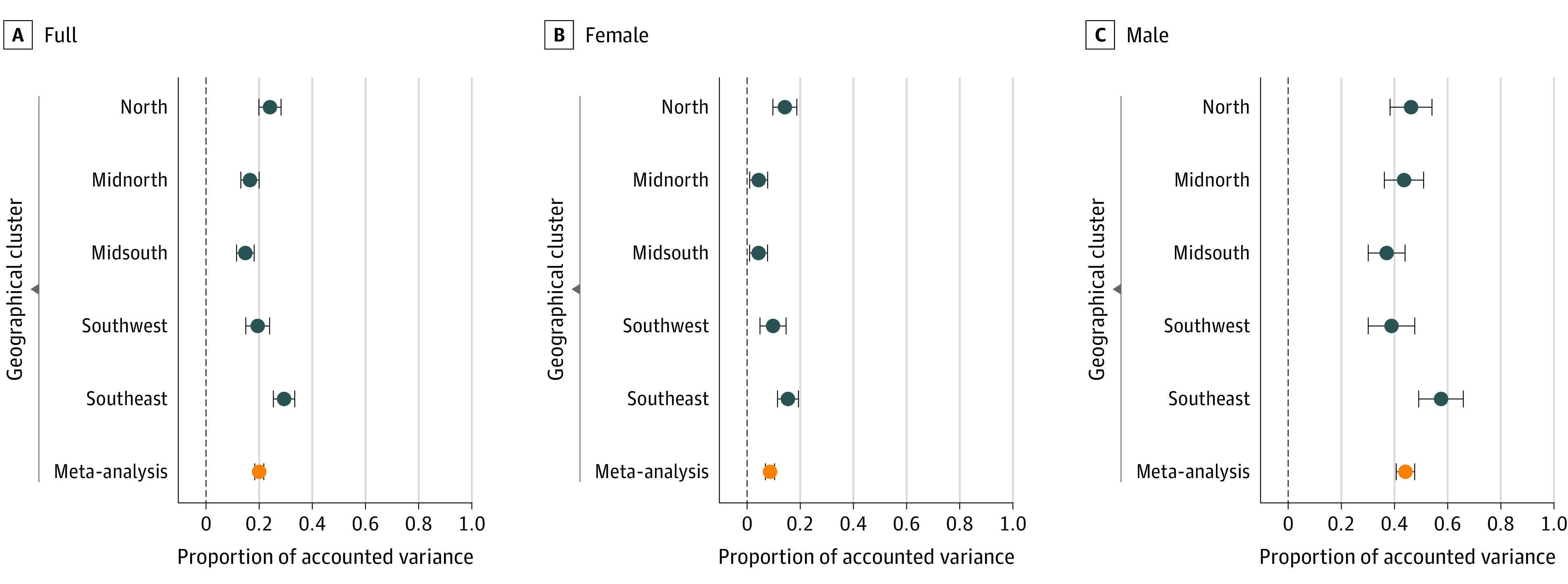
Proportion of Composite International Diagnostic Inventory (CIDI) Depression Variation Explained by the Genome-by-Trauma Interactions Forest plot x-axis shows proportion of CIDI depression variance explained by the genome-by-trauma interaction effect with bars representing SEs. Results for geographic clusters (blue) and meta-analyzed estimates (orange) are shown on the y-axis. Facets represent the analysis results using different samples.

Figure 2 shows the estimates for the proportion of CIDI depression variance explained by the interaction (G × E) included in the mixed linear models. Here, the interaction matrix used Es capturing full trauma exposure. Results for each geographic cluster as well as within female/male participant–only samples are presented. Full details of these analyses, including estimates, SEs, LRTs values as well as results using broad depression and neuroticism as the dependent variable, can be found in the Supplement (eTables 35, 42, 43 in the Supplement).

Meta-analyzed estimates for proportion of CIDI depression variance explained by G × E interactions across the clusters were statistically significant within the whole (joint female and male participants), female, and male samples. Compared with the analyses of the full sample (joint male and female participants), the meta-analyzed interaction variance was smaller when explored within the female sample and larger when explored within the male sample. Similar results were observed when using only unrelated individuals (eTables 44-45 in the Supplement).

## Discussion

In this cross-sectional study, results suggest contributions of genome-by-trauma interactions to depression/neuroticism phenotypic variation that are of relatively large magnitude (7%-20%), approximately the same magnitude as the variance captured by self-reported trauma exposure itself (5%-18%). Exploring variance components of depression/neuroticism within male and female individuals separately indicates that the proportion of CIDI depression variation captured by genome-by-trauma interactions differs substantially between the sexes, with estimates being approximately 5-fold greater in male individuals.

To more comprehensively capture genome-by-trauma interactions within depression and neuroticism, the approach uses all genotyped variants to compute genetic similarity instead of individual SNVs or PGSs as in some previous studies.^16,19,27^ We computed trauma exposure and genome-by-trauma interaction similarity to explore trait variance attributable to these effects by incorporating the genetic, trauma exposure, and interaction terms as random effects as opposed to fixed effects within linear models.^19,20,27^ Moreover, we used all related individuals, with appropriate sensitivity analyses (limited to unrelated individuals only). By using all available genotyped data, the mixed linear models implemented have greater statistical power to identify phenotypic variation attributable to genetic and genome-by-trauma interactions. Hence, this method is able to uncover a greater proportion of trait variance than observed with individual SNVs or PGSs. We explored general (full trauma) and specific (childhood, adult, catastrophic) measures of trauma.

Our heritability estimates of the trauma measures support findings from the literature.^46^ Our results suggest statistically significant and modest genetic correlations between the trauma and depression/neuroticism variables. Genetic correlations are 2-fold greater with CIDI depression as opposed to broad depression and neuroticism. A perception component of these traits, ie, the more extreme one is on the depression scale the more likely one is to either remember or perceive an event as traumatic, may explain these genetic correlations. This caveat should also be considered and curb interpretation of the causal role of trauma exposure in depression manifestation. Future research that can combine longitudinal designs with prospective measures will be able to greatly enhance this line of research.

Our results provide depression/neuroticism heritability estimates and estimates for proportion of variance attributable to self-reported trauma exposure in line with previous literature.^47,48,49^ Findings also suggest the subcategories, childhood, adult, and catastrophic, trauma explain a substantial proportion of CIDI depression variance.

As mentioned previously, we observed significant contributions of genome-by-trauma interactions to depression/neuroticism phenotypic variation. This finding was consistent (although estimates are lower) across the subcategories (childhood, adult and catastrophic) of trauma exposure (Figure 1). Lower, yet still significant, interaction estimates were observed for broad depression and neuroticism phenotypes, except for the nonsignificant genome-by-catastrophic trauma interaction estimate within broad depression (eTable 38 in the Supplement). The contrast between results exploring CIDI depression, broad depression, and neuroticism may highlight that genome-by-trauma interaction effects play a more specific role within MDD manifestation.

Our findings suggest that model fit was significantly improved when mixed linear models used environmental relationship (Es) and genome-by-trauma interaction (G × E) matrices. This was also observed when Es were computed using only the first eigenvector of trauma items. However, the variance components estimates were substantially attenuated when compared with results from models including Es computed using all PCs of trauma items. This suggests that important trauma exposure and genome-by-trauma interactions may be distributed across the different dimensions of self-reported trauma exposure. The inclusion of more self-reported trauma exposure PCs may additionally capture the differential impact of subtypes of trauma exposure.

The substantial difference between the sexes in the proportion of depression variation captured by genome-by-trauma interactions was also observed for broad depression and neuroticism phenotypes. These results, alongside the evident prevalence differences, highlight the importance and need to explore these associations within the sexes separately. Our findings suggest that trauma exposure and sensitivity to trauma exposure accounted for greater variance in depression/neuroticism outcomes for male individuals.

Although using PCs enables the use of all trauma exposure variables, it is difficult to interpret directions of associations as higher PC values do not necessarily mean higher levels of trauma exposure. Further, research can be conducted to explore the direction of these associations. Exploring individual trauma (neglect, physical abuse, etc) measures may provide a better understanding of the effect of specific trauma and genome-by-trauma experiences.

Our findings suggest evidence of differential associations between trauma exposure dependent on differences in individual genetic liability with depression. Our research design and analyses are repeated across 5 geographic cluster samples. These within-sample replications, although not independent samples, yielded relatively consistent estimates and SEs, thereby increasing confidence in our results. It is evident that the method employed here has major advantages when exploring genome-by-trauma exposure interactions as opposed to much of the literature making use of PGSs.^16,20,26,30^

### Limitations

There are limitations to this study that need to be considered when interpreting results. Hence, results of this study may not be generalisable to the whole population.Although the Gs computed used all genotyped SNVs and subsequently accounted for greater variance than PGSs, discrepancies between twin study heritability and SNV heritability estimates of depression were still apparent. Twin study estimates may be biased upward owing to the presence of gene-environment interplay effects, and thus, real heritability estimates are likely to fall between SNV heritability and twin study heritability estimates.^50^ Moreover, results show that our environmental variables, full trauma and the subcategories of trauma, have moderate heritability estimates, and these are genetically correlated with our outcome phenotypes (depression/neuroticism). This highlights that our environmental measures captured both genetic and environmental variances. As genomic relationship matrices are not capturing the entirety of the genetic variance within depression/neuroticism outcomes, the variance captured by the trauma (and subtrauma) measures may capture residual genetic variance.

To control for genetic covariance between our environmental and outcome variables, we also explored measures of trauma precorrected for the available genetic measure (Gs). The differences in estimates of variance components were negligible (eTables 35 and 40 in the Supplement). However, similar to the aforementioned limitation, this effect can be more accurately controlled for with an improved genetic measure (eg, G using imputed or whole-genome sequenced data). Simulation findings from the literature suggest that making use of imputed or whole-genome sequencing genetic data for Gs can uncover a further substantial proportion of genetic variance,^50^ which would be useful in addressing the limitations outlined previously. Moreover, future work could entail the simulation of correlated genetic and environmental data with absent G × E associations, to explore how this would affect the mixed linear model results, particularly the variance attributable to G × E.

The UK Biobank study is a homogeneous cohort with a healthy volunteer bias, which means that participants tend to have relatively better health and higher socioeconomic status.^51^ Moreover, our trauma exposure, depression, and neuroticism variables were measured using retrospective self-report. Furthermore, measures of trauma exposure and CIDI depression were obtained later than measures of broad depression and neuroticism with the follow-up UKB mental health questionnaire. This indicates potential measurement error within our variables.^16^ Incorporating more objective measures of trauma exposure, eg, omics measures (DNA methylation) may be able to provide a measure of trauma exposure that is less susceptible to reporting bias and thus, measurement error. For instance, the availability of methylation data has increased substantially and can be used as good proxy measures of environments as seen with smoking.^35,52^ Evidence suggests there may be a methylation profile associated with trauma exposure.^53,54^ Genome-by-environment interaction effects using methylation data can then be dissected to explore biologic pathways with nonadditive effects on outcomes that can be directly targeted. Findings could also further clarify the relationship between genetic liability and trauma exposure.

## Conclusions

In conclusion, findings of this cross-sectional study suggest empirical evidence of depression/neuroticism variation associated with genome-by-trauma interactions. The magnitude of these associations were much larger for male individuals than for female individuals. These findings can be further explored to identify both risk groups and modifiable environments/biological pathways that yield greater risk of depression manifestation, which would be useful in personalized/preventive interventions.

## Notes

Supplement. **eMethods. **Participants, Genotypes, Phenotypes **eTable 1.** Sample Sizes of UKB Participants With Complete Trauma, Depression, and Neuroticism Information **eFigure 1.** Trauma Exposure Distribution by Sex **eTable 2.** Broad Depression UK Biobank Questions and Field Codes **eTable 3.** Neuroticism UK Biobank Questions and Field Codes **eTable 4.** Composite International Diagnostic Inventory (CIDI) Depression UK Biobank Questions and Field Codes **eTable 5.** Exclusion Criteria UK Biobank Questions and Field Codes **eTable 6.** Childhood Trauma Exposure and CIDI Depression Regression Model Associations **eTable 7.** Childhood Trauma Exposure and Broad Depression Regression Model Associations **eTable 8.** Childhood Trauma Exposure and Neuroticism Regression Model Associations **eTable 9.** Adult Trauma Exposure and CIDI Depression Regression Model Associations **eTable 10.** Adult Trauma Exposure and Broad Depression Regression Model Associations **eTable 11.** Adult Trauma Exposure and Neuroticism Regression Model Associations **eTable 12.** Catastrophic Trauma Exposure and CIDI Depression Regression Model Associations **eTable 13.** Catastrophic Trauma Exposure and Broad Depression Regression Model Associations **eTable 14.** Catastrophic Trauma Exposure and Neuroticism Regression Model Associations **eTable 15.** Childhood Trauma Exposure Principal Components and Depression/Neuroticism Regression Model Associations **eTable 16.** Adult Trauma Exposure Principal Components and Depression/Neuroticism Regression Model Associations **eTable 17.** Catastrophic Trauma Exposure Principal Components and Depression/Neuroticism Regression Model Associations **eTable 18.** Full Trauma Exposure Principal Components and Depression/Neuroticism Regression Model Associations **eTable 19.** Childhood Trauma Exposure Principal Component Loadings **eTable 20.** Adult Trauma Exposure Principal Component Loadings **eTable 21.** Catastrophic Trauma Exposure Principal Component Loadings **eTable 22.** Full Trauma Exposure Principal Component Loadings **eFigure 2.** UK Biobank Geographical Clusters **eTable 23.** Cluster Demographics Using All Participants With Complete Trauma Exposure Responses **eTable 24.** Cluster Demographics Using Unrelated Participants With Complete Trauma Exposure Responses **eTable 25.** Cluster Demographics Using All Participants With Complete Childhood Trauma Exposure Responses **eTable 26.** Cluster Demographics Using All Participants With Complete Adult Trauma Exposure Responses **eTable 27.** Cluster Demographics Using All Participants With Catastrophic Trauma Exposure Responses **eTable 28.** Cluster Demographics Using All Female Participants With Complete Trauma Exposure Responses **eTable 29.** Cluster Demographics Using Unrelated Female Participants With Complete Trauma Exposure Responses **eTable 30.** Cluster Demographics Using All Male Participants With Complete Trauma Exposure Responses **eTable 31.** Cluster Demographics Using Unrelated Male Participants With Complete Trauma Exposure Responses
eAppendix 1.
**eTable 32.** Mixed Linear Model Results of Proportion of CIDI Depression Variance Attributable to Environmental Relationship Matrices Computed Using Different OSCA Algorithms **eTable 33.** Trauma Exposure Heritability Estimates **eTable 34.** Trauma Exposure and Depression/Neuroticism Genetic Correlations **eTable 35.** Mixed Linear Model Results Including Es of Full Trauma Exposure Principal Components **eTable 36.** Mixed Linear Model Results Including Es of Childhood Trauma Exposure Principal Components **eTable 37.** Mixed Linear Model Results Including Es of Adult Trauma Exposure Principal Components **eTable 38.** Mixed Linear Model Results Including Es of Full Catastrophic Trauma Exposure Principal Components **eTable 39.** Mixed Linear Model Results Including Es of Full Trauma Exposure Principal Components Using Unrelated Individuals **eTable 40.** Mixed Linear Model Results Including Es of Full Trauma Exposure Principal Components Precorrected for Genomic Relationship Matrix **eTable 41.** Mixed Linear Model Results Including Es of Full Trauma Exposure Principal Component 1 **eTable 42.** Mixed Linear Model Results Including Es of Female Only Full Trauma Exposure Principal Components **eTable 43.** Mixed Linear Model Results Including Es of Male Only Full Trauma Exposure Principal Components **eTable 44.** Mixed Linear Model Results Including Es of Female Only Full Trauma Exposure Principal Components Using Unrelated Individuals **eTable 45.** Mixed Linear Model Results Including Es of Male Only Full Trauma Exposure Principal Components Using Unrelated Individuals **eTable 46.** Mixed Linear Model Results Including Es of Full Trauma Exposure Principal Components; Varying CIDI Depression Prevalence Rates
eAppendix 2.

eReferences
