# Supplementary material for: Genome-by-Trauma Exposure Interactions in Adults With Depression in the UK Biobank
Source: JAMA Psychiatry. 2022 Sep 28;79(11):1110–7. doi: 10.1001/jamapsychiatry.2022.2983 (PMC9520433; doi:10.1001/jamapsychiatry.2022.2983)
Supplement: Supplement. — eMethods. Participants, Genotypes, Phenotypes eTable 1. Sample Sizes of UKB Participants With Complete Trauma, Depression, and Neuroticism Information eFigure 1. Trauma Exposure Distribution by Sex eTable 2. Broad Depression UK Biobank Questions and Field Codes eTable 3. Neuroticism UK Biobank Questions and Field Codes eTable 4. Composite International Diagnostic Inventory (CIDI) Depression UK Biobank Questions and Field Codes eTable 5. Exclusion Criteria UK Biobank Questions and Field Codes eTable 6. Childhood Trauma Exposure and CIDI Depression Regression Model Associations eTable 7. Childhood Trauma Exposure and Broad Depression Regression Model Associations eTable 8. Childhood Trauma Exposure and Neuroticism Regression Model Associations eTable 9. Adult Trauma Exposure and CIDI Depression Regression Model Associations eTable 10. Adult Trauma Exposure and Broad Depression Regression Model Associations eTable 11. Adult Trauma Exposure and Neuroticism Regression Model Associations eTable 12. Catastrophic Trauma Exposure and CIDI Depression Regression Model Associations eTable 13. Catastrophic Trauma Exposure and Broad Depression Regression Model Associations eTable 14. Catastrophic Trauma Exposure and Neuroticism Regression Model Associations eTable 15. Childhood Trauma Exposure Principal Components and Depression/Neuroticism Regression Model Associations eTable 16. Adult Trauma Exposure Principal Components and Depression/Neuroticism Regression Model Associations eTable 17. Catastrophic Trauma Exposure Principal Components and Depression/Neuroticism Regression Model Associations eTable 18. Full Trauma Exposure Principal Components and Depression/Neuroticism Regression Model Associations eTable 19. Childhood Trauma Exposure Principal Component Loadings eTable 20. Adult Trauma Exposure Principal Component Loadings eTable 21. Catastrophic Trauma Exposure Principal Component Loadings eTable 22. Full Trauma Exposure Principal Component Loadings eFigure 2. UK Biobank Ge [file jamapsychiatry-e222983-s001.pdf]

**Supplementary Online Content**

Chuong M, Adams MJ, Kwong ASF, Haley CS, Amador C, McIntosh AM. Genome-by-trauma exposure interactions in adults with depression in the UK Biobank. *JAMA Psychiatry*. Published online September 28, 2022. doi:10.1001/jamapsychiatry.2022.2983

**eMethods.** Participants, Genotypes, Phenotypes
**eTable 1.** Sample Sizes of UKB Participants With Complete Trauma, Depression, and Neuroticism Information
**eFigure 1.** Trauma Exposure Distribution by Sex
**eTable 2.** Broad Depression UK Biobank Questions and Field Codes
**eTable 3.** Neuroticism UK Biobank Questions and Field Codes
**eTable 4.** Composite International Diagnostic Inventory (CIDI) Depression UK Biobank Questions and Field Codes
**eTable 5.** Exclusion Criteria UK Biobank Questions and Field Codes
**eTable 6.** Childhood Trauma Exposure and CIDI Depression Regression Model Associations
**eTable 7.** Childhood Trauma Exposure and Broad Depression Regression Model Associations
**eTable 8.** Childhood Trauma Exposure and Neuroticism Regression Model Associations
**eTable 9.** Adult Trauma Exposure and CIDI Depression Regression Model Associations
**eTable 10.** Adult Trauma Exposure and Broad Depression Regression Model Associations
**eTable 11.** Adult Trauma Exposure and Neuroticism Regression Model Associations
**eTable 12.** Catastrophic Trauma Exposure and CIDI Depression Regression Model Associations
**eTable 13.** Catastrophic Trauma Exposure and Broad Depression Regression Model Associations
**eTable 14.** Catastrophic Trauma Exposure and Neuroticism Regression Model Associations
**eTable 15.** Childhood Trauma Exposure Principal Components and Depression/Neuroticism Regression Model Associations
**eTable 16.** Adult Trauma Exposure Principal Components and Depression/Neuroticism Regression Model Associations
**eTable 17.** Catastrophic Trauma Exposure Principal Components and Depression/Neuroticism Regression Model Associations
**eTable 18.** Full Trauma Exposure Principal Components and Depression/Neuroticism Regression Model Associations
**eTable 19.** Childhood Trauma Exposure Principal Component Loadings
**eTable 20.** Adult Trauma Exposure Principal Component Loadings
**eTable 21.** Catastrophic Trauma Exposure Principal Component Loadings
**eTable 22.** Full Trauma Exposure Principal Component Loadings
**eFigure 2.** UK Biobank Geographical Clusters
**eTable 23.** Cluster Demographics Using All Participants With Complete Trauma Exposure Responses
**eTable 24.** Cluster Demographics Using Unrelated Participants With Complete Trauma Exposure Responses
**eTable 25.** Cluster Demographics Using All Participants With Complete Childhood Trauma Exposure Responses
**eTable 26.** Cluster Demographics Using All Participants With Complete Adult Trauma Exposure Responses
**eTable 27.** Cluster Demographics Using All Participants With Catastrophic Trauma Exposure Responses
**eTable 28.** Cluster Demographics Using All Female Participants With Complete Trauma Exposure Responses
**eTable 29.** Cluster Demographics Using Unrelated Female Participants With Complete Trauma Exposure Responses
**eTable 30.** Cluster Demographics Using All Male Participants With Complete Trauma Exposure Responses
**eTable 31.** Cluster Demographics Using Unrelated Male Participants With Complete Trauma Exposure Responses
**eAppendix 1.**
**eTable 32.** Mixed Linear Model Results of Proportion of CIDI Depression Variance Attributable to Environmental Relationship Matrices Computed Using Different OSCA Algorithms
**eTable 33.** Trauma Exposure Heritability Estimates
**eTable 34.** Trauma Exposure and Depression/Neuroticism Genetic Correlations
**eTable 35.** Mixed Linear Model Results Including Es of Full Trauma Exposure Principal Components
**eTable 36.** Mixed Linear Model Results Including Es of Childhood Trauma Exposure Principal Components
**eTable 37.** Mixed Linear Model Results Including Es of Adult Trauma Exposure Principal Components

© 2022 Chuong M et al. *JAMA Psychiatry.*

**eTable 38.** Mixed Linear Model Results Including Es of Full Catastrophic Trauma Exposure Principal Components
**eTable 39.** Mixed Linear Model Results Including Es of Full Trauma Exposure Principal Components Using Unrelated Individuals
**eTable 40.** Mixed Linear Model Results Including Es of Full Trauma Exposure Principal Components Precorrected for Genomic Relationship Matrix
**eTable 41.** Mixed Linear Model Results Including Es of Full Trauma Exposure Principal Component 1
**eTable 42.** Mixed Linear Model Results Including Es of Female Only Full Trauma Exposure Principal Components
**eTable 43.** Mixed Linear Model Results Including Es of Male Only Full Trauma Exposure Principal Components
**eTable 44.** Mixed Linear Model Results Including Es of Female Only Full Trauma Exposure Principal Components Using Unrelated Individuals
**eTable 45.** Mixed Linear Model Results Including Es of Male Only Full Trauma Exposure Principal Components Using Unrelated Individuals
**eTable 46.** Mixed Linear Model Results Including Es of Full Trauma Exposure Principal Components; Varying CIDI Depression Prevalence Rates
**eAppendix 2.**
**eReferences**

This supplementary material has been provided by the authors to give readers additional information about their work.

© 2022 Chuong M et al. *JAMA Psychiatry.*

**eMethods.** Participants, Genotypes, Phenotypes

## Participants

We used data from the UK Biobank (UKB), a national study exploring genetic and environmental determinants of health using individuals recruited from 22 different centres across the United Kingdom.[1,2] A follow up Mental Health Questionnaire (MHQ) was administered assessing common mental health disorders including trauma experience.[3] In this study we had access to individual level data from 148 129 participants who completed the MHQ with available genetic, trauma experience, depressive symptoms and/or neuroticism information. Data were limited to individuals with White British ethnicity and participants who requested their data be removed, were not included in analyses.

The UKB study received ethical approval from the NHS National Research Ethics Service (reference: 11/NW/0382) and all participants provided written informed consent. This study has been approved by the UKB Access Committee (Project #4844).

## Genotypes

Two separate array chips with 95% SNP overlap, were used to genotype UKB participants; the Applied Biosystems™ UK BiLEVE Axiom™ Array by Affymetrix1 captures 807 411 SNPs and the Applied Biosystems™ UK Biobank Axiom™ Array captures 825 927 SNPs.[4] Quality control of genotyped SNPs consisted of exclusion of SNPs with missingness > 2% and a Hardy-Weinberg Equilibrium test $p < 10^{-6}$. SNPs with minor allele frequency (MAF) < 0.05 and individuals with > 2% missing genotypes were excluded from analyses. A total of 414 584 common SNPs across the 22 autosomes were included in analyses.

## Phenotypes

All phenotypes were defined using retrospective self-reported responses to questions assessing help-seeking behaviour, depressive symptoms, and trauma experience. Field codes and information on classifications are available (eTables 2-5,6,9,12).

### Depression & Neuroticism Phenotypes

CIDI depression was defined using questions from the Composite International Diagnostic Interview Short Form (CIDI).[5] These were administered within the online follow-up Mental Health Questionnaire (MHQ), directly assessing the Diagnostic and Statistical Manual of Mental Disorders, Fifth Edition (DSM-5)[6] criteria for MDD, and are associated with greater MDD specificity.[7]

Supplementary analyses were conducted using broad depression and neuroticism. Broad depression was defined using self-reported help seeking behaviour. Case and control status was determined from the response to Touchscreen Questionnaire questions administered during initial recruitment; ‘Have you ever seen a general practitioner (GP) for nerves, anxiety, tension or depression?’ or ‘Have you ever seen a psychiatrist for nerves, anxiety, tension or depression? Individuals responding ‘Yes’ to either and ‘No’ to both questions were classified as cases and controls, respectively.

Neuroticism was measured using the 12-item Neuroticism scale of the revised short form Eysenck Personality Questionnaire (EPQ-R).[8] Response options were ‘Yes’, ‘No’, ‘Do not know’, ‘Prefer not to answer’. Summed scores of ‘Yes’ responses (ranging from 0-12) were used in analyses.

### Trauma Phenotypes

© 2022 Chuong M et al. *JAMA Psychiatry.*

Participants were administered a 16-item questionnaire relating to traumatic experiences as a part of the MHQ. Five items explored childhood trauma using the Childhood Trauma Screener[9,10]; five items explored adult trauma using an equivalent screener developed by the UKB Mental Health steering group[11]; and six items explored catastrophic trauma using questions related to events that often trigger post-traumatic stress disorder.[12] More information on questions, distribution of responses can be found in eFigure 1 and eTables 6-14. **eTable 1** shows samples sizes of individuals included with complete trauma exposure, depression and neuroticism information.

Individual regression analyses between each trauma item and depression/neuroticism outcomes, with age and sex as covariates (eTables 6-14), suggested most trauma items to be significantly associated with the three phenotypes. Hence, all items were utilised in the formation of trauma phenotypes.

It may seem intuitive that certain forms of trauma exposure should have greater weighting when examining its role in depression manifestation e.g. sexual/physical or neglect [13,14]. However, determining the weights for specific trauma exposures can be difficult as many trauma subtypes are overlapping and correlated with one another. [15] In order to capture separate and independent dimensions of trauma, principal components (PCs) of complete responses to all trauma (full trauma) as well as the three sub-categories of trauma items (childhood, adult, catastrophic trauma) were obtained. Trauma items were rescaled to have a mean of one and unit variance before PCs were extracted using the 'prcomp' function in R 4.0.2.[16] For more information on depression/neuroticism associations with trauma PCs and PC loadings see eTables 15-22.

**eTable 1**. Sample Sizes of UKB Participants With Complete Trauma, Depression, and Neuroticism Information

| **Trauma Measure** | **CIDI** | | **Broad** | | **Neuroticism** |
|---|---|---|---|---|---|
| | **Case** | **Control** | **Case** | **Control** | |
| **FULL TRAUMA** | 32 015 | 81 809 | 45 093 | 82 370 | 116 995 |
| **CHILDHOOD** | 33 532 | 85 405 | 47 583 | 85 894 | 121 670 |
| **ADULT** | 33 262 | 83 722 | 46 886 | 84 234 | 119 914 |
| **CATASTROPHIC** | 33 657 | 85 704 | 47 850 | 86 127 | 122 108 |

*Abbreviations.* UKB, UK Biobank; CIDI, Composite International Diagnostic Inventory diagnosis criteria of Major Depressive Disorder (MDD); Broad, diagnosis of MDD based on self-reported diagnosis of nerves, anxiety, tensions or depression from healthcare professional.

© 2022 Chuong M et al. *JAMA Psychiatry.*

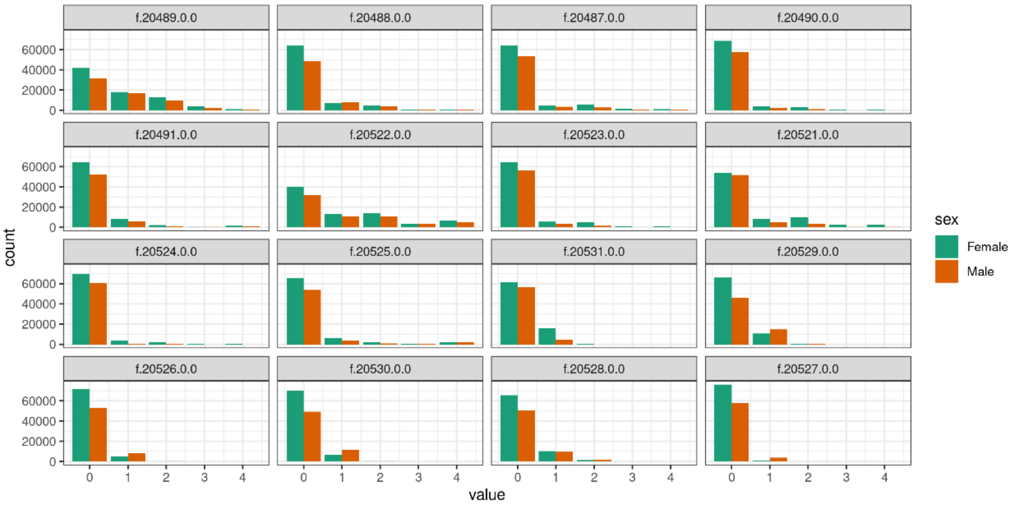


**eFigure 1.** Trauma Exposure Distribution by Sex. Facets represent the 16 different trauma exposure questions (field codes) available within the UK Biobank Mental Health Questionnaire. The x-axis represents the available responses '0 - Never True; 1- Rarely True; 2 – Sometimes True; 3 – Often True; 4 – Always True'. The questions related to catastrophic trauma exposure responses were '0 – Never True; 1 – Yes, not within 12 months; 2 – Yes, within 12 months'. Each field code and corresponding question can be found in table 2 of this appendix.

© 2022 Chuong M et al. *JAMA Psychiatry.*

**eTable 2.** Broad Depression UK Biobank Questions and Field Codes

| UKB FIELD | QUESTION | RESPONSE OPTIONS | CRITERIA |
|---|---|---|---|
| f.2090.0.0 | Seen doctor (GP) for nerves, anxiety, tension or depression | Do not know<br>Prefer not to answer<br>No<br>Yes | Participants who responded 'Yes' to either question are classified as cases. Participants who repsonded 'No' to both questions are classified as controls. Participants who responded 'Prefer not to answer' or 'Do not know' were omitted from further analyses. |
| f.2100.0.0 | Seen a psychiatrist for nerves, anxiety, tension or depression | | |

**eTable 3.** Neuroticism UK Biobank Questions and Field Codes

| UKB FIELD | QUESTION | RESPONSE OPTIONS | CRITERIA |
|---|---|---|---|
| f.1920.0.0 | Does your mood often go up and down? | Do not know<br>Prefer not to answer<br>No<br>Yes | Participants are given a sumscore of neuroticism symptom between 0-12 |
| f.1930.0.0 | Do you ever feel 'just miserable' for no reason? | | |
| f.1940.0.0 | Are you an irritable person? | | |
| f.1950.0.0 | Are your feelings easily hurt? | | |
| f.1960.0.0 | Do you often feel 'fed-up'? | | |
| f.1970.0.0 | Would you call yourself a nervous person? | | |
| f.1980.0.0 | Are you a worrier? | | |
| f.1990.0.0 | Would you call yourself tense or 'highly strung'? | | |
| f.2000.0.0 | Do you worry too long after an embarrassing experience? | | |
| f.2010.0.0 | Do you suffer from 'nerves'? | | |
| f.2020.0.0 | Do you often feel lonely? | | |
| f.2030.0.0 | Are you often troubled by feelings of guilt? | | |

© 2022 Chuong M et al. *JAMA Psychiatry.*

**eTable 4.** Composite International Diagnostic Inventory (CIDI) Depression UK Biobank Questions and Field Codes

| UKB FIELD | QUESTION | RESPONSE OPTIONS | CRITERIA |
|---|---|---|---|
| f.20446.0.0 (cardinal) | Ever had prolong feelings of sadness or depression | Prefer not to answer<br>No<br>Yes | Participants who reported at least 5 symptoms, including one cardinal symptom AND reported 'Most of the day' or more affected during worst episode of depression [f20436], Depressed 'almost every day' or more during worst episode of depression [f20439] and 'More than a little impact' or more on normal roles during worst period of depression [f20440] were classified as cases. All other participants were classified as controls. |
| f.20441.0.0 (cardinal) | Ever had prolonged loss of interest in normal activities | | |
| f.20449.0.0 | Feelings of tiredness during worst episode of depression | | |
| f.20532.0.0 | Sleep change during worst episode of depression | | |
| f.20435.0.0 | Difficulty concentrating during worst episode of depression | | |
| f.20450.0.0 | Feelings of worthlessness during worst episode of | | |
| f.20437.0.0 | Thoughts of death during worst episode of depression | | |
| f.20536.0.0 | Weight change during worst episode of depression | Do not know<br>Prefer not to answer<br>Same/Dieting<br>Gained<br>Lost<br>Gained and lost | |
| f.20436.0.0 | Most of the day or more affected during worst episode of depression | Do not know<br>Prefer not to answer<br>Less than half of the day<br>About half of the day<br>Most of the day<br>All day long | |
| f.20439.0.0 | Depressed (almost) every day during worst episode of depression | Do not know<br>Prefer not to answer<br>Less often<br>Almost every day<br>Every day | |
| f.20440.0.0 | More than a little impact on normal roles during worst period of depression | Prefer not to answer<br>Not at all<br>A little<br>Somewhat<br>A lot | |

© 2022 Chuong M et al. *JAMA Psychiatry.*

**eTable 5.** Exclusion Criteria UK Biobank Questions and Field Codes

| UKB FIELD | QUESTION | RESPONSE OPTIONS | CRITERIA |
|---|---|---|---|
| f.20544.0.0 | Report any mental health problems diagnosed by a professional | Prefer not to answer<br>ADD/ADHD<br>Agoraphobia<br>Anorexia nervosa<br>Anxierty, nerves or GAD<br>Autism, Asperger's or ASD<br>Binge eating<br>Bulimia nervosa<br>Depression<br>Mania, hypomania, bipolar<br>OCD<br>Panic attacks<br>Any other phobia<br>Personality disorder<br>Any other type of psychosis<br>Schizophrenia<br>Social anxiety or social phobia | Cases self-reporting diagnoses of schizophrenia, other psychoses or bipolar disorder were excluded from analyses. Cases reporting any mental health disorder, hospitalisation due to mood disorders, taking antidepressant medication or met previouslt defined criteria for a mood disorder are exlcuded from analyses. |
| f.20002.0.0 | Report depression in previous interview with psychiatric nurse | Depression | |
| f.20126.0.0 | Meet previous criteria for depression or bipolar disorder | No bipolar or depression<br>Bipolar I disorder<br>Bipolar II disorder<br>Probable recurrent major depression<br>Single probable major depression | |
| f.41202.0.0<br>f.41204.0.0 | Have a hospital inpatient ICD10 code for mood disorder | F30-F39 | |
| f.20003.0.0 | Report use of anti-depressant medication* at baseline | 1140879616, 1140921600, 1140879540, 1140867878, 1140916282, 1140909806, 1140867888, 1141152732, 1141180212, 1140879634, 1140867876, 140882236, 1141190158, 1141200564, 1140867726, 1140879620, 1140867818, 1140879630, 1140879628, 1141151946, 1140867948, 1140867624, 1140867756, 1140867884, 1141151978, 1141152736, 1141201834, 1140867690, 1140867640, 1140867920, 1140867850, 1140879544, 1141200570, 1140867934, 1140867758, 1140867914, 1140867820, 1141151982, 1140882244, 1140879556, 1140867852, 1140867860, 1140917460, 1140867938, 1140867856, 1140867922, 1140910820, 1140882312, 1140867944, 1140867784, 1140867812, 1140867668, 1140867940 | |

© 2022 Chuong M et al. *JAMA Psychiatry.*

**eTables 6-14** present associations between UK Biobank trauma exposure questions and the phenotypes of interest; broad depression, CIDI depression and neuroticism. Trauma exposure question responses were ‘Rarely True’, ‘Sometimes True’, ‘Often True’, ‘Always True’. Trauma exposure was explored as a categorical variable, and covariates; age and sex, were included in analyses. eTables 6-14 were presented in separate sections for each trauma exposure sub-category (childhood, adult and catastrophic trauma exposure) and each phenotype of interest. Tables include β coefficient estimates of associations, standard errors, T and P-Values as well as phenotypic variance accounted for by each model.

© 2022 Chuong M et al. *JAMA Psychiatry.*

**eTable 6.** Childhood Trauma Exposure and CIDI Depression Regression Model Associations

| Trauma | Predictor | Est | SE | T | P | AdjR2 | Ncase | Ncontrol |
|---|---|---|---|---|---|---|---|---|
| *Felt loved f.20489.0.0* | Intercept | 0.691 | 0.009 | 75.093 | 0.000 | 0.084 | | |
| | Sex(M) | -0.151 | 0.002 | -60.453 | 0.000 | | | |
| | Age | -0.007 | 0.000 | -43.205 | 0.000 | | | |
| | Rarely True | 0.032 | 0.003 | 10.809 | 0.000 | | 31163 | 64492 |
| | Sometimes True | 0.174 | 0.004 | 48.808 | 0.000 | | 18948 | |
| | Often True | 0.289 | 0.006 | 46.798 | 0.000 | | 5292 | |
| | Always True | 0.354 | 0.011 | 32.234 | 0.000 | | 1583 | |
| *Hit hard* f.20488.0.0 | Intercept | 0.696 | 0.009 | 74.646 | 0.000 | 0.066 | | |
| | Sex(M) | -0.156 | 0.003 | -62.029 | 0.000 | | | |
| | Age | -0.007 | 0.000 | -39.996 | 0.000 | | | |
| | Rarely True | 0.077 | 0.004 | 18.975 | 0.000 | | 12954 | 99403 |
| | Sometimes True | 0.192 | 0.005 | 37.210 | 0.000 | | 7692 | |
| | Often True | 0.306 | 0.014 | 22.128 | 0.000 | | 1002 | |
| | Always True | 0.381 | 0.018 | 21.569 | 0.000 | | 613 | |
| *Felt hated by family member f.20487.0.0* | Intercept | 0.661 | 0.009 | 71.538 | 0.000 | 0.084 | | |
| | Sex(M) | -0.140 | 0.002 | -55.860 | 0.000 | | | |
| | Age | -0.006 | 0.000 | -38.304 | 0.000 | | | |
| | Rarely True | 0.121 | 0.005 | 22.864 | 0.000 | | 7113 | 103745 |
| | Sometimes True | 0.247 | 0.005 | 47.964 | 0.000 | | 7546 | |
| | Often True | 0.379 | 0.010 | 36.418 | 0.000 | | 1748 | |
| | Always True | 0.420 | 0.011 | 36.751 | 0.000 | | 1447 | |
| *Sexually molested f.20490.0.0* | Intercept | 0.723 | 0.009 | 77.618 | 0.000 | 0.058 | | |
| | Sex(M) | -0.143 | 0.003 | -56.056 | 0.000 | | | |
| | Age | -0.007 | 0.000 | -42.388 | 0.000 | | | |
| | Rarely True | 0.119 | 0.006 | 19.467 | 0.000 | | 5363 | 110669 |
| | Sometimes True | 0.185 | 0.007 | 24.952 | 0.000 | | 3585 | |
| | Often True | 0.321 | 0.019 | 17.224 | 0.000 | | 552 | |
| | Always True | 0.371 | 0.021 | 18.032 | 0.000 | | 453 | |
| *Someone to take to the doctor f.20491.0.0* | Intercept | 0.756 | 0.009 | 81.211 | 0.000 | 0.054 | | |
| | Sex(M) | -0.150 | 0.003 | -59.037 | 0.000 | | | |
| | Age | -0.007 | 0.000 | -45.083 | 0.000 | | | |
| | Rarely True | 0.051 | 0.004 | 12.240 | 0.000 | | 12565 | 102320 |
| | Sometimes True | 0.193 | 0.008 | 24.001 | 0.000 | | 3046 | |
| | Often True | 0.247 | 0.015 | 16.535 | 0.000 | | 871 | |
| | Always True | 0.036 | 0.009 | 3.891 | 0.000 | | 2362 | |

*Abbreviations.* CIDI, Composite International Diagnostic Inventory definition of depression; Sex(M), Male; Est, estimate; SE, standard error; T, T-Value; P, P-Value; AdjR2, adjusted $R^2$ value; n, sample size.

© 2022 Chuong M et al. *JAMA Psychiatry.*

**eTable 7.** Childhood Trauma Exposure and Broad Depression Regression Model Associations

| Trauma | Predictor | Est | SE | T | P | AdjR2 | Ncase | Ncontrol |
|---|---|---|---|---|---|---|---|---|
| *Felt loved f.20489.0.0* | Intercept | 0.482 | 0.009 | 50.787 | 0.000 | 0.0557 | | |
| | Sex(M) | -0.159 | 0.003 | -62.030 | 0.000 | | | |
| | Age | -0.002 | 0.000 | -10.883 | 0.000 | | 34817 | |
| | Rarely True | 0.030 | 0.003 | 9.817 | 0.000 | | 21700 | 71645 |
| | Sometimes True | 0.163 | 0.004 | 45.123 | 0.000 | | 6033 | |
| | Often True | 0.248 | 0.006 | 39.683 | 0.000 | | 1808 | |
| | Always True | 0.307 | 0.011 | 27.694 | 0.000 | | | |
| *Hit hard* f.20488.0.0 | Intercept | 0.494 | 0.010 | 51.409 | 0.000 | 0.0394 | | |
| | Sex(M) | -0.163 | 0.003 | -63.344 | 0.000 | | | |
| | Age | -0.001 | 0.000 | -8.652 | 0.000 | | | |
| | Rarely True | 0.058 | 0.004 | 13.988 | 0.000 | | 14461 | |
| | Sometimes True | 0.150 | 0.005 | 28.486 | 0.000 | | 8614 | 115923 |
| | Often True | 0.243 | 0.014 | 16.907 | 0.000 | | 1077 | |
| | Always True | 0.304 | 0.018 | 16.515 | 0.000 | | 655 | |
| *Felt hated by family member f.20487.0.0* | Intercept | 0.462 | 0.010 | 48.382 | 0.000 | 0.0521 | | |
| | Sex(M) | -0.149 | 0.003 | -58.208 | 0.000 | | | |
| | Age | -0.001 | 0.000 | -6.958 | 0.000 | | | |
| | Rarely True | 0.105 | 0.005 | 19.515 | 0.000 | | 8060 | |
| | Sometimes True | 0.208 | 0.005 | 39.833 | 0.000 | | 8617 | 115923 |
| | Often True | 0.308 | 0.011 | 28.759 | 0.000 | | 1942 | |
| | Always True | 0.337 | 0.012 | 28.669 | 0.000 | | 1602 | |
| *Sexually molested f.20490.0.0* | Intercept | 0.512 | 0.010 | 53.375 | 0.000 | 0.0362 | | |
| | Sex(M) | -0.152 | 0.003 | -58.681 | 0.000 | | | |
| | Age | -0.002 | 0.000 | -10.320 | 0.000 | | | |
| | Rarely True | 0.092 | 0.006 | 14.941 | 0.000 | | 6104 | |
| | Sometimes True | 0.159 | 0.007 | 21.236 | 0.000 | | 4105 | 123692 |
| | Often True | 0.252 | 0.019 | 13.482 | 0.000 | | 637 | |
| | Always True | 0.294 | 0.021 | 13.884 | 0.000 | | 497 | |
| *Someone to take to the doctor f.20491.0.0* | Intercept | 0.541 | 0.010 | 56.586 | 0.000 | 0.0341 | | |
| | Sex(M) | -0.158 | 0.003 | -61.040 | 0.000 | | | |
| | Age | -0.002 | 0.000 | -13.003 | 0.000 | | | |
| | Rarely True | 0.043 | 0.004 | 10.250 | 0.000 | | 14154 | |
| | Sometimes True | 0.163 | 0.008 | 20.074 | 0.000 | | 3469 | 114362 |
| | Often True | 0.208 | 0.015 | 13.828 | 0.000 | | 991 | |
| | Always True | 0.050 | 0.009 | 5.449 | 0.000 | | 2665 | |

*Abbreviations.* Sex(M), Male; Est, estimate; SE, standard error; T, T-Value; P, P-Value; AdjR2, adjusted $R^2$ value; n, sample size.

© 2022 Chuong M et al. *JAMA Psychiatry.*

**eTable 8.** Childhood Trauma Exposure and Neuroticism Regression Model Associations

| Trauma | Predictor | Est | SE | T | P | AdjR2 | Ncase | Ncontrol |
|---|---|---|---|---|---|---|---|---|
| *Felt loved f.20489.0.0* | Intercept | 6.224 | 0.065 | 95.865 | 0.000 | 0.065 | | |
| | Sex(M) | -0.806 | 0.018 | -45.839 | 0.000 | | | |
| | Age | -0.043 | 0.001 | -37.757 | 0.000 | | | |
| | Rarely True | 0.411 | 0.021 | 19.482 | 0.000 | | 31162 | 65609 |
| | Sometimes True | 1.265 | 0.025 | 50.862 | 0.000 | | 19649 | |
| | Often True | 1.882 | 0.043 | 44.040 | 0.000 | | 5561 | |
| | Always True | 2.326 | 0.075 | 31.033 | 0.000 | | 1709 | |
| *Hit hard* f.20488.0.0 | Intercept | 6.423 | 0.066 | 97.244 | 0.000 | 0.038 | | |
| | Sex(M) | -0.825 | 0.018 | -46.302 | 0.000 | | | |
| | Age | -0.041 | 0.001 | -35.360 | 0.000 | | | |
| | Rarely True | 0.302 | 0.029 | 10.425 | 0.000 | | 13076 | |
| | Sometimes True | 0.922 | 0.036 | 25.622 | 0.000 | | 8042 | 101038 |
| | Often True | 1.542 | 0.097 | 15.895 | 0.000 | | 1034 | |
| | Always True | 1.588 | 0.121 | 13.123 | 0.000 | | 662 | |
| *Felt hated by family member f.20487.0.0* | Intercept | 6.153 | 0.066 | 93.790 | 0.000 | 0.053 | | |
| | Sex(M) | -0.738 | 0.018 | -41.679 | 0.000 | | | |
| | Age | -0.038 | 0.001 | -33.305 | 0.000 | | | |
| | Rarely True | 0.796 | 0.037 | 21.237 | 0.000 | | 7218 | |
| | Sometimes True | 1.399 | 0.036 | 38.806 | 0.000 | | 7882 | 105257 |
| | Often True | 2.021 | 0.072 | 28.205 | 0.000 | | 1881 | |
| | Always True | 2.101 | 0.079 | 26.694 | 0.000 | | 1556 | |
| *Sexually molested f.20490.0.0* | Intercept | 6.551 | 0.066 | 99.386 | 0.000 | 0.034 | | |
| | Sex(M) | -0.766 | 0.018 | -42.663 | 0.000 | | | |
| | Age | -0.043 | 0.001 | -36.928 | 0.000 | | | |
| | Rarely True | 0.383 | 0.043 | 8.990 | 0.000 | | 5596 | |
| | Sometimes True | 0.858 | 0.051 | 16.821 | 0.000 | | 3856 | 112289 |
| | Often True | 1.193 | 0.126 | 9.448 | 0.000 | | 609 | |
| | Always True | 1.642 | 0.142 | 11.594 | 0.000 | | 484 | |
| *Someone to take to the doctor f.20491.0.0* | Intercept | 6.712 | 0.066 | 102.226 | 0.000 | 0.035 | | |
| | Sex(M) | -0.791 | 0.018 | -44.267 | 0.000 | | | |
| | Age | -0.046 | 0.001 | -39.571 | 0.000 | | | |
| | Rarely True | 0.451 | 0.029 | 15.318 | 0.000 | | 12589 | |
| | Sometimes True | 0.994 | 0.056 | 17.681 | 0.000 | | 3148 | 104329 |
| | Often True | 1.511 | 0.102 | 14.853 | 0.000 | | 941 | |
| | Always True | 0.568 | 0.065 | 8.800 | 0.000 | | 2376 | |

*Abbreviations.* Sex(M), Male; Est, estimate; SE, standard error; T, T-Value; P, P-Value; AdjR2, adjusted $R^2$ value; n, sample size.

© 2022 Chuong M et al. *JAMA Psychiatry.*

**eTable 9.** Adult Trauma Exposure and CIDI Depression Regression Model Associations

| Trauma | Predictor | Est | SE | T | P | AdjR2 | Ncase | Ncontrol |
|---|---|---|---|---|---|---|---|---|
| *Been in a confiding relationship as an adult f.20522* | Intercept | 0.715 | 0.009 | 75.706 | 0.000 | 0.059 | | |
| | Sex(M) | -0.154 | 0.003 | -60.213 | 0.000 | | | |
| | Age | -0.007 | 0.000 | -42.679 | 0.000 | | | |
| | Rarely True | 0.037 | 0.003 | 10.489 | 0.000 | | 20921 | 61655 |
| | Sometimes True | 0.110 | 0.003 | 31.359 | 0.000 | | 20954 | |
| | Often True | 0.172 | 0.006 | 27.685 | 0.000 | | 5411 | |
| | Always True | 0.025 | 0.005 | 5.303 | 0.000 | | 9971 | |
| *Physical violence by partner or ex-partner as an adult f.20523.0.0* | Intercept | 0.694 | 0.009 | 74.613 | 0.000 | 0.067 | | |
| | Sex(M) | -0.135 | 0.003 | -53.316 | 0.000 | | | |
| | Age | -0.007 | 0.000 | -40.616 | 0.000 | | | |
| | Rarely True | 0.121 | 0.005 | 23.199 | 0.000 | | 7426 | 106872 |
| | Sometimes True | 0.211 | 0.006 | 34.379 | 0.000 | | 5316 | |
| | Often True | 0.290 | 0.015 | 19.850 | 0.000 | | 898 | |
| | Always True | 0.375 | 0.013 | 27.943 | 0.000 | | 1069 | |
| *Belittlement by partner or ex-partner as an adult f.20521.0.0* | Intercept | 0.624 | 0.009 | 67.840 | 0.000 | 0.100 | | |
| | Sex(M) | -0.113 | 0.003 | -44.950 | 0.000 | | | |
| | Age | -0.006 | 0.000 | -37.857 | 0.000 | | | |
| | Rarely True | 0.124 | 0.004 | 29.156 | 0.000 | | 11332 | 93821 |
| | Sometimes True | 0.242 | 0.004 | 56.669 | 0.000 | | 11455 | |
| | Often True | 0.377 | 0.009 | 44.246 | 0.000 | | 2611 | |
| | Always True | 0.452 | 0.009 | 50.437 | 0.000 | | 2368 | |
| *Sexual interference by partner or ex-partner without consent as an adult f.20524.0.0* | Intercept | 0.718 | 0.009 | 77.249 | 0.000 | 0.061 | | |
| | Sex(M) | -0.133 | 0.003 | -51.894 | 0.000 | | | |
| | Age | -0.007 | 0.000 | -42.200 | 0.000 | | | |
| | Rarely True | 0.172 | 0.007 | 23.281 | 0.000 | | 3657 | 115014 |
| | Sometimes True | 0.275 | 0.010 | 28.960 | 0.000 | | 2179 | |
| | Often True | 0.338 | 0.024 | 14.245 | 0.000 | | 339 | |
| | Always True | 0.449 | 0.022 | 20.014 | 0.000 | | 381 | |
| *Able to pay rent/mortgage as an adult f.20525.0.0* | Intercept | 0.728 | 0.009 | 77.961 | 0.000 | 0.060 | | |
| | Sex(M) | -0.146 | 0.003 | -57.404 | 0.000 | | | |
| | Age | -0.007 | 0.000 | -42.672 | 0.000 | | | |
| | Rarely True | 0.106 | 0.005 | 22.113 | 0.000 | | 8966 | 103689 |
| | Sometimes True | 0.274 | 0.008 | 32.599 | 0.000 | | 2791 | |
| | Often True | 0.273 | 0.016 | 17.098 | 0.000 | | 757 | |
| | Always True | 0.011 | 0.007 | 1.637 | 0.102 | | 4047 | |

*Abbreviations.* CIDI, Composite International Diagnostic Inventory definition of depression; Sex(M), Male; Est, estimate; SE, standard error; T, T-Value; P, P-Value; AdjR2, adjusted $R^2$ value; n, sample size.

© 2022 Chuong M et al. *JAMA Psychiatry.*

**eTable 10.** Adult Trauma Exposure and Broad Depression Regression Model Associations

| Trauma | Predictor | Est | SE | T | P | AdjR2 | Ncase | Ncontrol |
|---|---|---|---|---|---|---|---|---|
| *Been in a confiding relationship as an adult f.20522* | Intercept | 0.497 | 0.010 | 51.306 | 0.000 | 0.039 | | |
| | Sex(M) | -0.161 | 0.003 | -61.764 | 0.000 | | | |
| | Age | -0.002 | 0.000 | -10.886 | 0.000 | | | |
| | Rarely True | 0.042 | 0.004 | 11.866 | 0.000 | | 23516 | |
| | Sometimes True | 0.105 | 0.004 | 29.784 | 0.000 | | 23832 | 68306 |
| | Often True | 0.165 | 0.006 | 26.400 | 0.000 | | 6173 | |
| | Always True | 0.034 | 0.005 | 6.994 | 0.000 | | 11188 | |
| *Physical violence by partner or ex-partner as an adult f.20523.0.0* | Intercept | 0.483 | 0.010 | 50.427 | 0.000 | 0.044 | | |
| | Sex(M) | -0.144 | 0.003 | -55.719 | 0.000 | | | |
| | Age | -0.001 | 0.000 | -8.561 | 0.000 | | | |
| | Rarely True | 0.116 | 0.005 | 22.003 | 0.000 | | 8489 | |
| | Sometimes True | 0.190 | 0.006 | 30.850 | 0.000 | | 6151 | 119238 |
| | Often True | 0.278 | 0.015 | 18.971 | 0.000 | | 1038 | |
| | Always True | 0.315 | 0.014 | 23.250 | 0.000 | | 1219 | |
| *Belittlemen t by partner or ex-partner as an adult f.20521.0.0* | Intercept | 0.428 | 0.010 | 44.934 | 0.000 | 0.064 | | |
| | Sex(M) | -0.127 | 0.003 | -48.942 | 0.000 | | | |
| | Age | -0.001 | 0.000 | -6.229 | 0.000 | | | |
| | Rarely True | 0.109 | 0.004 | 25.140 | 0.000 | | 12900 | |
| | Sometimes True | 0.207 | 0.004 | 47.617 | 0.000 | | 13171 | 104338 |
| | Often True | 0.319 | 0.009 | 36.916 | 0.000 | | 3005 | |
| | Always True | 0.370 | 0.009 | 40.606 | 0.000 | | 2705 | |
| *Sexual interference by partner or ex-partner without consent as an adult f.20524.0.0* | Intercept | 0.508 | 0.010 | 53.160 | 0.000 | 0.038 | | |
| | Sex(M) | -0.144 | 0.003 | -55.107 | 0.000 | | | |
| | Age | -0.002 | 0.000 | -10.171 | 0.000 | | | |
| | Rarely True | 0.150 | 0.007 | 20.047 | 0.000 | | 4171 | |
| | Sometimes True | 0.215 | 0.010 | 22.449 | 0.000 | | 2483 | 128661 |
| | Often True | 0.313 | 0.024 | 13.003 | 0.000 | | 384 | |
| | Always True | 0.353 | 0.023 | 15.126 | 0.000 | | 409 | |
| *Able to pay rent/mortg age as an adult f.20525.0.0* | Intercept | 0.519 | 0.010 | 54.064 | 0.000 | 0.037 | | |
| | Sex(M) | -0.155 | 0.003 | -59.628 | 0.000 | | | |
| | Age | -0.002 | 0.000 | -11.155 | 0.000 | | | |
| | Rarely True | 0.080 | 0.005 | 16.240 | 0.000 | | 10042 | |
| | Sometimes True | 0.226 | 0.008 | 26.788 | 0.000 | | 3195 | 115814 |
| | Often True | 0.239 | 0.016 | 14.807 | 0.000 | | 854 | |
| | Always True | 0.038 | 0.007 | 5.296 | 0.000 | | 4581 | |

*Abbreviations.* Sex(M), Male; Est, estimate; SE, standard error; T, T-Value; P, P-Value; AdjR2, adjusted $R^2$ value; n, sample size.

© 2022 Chuong M et al. *JAMA Psychiatry.*

**eTable 11.** Adult Trauma Exposure and Neuroticism Regression Model Associations

| Trauma | Predictor | Est | SE | T | P | AdjR2 | Ncase | Ncontrol |
|---|---|---|---|---|---|---|---|---|
| *Been in a confiding relationship as an adult f.20522* | Intercept | 6.349 | 0.066 | 95.820 | 0.000 | 0.048 | | |
| | Sex(M) | -0.808 | 0.018 | -45.172 | 0.000 | | | |
| | Age | -0.043 | 0.001 | -37.495 | 0.000 | | | |
| | Rarely True | 0.267 | 0.024 | 10.930 | 0.000 | | 21360 | |
| | Sometimes True | 0.877 | 0.024 | 35.873 | 0.000 | | 21288 | 63266 |
| | Often True | 1.542 | 0.043 | 35.486 | 0.000 | | 5487 | |
| | Always True | 0.662 | 0.033 | 19.849 | 0.000 | | 9936 | |
| *Physical violence by partner or ex-partner as an adult f.20523.0.0* | Intercept | 6.484 | 0.066 | 98.057 | 0.000 | 0.034 | | |
| | Sex(M) | -0.749 | 0.018 | -41.605 | 0.000 | | | |
| | Age | -0.042 | 0.001 | -36.159 | 0.000 | | | |
| | Rarely True | 0.249 | 0.037 | 6.784 | 0.000 | | 7700 | |
| | Sometimes True | 0.757 | 0.042 | 17.870 | 0.000 | | 5741 | 108165 |
| | Often True | 0.931 | 0.100 | 9.274 | 0.000 | | 970 | |
| | Always True | 1.393 | 0.091 | 15.376 | 0.000 | | 1198 | |
| *Belittlement by partner or ex-partner as an adult f.20521.0.0* | Intercept | 6.045 | 0.066 | 91.735 | 0.000 | 0.054 | | |
| | Sex(M) | -0.628 | 0.018 | -34.987 | 0.000 | | | |
| | Age | -0.038 | 0.001 | -33.456 | 0.000 | | | |
| | Rarely True | 0.721 | 0.030 | 23.680 | 0.000 | | 11535 | |
| | Sometimes True | 1.240 | 0.030 | 41.386 | 0.000 | | 12150 | 94691 |
| | Often True | 1.543 | 0.059 | 26.116 | 0.000 | | 2822 | |
| | Always True | 1.806 | 0.062 | 29.261 | 0.000 | | 2590 | |
| *Sexual interference by partner or ex-partner without consent as an adult f.20524.0.0* | Intercept | 6.550 | 0.066 | 99.450 | 0.000 | 0.033 | | |
| | Sex(M) | -0.740 | 0.018 | -40.757 | 0.000 | | | |
| | Age | -0.043 | 0.001 | -36.875 | 0.000 | | | |
| | Rarely True | 0.555 | 0.052 | 10.588 | 0.000 | | 3708 | |
| | Sometimes True | 1.023 | 0.066 | 15.540 | 0.000 | | 2307 | 116944 |
| | Often True | 1.368 | 0.162 | 8.464 | 0.000 | | 372 | |
| | Always True | 1.493 | 0.153 | 9.764 | 0.000 | | 416 | |
| *Able to pay rent/mortgage as an adult f.20525.0.0* | Intercept | 6.573 | 0.066 | 99.695 | 0.000 | 0.036 | | |
| | Sex(M) | -0.778 | 0.018 | -43.366 | 0.000 | | | |
| | Age | -0.043 | 0.001 | -37.496 | 0.000 | | | |
| | Rarely True | 0.472 | 0.034 | 13.830 | 0.000 | | 9011 | |
| | Sometimes True | 1.164 | 0.058 | 20.059 | 0.000 | | 2950 | 105706 |
| | Often True | 1.522 | 0.109 | 13.937 | 0.000 | | 815 | |
| | Always True | 0.390 | 0.050 | 7.866 | 0.000 | | 4087 | |

*Abbreviations.* Sex(M), Male; Est, estimate; SE, standard error; T, T-Value; P, P-Value; AdjR2, adjusted $R^2$ value; n, sample size.

© 2022 Chuong M et al. *JAMA Psychiatry.*

**eTable 12.** Catastrophic Trauma Exposure and CIDI Depression Regression Model Associations

| Trauma | Predictor | Est | SE | T | P | AdjR2 | Ncase | Ncontrol |
|---|---|---|---|---|---|---|---|---|
| *Victim of sexual assault f.20531.0.0* | Intercept | 0.692 | 0.009 | 74.197 | 0.000 | 0.066 | | |
| | Sex(M) | -0.130 | 0.003 | -50.529 | 0.000 | | | |
| | Age | -0.007 | 0.000 | -41.146 | 0.000 | | | |
| | Yes, nw12 mths | 0.183 | 0.004 | 50.125 | 0.000 | | 17222 | 103230 |
| | Yes, w 12 mths | 0.193 | 0.040 | 4.848 | 0.000 | | 119 | |
| *Victim of physically violent crime f.20529.0.0* | Intercept | 0.719 | 0.009 | 76.182 | 0.000 | 0.051 | | |
| | Sex(M) | -0.161 | 0.003 | -62.895 | 0.000 | | | |
| | Age | -0.007 | 0.000 | -40.652 | 0.000 | | | |
| | Yes, nw12 mths | 0.075 | 0.003 | 22.860 | 0.000 | | 22490 | 98642 |
| | Yes, w 12 mths | 0.102 | 0.019 | 5.429 | 0.000 | | 546 | |
| *Been in serious accident believed to be life-threatening f.20526.0.0* | Intercept | 0.742 | 0.009 | 79.672 | 0.000 | 0.051 | | |
| | Sex(M) | -0.159 | 0.003 | -62.494 | 0.000 | | | |
| | Age | -0.007 | 0.000 | -43.033 | 0.000 | | | |
| | Yes, nw12 mths | 0.100 | 0.004 | 22.964 | 0.000 | | 11447 | 109967 |
| | Yes, w 12 mths | 0.098 | 0.024 | 4.142 | 0.000 | | 342 | |
| *Witnessed sudden violent death f.20530.0.0* | Intercept | 0.743 | 0.009 | 79.726 | 0.000 | 0.051 | | |
| | Sex(M) | -0.162 | 0.003 | -63.035 | 0.000 | | | |
| | Age | -0.007 | 0.000 | -43.197 | 0.000 | | | |
| | Yes, nw12 mths | 0.084 | 0.004 | 22.416 | 0.000 | | 16076 | 104955 |
| | Yes, w 12 mths | 0.064 | 0.017 | 3.691 | 0.000 | | 655 | |
| *Diagnosed with life-threatening illness f.20528.0.0* | Intercept | 0.770 | 0.009 | 82.523 | 0.000 | 0.050 | | |
| | Sex(M) | -0.155 | 0.003 | -60.841 | 0.000 | | | |
| | Age | -0.008 | 0.000 | -46.119 | 0.000 | | | |
| | Yes, nw12 mths | 0.075 | 0.004 | 20.386 | 0.000 | | 16877 | 102040 |
| | Yes, w 12 mths | 0.066 | 0.009 | 7.504 | 0.000 | | 2541 | |
| *Been involved in combat or exposed to war-zone f.20527.0.0* | Intercept | 0.757 | 0.009 | 81.216 | 0.000 | 0.047 | | |
| | Sex(M) | -0.155 | 0.003 | -60.441 | 0.000 | | | |
| | Age | -0.007 | 0.000 | -43.838 | 0.000 | | | |
| | Yes, nw12 mths | 0.036 | 0.007 | 5.272 | 0.000 | | 4298 | 117377 |
| | Yes, w 12 mths | 0.029 | 0.047 | 0.613 | 0.540 | | 89 | |

*Abbreviations.* CIDI, Composite International Diagnostic Inventory definition of depression; Sex(M), Male; Est, estimate; SE, standard error; T, T-Value; P, P-Value; AdjR2, adjusted $R^2$ value; n, sample size.

© 2022 Chuong M et al. *JAMA Psychiatry.*

**eTable 13.** Catastrophic Trauma Exposure and Broad Depression Regression Model Associations

| Trauma | Predictor | Est | SE | T | P | AdjR2 | Ncase | Ncontrol |
|---|---|---|---|---|---|---|---|---|
| *Victim of sexual assault f.20531.0.0* | Intercept | 0.486 | 0.010 | 50.520 | 0.000 | 0.041 | | |
| | Sex(M) | -0.142 | 0.003 | -54.083 | 0.000 | | | |
| | Age | -0.002 | 0.000 | -9.193 | 0.000 | | | |
| | Yes, nw12 mths | 0.149 | 0.004 | 40.390 | 0.000 | | 19575 | 115219 |
| | Yes, w 12 mths | 0.202 | 0.041 | 4.936 | 0.000 | | 132 | |
| *Victim of physically violent crime f.20529.0.0* | Intercept | 0.509 | 0.010 | 52.497 | 0.000 | 0.032 | | |
| | Sex(M) | -0.167 | 0.003 | -64.180 | 0.000 | | | |
| | Age | -0.002 | 0.000 | -9.154 | 0.000 | | | |
| | Yes, nw12 mths | 0.061 | 0.003 | 18.161 | 0.000 | | 19575 | 110680 |
| | Yes, w 12 mths | 0.097 | 0.019 | 5.116 | 0.000 | | 623 | |
| *Been in serious accident believed to be life-threatening f.20526.0.0* | Intercept | 0.529 | 0.010 | 55.287 | 0.000 | 0.031 | | |
| | Sex(M) | -0.166 | 0.003 | -63.759 | 0.000 | | | |
| | Age | -0.002 | 0.000 | -10.954 | 0.000 | | | |
| | Yes, nw12 mths | 0.074 | 0.004 | 16.711 | 0.000 | | 12638 | 123323 |
| | Yes, w 12 mths | 0.095 | 0.024 | 3.941 | 0.000 | | 385 | |
| *Witnessed sudden violent death f.20530.0.0* | Intercept | 0.533 | 0.010 | 55.632 | 0.000 | 0.030 | | |
| | Sex(M) | -0.165 | 0.003 | -63.096 | 0.000 | | | |
| | Age | -0.002 | 0.000 | -11.215 | 0.000 | | | |
| | Yes, nw12 mths | 0.041 | 0.004 | 10.621 | 0.000 | | 17559 | 118005 |
| | Yes, w 12 mths | 0.037 | 0.018 | 2.098 | 0.036 | | 724 | |
| *Diagnosed with life-threatening illness f.20528.0.0* | Intercept | 0.550 | 0.010 | 57.394 | 0.000 | 0.031 | | |
| | Sex(M) | -0.162 | 0.003 | -62.640 | 0.000 | | | |
| | Age | -0.002 | 0.000 | -13.466 | 0.000 | | | |
| | Yes, nw12 mths | 0.055 | 0.004 | 14.791 | 0.000 | | 19051 | 114029 |
| | Yes, w 12 mths | 0.068 | 0.009 | 7.628 | 0.000 | | 2898 | |
| *Been involved in combat or exposed to war-zone f.20527.0.0* | Intercept | 0.539 | 0.010 | 56.338 | 0.000 | 0.029 | | |
| | Sex(M) | -0.161 | 0.003 | -61.726 | 0.000 | | | |
| | Age | -0.002 | 0.000 | -11.408 | 0.000 | | | |
| | Yes, nw12 mths | -0.005 | 0.007 | -0.744 | 0.457 | | 4642 | 131632 |
| | Yes, w 12 mths | -0.015 | 0.049 | -0.299 | 0.765 | | 93 | |

*Abbreviations.* Sex(M), Male; Est, estimate; SE, standard error; T, T-Value; P, P-Value; AdjR2, adjusted $R^2$ value; n, sample size.

© 2022 Chuong M et al. *JAMA Psychiatry.*

**eTable 14.** Catastrophic Trauma Exposure and Neuroticism Regression Model Associations

| Trauma | Predictor | Est | SE | T | P | AdjR2 | Ncase | Ncontrol |
|---|---|---|---|---|---|---|---|---|
| *Victim of sexual assault f.20531.0.0* | Intercept | 6.455 | 0.066 | 97.406 | 0.000 | 0.034 | | |
| | Sex(M) | -0.724 | 0.018 | -39.870 | 0.000 | | | |
| | Age | -0.042 | 0.001 | -36.288 | 0.000 | | | |
| | Yes, nw12 mths | 0.643 | 0.025 | 25.228 | 0.000 | | 18064 | 104622 |
| | Yes, w 12 mths | 0.779 | 0.286 | 2.723 | 0.006 | | 118 | |
| *Victim of physically violent crime f.20529.0.0* | Intercept | 6.519 | 0.067 | 97.857 | 0.000 | 0.031 | | |
| | Sex(M) | -0.848 | 0.018 | -47.031 | 0.000 | | | |
| | Age | -0.041 | 0.001 | -35.609 | 0.000 | | | |
| | Yes, nw12 mths | 0.327 | 0.023 | 14.081 | 0.000 | | 22874 | 100423 |
| | Yes, w 12 mths | 0.492 | 0.131 | 3.764 | 0.000 | | 572 | |
| *Been in serious accident believed to be life-threatening f.20526.0.0* | Intercept | 6.649 | 0.066 | 101.066 | 0.000 | 0.030 | | |
| | Sex(M) | -0.828 | 0.018 | -46.035 | 0.000 | | | |
| | Age | -0.043 | 0.001 | -37.381 | 0.000 | | | |
| | Yes, nw12 mths | 0.234 | 0.030 | 7.665 | 0.000 | | 11692 | 111898 |
| | Yes, w 12 mths | 0.364 | 0.167 | 2.177 | 0.030 | | 349 | |
| *Witnessed sudden violent death f.20530.0.0* | Intercept | 6.688 | 0.066 | 101.595 | 0.000 | 0.029 | | |
| | Sex(M) | -0.809 | 0.018 | -44.778 | 0.000 | | | |
| | Age | -0.044 | 0.001 | -37.690 | 0.000 | | | |
| | Yes, nw12 mths | -0.021 | 0.027 | -0.790 | 0.430 | | 16292 | 106902 |
| | Yes, w 12 mths | -0.054 | 0.120 | -0.449 | 0.653 | | 682 | |
| *Diagnosed with life-threatening illness f.20528.0.0* | Intercept | 6.731 | 0.066 | 102.202 | 0.000 | 0.030 | | |
| | Sex(M) | -0.818 | 0.018 | -45.719 | 0.000 | | | |
| | Age | -0.045 | 0.001 | -38.710 | 0.000 | | | |
| | Yes, nw12 mths | 0.246 | 0.026 | 9.543 | 0.000 | | 17347 | 103619 |
| | Yes, w 12 mths | 0.369 | 0.061 | 6.057 | 0.000 | | 2699 | |
| *Been involved in combat or exposed to war-zone f.20527.0.0* | Intercept | 6.684 | 0.066 | 101.769 | 0.000 | 0.030 | | |
| | Sex(M) | -0.804 | 0.018 | -44.646 | 0.000 | | | |
| | Age | -0.043 | 0.001 | -37.617 | 0.000 | | | |
| | Yes, nw12 mths | -0.171 | 0.049 | -3.505 | 0.000 | | 4280 | 119584 |
| | Yes, w 12 mths | -0.496 | 0.342 | -1.450 | 0.147 | | 83 | |

*Abbreviations.* Sex(M), Male; w/nw, within/not within; Est, estimate; SE, standard error; T, T-Value; P, P-Value; AdjR2, adjusted $R^2$ value; n, sample size.

© 2022 Chuong M et al. *JAMA Psychiatry.*

**eTables 15-18** present associations between UK Biobank trauma exposure eigenvectors, or also known as principal components (PCs) and the phenotypes of interest; broad depression, CIDI depression and neuroticism. Trauma PCs were obtained for full, childhood, adult and catastrophic trauma separately. Full trauma PCs pre-corrected for the full sample GRMs were also explored. eTables 15-18 were presented in separate sections for each trauma exposure sub-category; full, childhood, adult and catastrophic trauma exposure. Tables include β coefficient estimates of associations, standard errors, T and P-Values as well as phenotypic variance accounted for by each model.

© 2022 Chuong M et al. *JAMA Psychiatry.*

**eTable 15.** Childhood Trauma Exposure Principal Components and Depression/Neuroticism Regression Model Associations

| | CIDI DEPRESSION (N = 128,927) | | | | | BROAD DEPRESSION (N = 486,165) | | | | | NEUROTICISM (N = 401,663) | | | | |
|---|---|---|---|---|---|---|---|---|---|---|---|---|---|---|---|
| Predictor | Est | SE | T | P | Cumulative Adjusted R2* | Est | SE | T | P | Cumulative Adjusted R2* | Est | SE | T | P | Cumulative Adjusted R2* |
| (Intercept) | 0.698 | 0.009 | 75.527 | 0.000 | NA | 0.497 | 0.010 | 51.947 | 0.000 | NA | 6.482 | 0.065 | 99.144 | 0.000 | NA |
| Age | -0.006 | 0.000 | -37.980 | 0.0 | 0.019 | -0.001 | 0.000 | -7.541 | 0.000 | 0.002 | -0.040 | 0.001 | -35.218 | 0.000 | 0.011 |
| Sex(M) | -0.141 | 0.003 | -56.103 | 0.000 | 0.047 | -0.150 | 0.003 | -57.814 | 0.000 | 0.028 | -0.751 | 0.018 | -42.218 | 0.000 | 0.032 |
| PC1 | 0.070 | 0.001 | 79.004 | 0.000 | 0.094 | 0.059 | 0.001 | 64.670 | 0.000 | 0.059 | 0.398 | 0.006 | 64.468 | 0.000 | 0.062 |
| PC2 | 0.019 | 0.001 | 13.862 | 0.000 | 0.095 | 0.011 | 0.001 | 8.181 | 0.000 | 0.059 | -0.031 | 0.010 | -3.285 | 0.001 | 0.062 |
| PC3 | 0.014 | 0.001 | 9.959 | 0.000 | 0.096 | 0.009 | 0.001 | 6.384 | 0.000 | 0.060 | 0.066 | 0.010 | 6.869 | 0.000 | 0.062 |
| PC4 | -0.035 | 0.002 | -21.690 | 0.000 | 0.100 | -0.037 | 0.002 | -22.318 | 0.000 | 0.063 | -0.351 | 0.011 | -31.272 | 0.000 | 0.070 |
| PC5 | 0.011 | 0.002 | 6.188 | 0.000 | 0.100 | 0.007 | 0.002 | 4.066 | 0.000 | 0.063 | 0.004 | 0.012 | 0.315 | 0.753 | 0.070 |

*Abbreviations.* CIDI, Composite International Diagnostic Inventory definition of depression; PC, principal component; Sex(M), Male; Est, estimate; SE, standard error; T, T-Value; P, P-Value; AdjR2; N, sample size. Coefficients are obtained from the full multiple regression model (including all predictors). *Represent adjusted R2 values when the specific predictor is ADDED to the model, on top of prior predictors.

**eTable 16.** Adult Trauma Exposure Principal Components and Depression/Neuroticism Regression Model Associations

| | CIDI DEPRESSION (N = 128,927) | | | | | BROAD DEPRESSION (N = 486,165) | | | | | NEUROTICISM (N = 401,663) | | | | |
|---|---|---|---|---|---|---|---|---|---|---|---|---|---|---|---|
| Predictor | Est | SE | T | P | Cumulative Adjusted R2* | Est | SE | T | P | Cumulative Adjusted R2* | Est | SE | T | P | Cumulative Adjusted R2* |
| (Intercept) | 0.683 | 0.009 | 73.522 | 0.000 | NA | 0.476 | 0.010 | 49.578 | 0.000 | NA | 6.411 | 0.066 | 97.180 | 0.000 | NA |
| Age | -0.006 | 0.000 | -37.358 | 0.000 | 0.019 | -0.001 | 0.000 | -6.454 | 0.000 | 0.002 | -0.040 | 0.001 | -34.593 | 0.000 | 0.011 |
| Sex(M) | -0.112 | 0.003 | -43.518 | 0.000 | 0.047 | -0.124 | 0.003 | -46.860 | 0.000 | 0.028 | -0.629 | 0.018 | -34.520 | 0.000 | 0.032 |
| PC1 | 0.071 | 0.001 | 77.007 | 0.000 | 0.092 | 0.062 | 0.001 | 65.473 | 0.000 | 0.060 | 0.309 | 0.006 | 48.234 | 0.000 | 0.047 |
| PC2 | 0.006 | 0.001 | 5.224 | 0.000 | 0.093 | 0.010 | 0.001 | 8.357 | 0.000 | 0.060 | 0.211 | 0.009 | 24.395 | 0.000 | 0.052 |
| PC3 | -0.011 | 0.001 | -7.885 | 0.000 | 0.093 | -0.010 | 0.001 | -6.892 | 0.000 | 0.061 | -0.179 | 0.010 | -17.947 | 0.000 | 0.055 |
| PC4 | -0.039 | 0.002 | -23.199 | 0.000 | 0.097 | -0.035 | 0.002 | -20.764 | 0.000 | 0.064 | -0.196 | 0.012 | -16.997 | 0.000 | 0.057 |
| PC5 | 0.061 | 0.002 | 31.521 | 0.000 | 0.105 | 0.044 | 0.002 | 22.353 | 0.000 | 0.067 | 0.373 | 0.013 | 27.948 | 0.000 | 0.063 |

*Abbreviations.* CIDI, Composite International Diagnostic Inventory definition of depression; PC, principal component; Sex(M), Male; Est, estimate; SE, standard error; T, T-Value; P, P-Value; N, sample size. Coefficients are obtained from the full multiple regression model (including all predictors). *Represent adjusted R2 values when the specific predictor is ADDED to the model, on top of prior predictors.

© 2022 Chuong M et al. *JAMA Psychiatry.*

**eTable 17.** Catastrophic Trauma Exposure Principal Components and Depression/Neuroticism Regression Model Associations

| | CIDI DEPRESSION (N = 128,927) | | | | | BROAD DEPRESSION (N = 486,165) | | | | | NEUROTICISM (N = 401,663) | | | | |
|---|---|---|---|---|---|---|---|---|---|---|---|---|---|---|---|
| **Predictor** | **Est** | **SE** | **T** | **P** | **Cumulative Adjusted R2*** | **Est** | **SE** | **T** | **P** | **Cumulative Adjusted R2*** | **Est** | **SE** | **T** | **P** | **Cumulative Adjusted R2*** |
| **(Intercept)** | 0.724 | 0.009 | 76.915 | 0.000 | NA | 0.509 | 0.010 | 52.401 | 0.000 | NA | 6.540 | 0.067 | 97.767 | 0.000 | NA |
| **Age** | -0.007 | 0.000 | -39.975 | 0.000 | 0.019 | -0.001 | 0.000 | -8.639 | 0.000 | 0.002 | -0.042 | 0.001 | -35.370 | 0.000 | 0.011 |
| **Sex(M)** | -0.148 | 0.003 | -56.024 | 0.000 | 0.047 | -0.152 | 0.003 | -56.271 | 0.000 | 0.028 | -0.753 | 0.019 | -40.154 | 0.000 | 0.032 |
| **PC1** | 0.046 | 0.001 | 42.547 | 0.000 | 0.062 | 0.031 | 0.001 | 28.004 | 0.000 | 0.035 | 0.101 | 0.008 | 13.284 | 0.000 | 0.031 |
| **PC2** | 0.043 | 0.001 | 33.730 | 0.000 | 0.070 | 0.041 | 0.001 | 31.211 | 0.000 | 0.042 | 0.214 | 0.009 | 23.734 | 0.000 | 0.035 |
| **PC3** | -0.022 | 0.001 | -17.182 | 0.000 | 0.072 | -0.020 | 0.001 | -15.156 | 0.000 | 0.044 | -0.106 | 0.009 | -11.697 | 0.000 | 0.036 |
| **PC4** | 0.016 | 0.001 | 11.761 | 0.000 | 0.073 | 0.010 | 0.001 | 7.416 | 0.000 | 0.044 | 0.031 | 0.009 | 3.312 | 0.001 | 0.036 |
| **PC5** | -0.014 | 0.001 | -10.466 | 0.000 | 0.074 | -0.011 | 0.001 | -7.926 | 0.000 | 0.045 | -0.010 | 0.010 | -1.041 | 0.298 | 0.036 |
| **PC6** | -0.010 | 0.001 | -7.143 | 0.000 | 0.075 | -0.004 | 0.001 | -3.043 | 0.002 | 0.045 | 0.020 | 0.010 | 2.036 | 0.042 | 0.036 |

*Abbreviations.* CIDI, Composite International Diagnostic Inventory definition of depression; PC, principal component; Sex(M), Male; Est, estimate; SE, standard error; T, T-Value; P, P-Value; N, sample size. Coefficients are obtained from the full multiple regression model (including all predictors). *Represent adjusted R2 values when the specific predictor is ADDED to the model, on top of prior predictors.

© 2022 Chuong M et al. *JAMA Psychiatry.*

**eTable 18.** Full Trauma Exposure Principal Components and Depression/Neuroticism Regression Model Associations

| | CIDI DEPRESSION (N = 128,927) | | | | | BROAD DEPRESSION (N = 486,165) | | | | | NEUROTICISM (N = 401,663) | | | | |
|---|---|---|---|---|---|---|---|---|---|---|---|---|---|---|---|
| Predictor | Est | SE | T | P | Cumulative Adjusted R2* | Est | SE | T | P | Cumulative Adjusted R2* | Est | SE | T | P | Cumulative Adjusted R2* |
| (Intercept) | 0.649 | 0.009 | 69.005 | 0.000 | NA | 0.450 | 0.010 | 45.870 | 0.000 | NA | 6.282 | 0.067 | 93.516 | 0.000 | NA |
| Age | -0.005 | 0.000 | -33.160 | 0.000 | 0.019 | -0.001 | 0.000 | -3.932 | 0.000 | 0.002 | -0.038 | 0.001 | -32.367 | 0.000 | 0.011 |
| Sex(M) | -0.116 | 0.003 | -43.328 | 0.000 | 0.047 | -0.122 | 0.003 | -44.101 | 0.000 | 0.028 | -0.615 | 0.019 | -32.340 | 0.000 | 0.032 |
| PC1 | -0.075 | 0.001 | -99.268 | 0.000 | 0.122 | -0.064 | 0.001 | -81.872 | 0.000 | 0.077 | -0.366 | 0.005 | -69.159 | 0.000 | 0.067 |
| PC2 | -0.006 | 0.001 | -5.406 | 0.000 | 0.122 | -0.005 | 0.001 | -4.918 | 0.000 | 0.078 | 0.114 | 0.007 | 15.426 | 0.000 | 0.069 |
| PC3 | 0.007 | 0.001 | 6.455 | 0.000 | 0.122 | -0.003 | 0.001 | -3.032 | 0.002 | 0.078 | -0.126 | 0.008 | -16.291 | 0.000 | 0.071 |
| PC4 | -0.004 | 0.001 | -3.516 | 0.000 | 0.123 | -0.003 | 0.001 | -2.407 | 0.016 | 0.078 | -0.006 | 0.008 | -0.764 | 0.445 | 0.071 |
| PC5 | -0.015 | 0.001 | -12.609 | 0.000 | 0.124 | -0.013 | 0.001 | -10.234 | 0.000 | 0.078 | -0.107 | 0.009 | -12.415 | 0.000 | 0.072 |
| PC6 | 0.013 | 0.001 | 10.531 | 0.000 | 0.125 | 0.011 | 0.001 | 8.502 | 0.000 | 0.079 | 0.043 | 0.009 | 4.833 | 0.000 | 0.072 |
| PC7 | 0.008 | 0.001 | 6.203 | 0.000 | 0.125 | 0.012 | 0.001 | 9.377 | 0.000 | 0.079 | 0.099 | 0.009 | 10.873 | 0.000 | 0.073 |
| PC8 | -0.002 | 0.001 | -1.215 | 0.224 | 0.125 | 0.000 | 0.001 | -0.016 | 0.987 | 0.079 | 0.072 | 0.010 | 7.553 | 0.000 | 0.074 |
| PC9 | -0.010 | 0.001 | -7.643 | 0.000 | 0.125 | -0.012 | 0.001 | -8.400 | 0.000 | 0.080 | -0.194 | 0.010 | -19.849 | 0.000 | 0.077 |
| PC10 | -0.013 | 0.001 | -9.781 | 0.000 | 0.126 | -0.008 | 0.001 | -5.861 | 0.000 | 0.080 | -0.073 | 0.010 | -7.384 | 0.000 | 0.077 |
| PC11 | -0.014 | 0.002 | -9.478 | 0.000 | 0.127 | -0.012 | 0.002 | -7.912 | 0.000 | 0.081 | -0.086 | 0.011 | -8.015 | 0.000 | 0.078 |
| PC12 | 0.046 | 0.002 | 28.633 | 0.000 | 0.133 | 0.044 | 0.002 | 26.448 | 0.000 | 0.086 | 0.341 | 0.011 | 29.976 | 0.000 | 0.085 |
| PC13 | 0.011 | 0.002 | 6.475 | 0.000 | 0.133 | 0.009 | 0.002 | 5.535 | 0.000 | 0.086 | 0.009 | 0.011 | 0.787 | 0.431 | 0.085 |
| PC14 | 0.013 | 0.002 | 7.325 | 0.000 | 0.133 | 0.008 | 0.002 | 4.203 | 0.000 | 0.086 | 0.039 | 0.012 | 3.133 | 0.002 | 0.085 |
| PC15 | 0.018 | 0.002 | 10.150 | 0.000 | 0.134 | 0.015 | 0.002 | 8.228 | 0.000 | 0.086 | 0.043 | 0.013 | 3.384 | 0.001 | 0.085 |
| PC16 | 0.042 | 0.002 | 21.569 | 0.000 | 0.138 | 0.026 | 0.002 | 13.468 | 0.000 | 0.088 | 0.242 | 0.013 | 18.073 | 0.000 | 0.088 |

*Abbreviations.* CIDI, Composite International Diagnostic Inventory definition of depression; PC, principal component; Sex(M), Male; Est, estimate; SE, standard error; T, T-Value; P, P-Value; N, sample size. Coefficients are obtained from the full multiple regression model (including all predictors). *Represent adjusted R2 values when the specific predictor is ADDED to the model, on top of prior predictors.

© 2022 Chuong M et al. *JAMA Psychiatry.*

**eTables 19-22** present trauma exposure principal component (PC) loadings. Here, values represent how each trauma exposure question loads on to the separate PCs. Loadings are presented separately for each trauma exposure sub-category; full, childhood, adult and catastrophic trauma exposure. eTables 19-22 are presented in separate sections for each trauma exposure sub-category.

**eTable 19.** Childhood Trauma Exposure Principal Component Loadings

| Trauma | UKB Field | PC1 | PC2 | PC3 | PC4 | PC5 |
|---|---|---|---|---|---|---|
| **Childhood** | f.20489.0.0 | 0.511 | -0.228 | 0.080 | -0.683 | -0.463 |
| | f.20488.0.0 | 0.491 | 0.141 | 0.383 | 0.648 | -0.416 |
| | f.20487.0.0 | 0.528 | 0.070 | 0.328 | -0.113 | 0.772 |
| | f.20490.0.0 | 0.310 | 0.734 | -0.600 | -0.062 | -0.033 |
| | f.20491.0.0 | 0.351 | -0.619 | -0.616 | 0.313 | 0.124 |

*Abbreviations.* UKB, UK Biobank; PC, principal component.

**eTable 20.** Adult Trauma Exposure Principal Component Loadings

| Trauma | UKB Field | PC1 | PC2 | PC3 | PC4 | PC5 |
|---|---|---|---|---|---|---|
| **Adult** | f.20522.0.0 | 0.168 | 0.701 | -0.691 | 0.029 | -0.047 |
| | f.20523.0.0 | 0.578 | -0.155 | 0.022 | -0.298 | -0.743 |
| | f.20521.0.0 | 0.567 | -0.142 | -0.071 | -0.471 | 0.657 |
| | f.20524.0.0 | 0.525 | -0.157 | -0.004 | 0.830 | 0.107 |
| | f.20525.0.0 | 0.203 | 0.663 | 0.719 | -0.004 | 0.042 |

*Abbreviations.* UKB, UK Biobank; PC, principal component.

**eTable 21.** Catastrophic Trauma Exposure Principal Component Loadings

| Trauma | UKB Field | PC1 | PC2 | PC3 | PC4 | PC5 | PC6 |
|---|---|---|---|---|---|---|---|
| **Catastrophic** | f.20531.0.0 | 0.263 | 0.723 | -0.122 | 0.545 | -0.310 | -0.015 |
| | f.20529.0.0 | 0.458 | 0.373 | 0.137 | -0.262 | 0.738 | 0.139 |
| | f.20526.0.0 | 0.479 | 0.039 | 0.033 | -0.600 | -0.584 | 0.260 |
| | f.20530.0.0 | 0.527 | -0.276 | 0.154 | 0.105 | -0.032 | -0.781 |
| | f.20528.0.0 | 0.179 | -0.135 | -0.965 | -0.054 | 0.124 | -0.034 |
| | f.20527.0.0 | 0.426 | -0.493 | 0.106 | 0.511 | 0.043 | 0.549 |

*Abbreviations.* UKB, UK Biobank; PC, principal component.

© 2022 Chuong M et al. *JAMA Psychiatry.*

**eTable 22.** Full Trauma Exposure Principal Component Loadings

| Trauma | UKB Field | PC1 | PC2 | PC3 | PC4 | PC5 | PC6 | PC7 | PC8 | PC9 | PC10 | PC11 | PC12 | PC13 | PC14 | PC15 | PC16 |
|---|---|---|---|---|---|---|---|---|---|---|---|---|---|---|---|---|---|
| Childhood | f.20489.0.0 | -0.341 | 0.340 | -0.086 | 0.057 | -0.224 | 0.008 | -0.014 | 0.016 | -0.148 | -0.057 | 0.138 | 0.570 | -0.232 | -0.514 | -0.120 | -0.073 |
| | f.20488.0.0 | -0.324 | 0.256 | 0.129 | 0.120 | -0.364 | 0.011 | -0.071 | -0.007 | 0.140 | 0.068 | -0.293 | -0.597 | 0.168 | -0.369 | 0.088 | 0.149 |
| | f.20487.0.0 | -0.363 | 0.239 | 0.047 | 0.136 | -0.380 | 0.035 | -0.044 | -0.029 | 0.038 | 0.031 | -0.150 | 0.124 | -0.160 | 0.742 | 0.030 | -0.157 |
| | f.20490.0.0 | -0.279 | -0.043 | 0.136 | 0.522 | 0.380 | -0.008 | -0.030 | -0.034 | -0.026 | -0.018 | -0.041 | 0.021 | 0.289 | 0.054 | -0.626 | 0.056 |
| | f.20491.0.0 | -0.226 | 0.375 | -0.261 | -0.073 | 0.221 | 0.008 | -0.010 | -0.102 | 0.207 | 0.069 | 0.724 | -0.216 | 0.171 | 0.113 | 0.123 | 0.023 |
| Adult | f.20522.0.0 | -0.163 | 0.249 | -0.285 | -0.221 | 0.256 | -0.106 | 0.123 | 0.238 | -0.654 | -0.314 | -0.222 | -0.213 | 0.046 | 0.092 | 0.019 | -0.023 |
| | f.20523.0.0 | -0.341 | -0.409 | -0.098 | -0.254 | -0.088 | 0.004 | -0.037 | -0.003 | 0.036 | 0.031 | 0.011 | -0.016 | 0.351 | -0.100 | -0.018 | -0.705 |
| | f.20521.0.0 | -0.356 | -0.362 | -0.104 | -0.220 | -0.141 | 0.019 | -0.040 | -0.005 | -0.064 | -0.013 | 0.016 | 0.270 | 0.364 | 0.086 | 0.138 | 0.651 |
| | f.20524.0.0 | -0.316 | -0.401 | -0.107 | -0.128 | 0.016 | 0.009 | -0.062 | -0.033 | -0.009 | -0.011 | 0.162 | -0.329 | -0.692 | -0.016 | -0.278 | 0.122 |
| | f.20525.0.0 | -0.161 | 0.176 | -0.313 | -0.272 | 0.422 | -0.020 | 0.062 | -0.091 | 0.500 | 0.191 | -0.506 | 0.154 | -0.110 | -0.026 | -0.015 | 0.033 |
| Catastrophic | f.20531.0.0 | -0.296 | -0.188 | 0.169 | 0.437 | 0.373 | -0.020 | 0.019 | 0.018 | -0.051 | -0.032 | -0.030 | 0.052 | -0.173 | -0.073 | 0.685 | -0.074 |
| | f.20529.0.0 | -0.125 | 0.016 | 0.368 | -0.150 | 0.011 | -0.247 | 0.538 | 0.631 | 0.176 | 0.163 | 0.118 | 0.022 | -0.021 | 0.020 | -0.065 | 0.027 |
| | f.20526.0.0 | -0.094 | 0.069 | 0.391 | -0.238 | 0.073 | -0.060 | 0.438 | -0.683 | -0.261 | 0.200 | -0.003 | -0.012 | -0.008 | -0.004 | -0.014 | 0.000 |
| | f.20530.0.0 | -0.080 | 0.092 | 0.458 | -0.286 | 0.119 | -0.087 | -0.248 | -0.073 | 0.271 | -0.727 | 0.020 | 0.038 | -0.008 | 0.014 | -0.014 | -0.002 |
| | f.20528.0.0 | -0.042 | 0.039 | 0.129 | -0.095 | 0.086 | 0.956 | 0.167 | 0.139 | -0.011 | -0.048 | -0.004 | -0.014 | -0.003 | -0.006 | -0.010 | -0.003 |
| | f.20527.0.0 | -0.049 | 0.126 | 0.364 | -0.271 | 0.204 | 0.006 | -0.628 | 0.177 | -0.236 | 0.501 | 0.009 | 0.032 | -0.022 | 0.019 | -0.002 | 0.001 |

*Abbreviations.* UKB, UK Biobank; PC, principal component.

© 2022 Chuong M et al. *JAMA Psychiatry.*

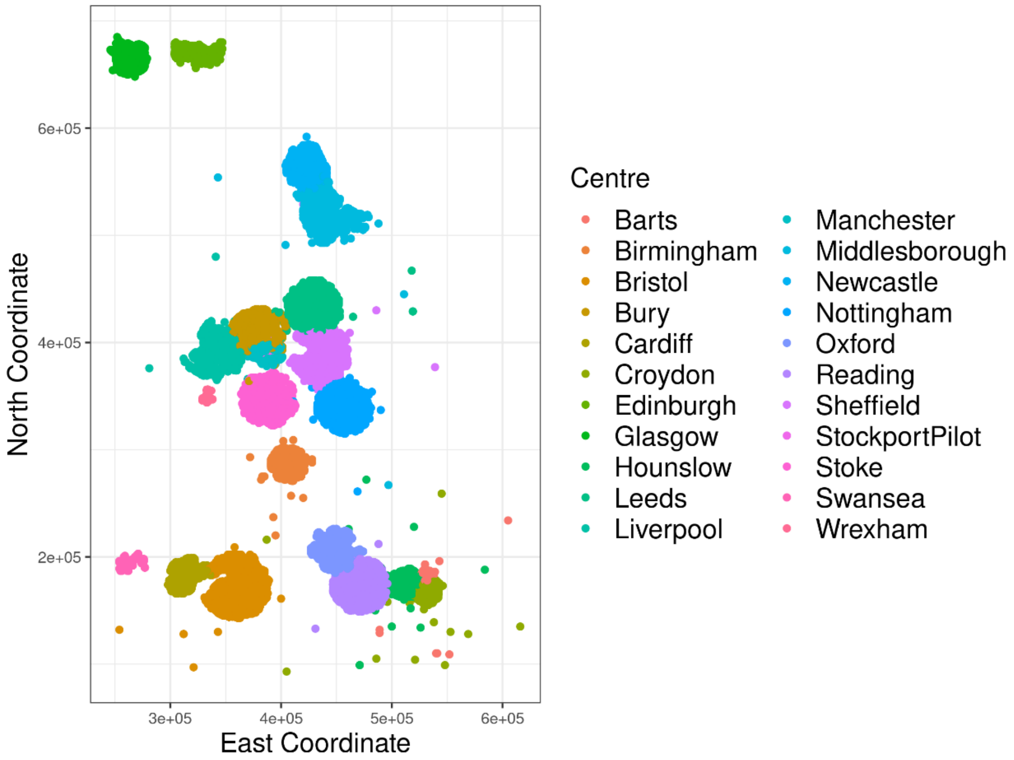


| Clusters | Centre | Sample Size |
|---|---|---|
| **North** | Glasgow | 4503 |
| | Edinburgh | 5592 |
| | Newcastle | 9771 |
| | Middlesborough | 6214 |
| **Mid North** | Leeds | 12332 |
| | Bury | 6915 |
| | Manchester | 3982 |
| | Liverpool | 8969 |
| **Mid South** | Wrexham | 151 |
| | Stoke | 4304 |
| | StockportPilot | 294 |
| | Sheffield | 9290 |
| | Nottingham | 10193 |
| | Birmingham | 6945 |
| **South West** | Swansea | 660 |
| | Cardiff | 4731 |
| | Bristol | 14642 |
| **South East** | Oxford | 4897 |
| | Reading | 10904 |
| | Hounslow | 9542 |
| | Croydon | 8860 |
| | Barts | 4438 |

© 2022 Chuong M et al. *JAMA Psychiatry.*

**eFigure 2.** UK Biobank Geographical Clusters. Above, figure shows the North and East co-ordinates of participant residence and colours represent the recruitment centres for each participant. Below, table shows geographical clusters, respective centres and sample sizes.

© 2022 Chuong M et al. *JAMA Psychiatry.*

**eTables 23-27** present demographic information on individuals within each geographical cluster; North, Mid-North, Mid-South, South-West, South-East. Demographic information for each cluster was presented when clusters were formed using all individuals responding to ALL trauma exposure questions. Additional demographic information tables were provided for only unrelated individuals responding to all trauma exposure questions as well as all individuals who have responded to all childhood, adult and catastrophic trauma exposure questions separately. eTables 23-27 were presented in separate sections for each sample.

© 2022 Chuong M et al. *JAMA Psychiatry.*

**eTable 23.** Cluster Demographics Using All Participants With Complete Trauma Exposure Responses

| DEMOGRPAHIC | NORTH CLUSTER | | | | | MIDNORTH CLUSTER | | | | | MIDSOUTH CLUSTER | | | | | SOUTHWEST CLUSTER | | | | | SOUTHEAST CLUSTER | | | | |
|---|---|---|---|---|---|---|---|---|---|---|---|---|---|---|---|---|---|---|---|---|---|---|---|---|---|
| Total Nind | 24287 | | | | | 29981 | | | | | 28961 | | | | | 18696 | | | | | 36323 | | | | |
| Sex | Female | Male | | | | Female | Male | | | | Female | Male | | | | Female | Male | | | | Female | Male | | | |
| | 13433 | 10854 | | | | 16595 | 13386 | | | | 15835 | 13126 | | | | 10533 | 8163 | | | | 20599 | 15724 | | | |
| Age | Min | Mean | Max | | | Min | Mean | Max | | | Min | Mean | Max | | | Min | Mean | Max | | | Min | Mean | Max | | |
| | 40 | 56.2 | 70.3 | | | 40.2 | 56.51 | 72.5 | | | 40.1 | 56.89 | 72.9 | | | 40.2 | 56.05 | 70.4 | | | 38.9 | 56.66 | 70.6 | | |
| | Case F | Case M | Con F | Con M | Total | Case F | Case M | Con F | Con M | Total | Case F | Case M | Con F | Con M | Total | Case F | Case M | Con F | Con M | Total | Case F | Case M | Con F | Con M | Total |
| CIDI Depression | 3726 | 1814 | 7053 | 7418 | 20011 | 4913 | 2277 | 8534 | 9054 | 24778 | 4876 | 2282 | 7644 | 8795 | 23597 | 3012 | 1371 | 5565 | 5623 | 15571 | 5402 | 2342 | 11050 | 11073 | 29867 |
| Broad Depression | 5225 | 2654 | 7053 | 7418 | 22350 | 6706 | 3546 | 8513 | 8897 | 27662 | 6563 | 3365 | 7892 | 8876 | 26696 | 3928 | 1989 | 5729 | 5634 | 17280 | 7530 | 3587 | 11253 | 11105 | 33475 |
| Neuroticism | Mean F | Total F | Mean M | Total M | | Mean F | Total F | Mean M | Total M | | Mean F | Total F | Mean M | Total M | | Mean F | Total F | Mean M | Total M | | Mean F | Total F | Mean M | Total M | |
| | 4.28 | 11375 | 3.41 | 9337 | | 4.24 | 13748 | 3.38 | 11438 | | 4.33 | 13158 | 3.36 | 11302 | | 4.20 | 8832 | 3.31 | 7019 | | 4.06 | 17141 | 3.32 | 13645 | |

*Abbreviations.* Nind, number of individuals; Min, minimum; Max, maximum; F, M, Female and Male, respectively.

**eTable 24.** Cluster Demographics Using Unrelated Participants With Complete Trauma Exposure Responses

| DEMOGRPAHIC | NORTH CLUSTER | | | | | MIDNORTH CLUSTER | | | | | MIDSOUTH CLUSTER | | | | | SOUTHWEST CLUSTER | | | | | SOUTHEAST CLUSTER | | | | |
|---|---|---|---|---|---|---|---|---|---|---|---|---|---|---|---|---|---|---|---|---|---|---|---|---|---|
| Total Nind | 20994 | | | | | 26566 | | | | | 25691 | | | | | 16841 | | | | | 33128 | | | | |
| Sex | Female | Male | | | | Female | Male | | | | Female | Male | | | | Female | Male | | | | Female | Male | | | |
| | 11626 | 9368 | | | | 14729 | 11837 | | | | 14048 | 11643 | | | | 9473 | 7368 | | | | 18735 | 14393 | | | |
| Age | Min | Mean | Max | | | Min | Mean | Max | | | Min | Mean | Max | | | Min | Mean | Max | | | Min | Mean | Max | | |
| | 40 | 56.14 | 70.3 | | | 40.3 | 56.47 | 72.5 | | | 40.1 | 56.86 | 72.9 | | | 40.2 | 56.03 | 70.4 | | | 40.2 | 56.60 | 70.6 | | |
| | Case F | Case M | Con F | Con M | Total | Case F | Case M | Con F | Con M | Total | Case F | Case M | Con F | Con M | Total | Case F | Case M | Con F | Con M | Total | Case F | Case M | Con F | Con M | Total |
| CIDI Depression | 3226 | 1575 | 6125 | 6461 | 17387 | 4395 | 2020 | 7552 | 8007 | 21974 | 4346 | 2065 | 6774 | 7775 | 20960 | 2710 | 1244 | 4988 | 5066 | 14008 | 4974 | 2131 | 10030 | 10165 | 27300 |
| Broad Depression | 4494 | 2287 | 6114 | 6395 | 19290 | 5960 | 3148 | 7538 | 7855 | 24501 | 5828 | 3002 | 6992 | 7858 | 23680 | 3551 | 1789 | 5122 | 5090 | 15552 | 6852 | 3253 | 10230 | 10195 | 30530 |
| Neuroticism | Mean F | Total F | Mean M | Total M | | Mean F | Total F | Mean M | Total M | | Mean F | Total F | Mean M | Total M | | Mean F | Total F | Mean M | Total M | | Mean F | Total F | Mean M | Total M | |
| | 4.25 | 9826 | 3.41 | 8074 | | 4.24 | 12213 | 3.38 | 10118 | | 4.32 | 11689 | 3.36 | 10019 | | 4.20 | 7940 | 3.31 | 6351 | | 4.06 | 15598 | 3.31 | 12481 | |

*Abbreviations.* Nind, number of individuals; Min, minimum; Max, maximum; F, M, Female and Male, respectively.

© 2022 Chuong M et al. *JAMA Psychiatry.*

**eTable 25.** Cluster Demographics Using All Participants With Complete Childhood Trauma Exposure Responses

| DEMOGRPAHIC | NORTH CLUSTER | | | | | MIDNORTH CLUSTER | | | | | MIDSOUTH CLUSTER | | | | | SOUTHWEST CLUSTER | | | | | SOUTHEAST CLUSTER | | | | |
|---|---|---|---|---|---|---|---|---|---|---|---|---|---|---|---|---|---|---|---|---|---|---|---|---|---|
| Total Nind | 25466 | | | | | 31458 | | | | | 30463 | | | | | 19603 | | | | | 37861 | | | | |
| Sex | Female | Male | | | | Female | Male | | | | Female | Male | | | | Female | Male | | | | Female | Male | | | |
| | 14197 | 11269 | | | | 17561 | 13897 | | | | 16824 | 13639 | | | | 11126 | 8477 | | | | 21662 | 16199 | | | |
| Age | Min | Mean | Max | | | Min | Mean | Max | | | Min | Mean | Max | | | Min | Mean | Max | | | Min | Mean | Max | | |
| | 40.00 | 56.25 | 70.30 | | | 40.20 | 56.56 | 72.50 | | | 40.10 | 56.95 | 72.90 | | | 40.20 | 56.10 | 70.40 | | | 38.90 | 56.71 | 70.60 | | |
| | Case F | Case M | Con F | Con M | Total | Case F | Case M | Con F | Con M | Total | Case F | Case M | Con F | Con M | Total | Case F | Case M | Con F | Con M | Total | Case F | Case M | Con F | Con M | Total |
| CIDI Depression | 3941 | 1864 | 7424 | 7754 | 20983 | 5178 | 2357 | 9007 | 9380 | 25922 | 5129 | 2375 | 8107 | 9117 | 24728 | 3159 | 1419 | 5859 | 5828 | 16265 | 5686 | 2424 | 11558 | 11371 | 31039 |
| Broad Depression | 5580 | 2759 | 7397 | 7691 | 23427 | 7147 | 3688 | 8963 | 9218 | 29016 | 6980 | 3515 | 8366 | 9204 | 28065 | 4168 | 2066 | 6026 | 5842 | 18102 | 7960 | 3720 | 11781 | 11406 | 34867 |
| Neuroticism | Mean F | Total F | Mean M | Total M | | Mean F | Total F | Mean M | Total M | | Mean F | Total F | Mean M | Total M | | Mean F | Total F | Mean M | Total M | | Mean F | Total F | Mean M | Total M | |
| | 4.30 | 11912 | 3.42 | 9653 | | 4.26 | 14440 | 3.40 | 11819 | | 4.35 | 13833 | 3.39 | 11675 | | 4.21 | 9255 | 3.32 | 7234 | | 4.08 | 17875 | 3.34 | 13974 | |

*Abbreviations.* Nind, number of individuals; Min, minimum; Max, maximum; F, M, Female and Male, respectively.

**eTable 26.** Cluster Demographics Using All Participants With Complete Adult Trauma Exposure Responses

| DEMOGRPAHIC | NORTH CLUSTER | | | | | MIDNORTH CLUSTER | | | | | MIDSOUTH CLUSTER | | | | | SOUTHWEST CLUSTER | | | | | SOUTHEAST CLUSTER | | | | |
|---|---|---|---|---|---|---|---|---|---|---|---|---|---|---|---|---|---|---|---|---|---|---|---|---|---|
| Total Nind | 25043 | | | | | 30914 | | | | | 29899 | | | | | 19246 | | | | | 37273 | | | | |
| Sex | Female | Male | | | | Female | Male | | | | Female | Male | | | | Female | Male | | | | Female | Male | | | |
| | 13939 | 11104 | | | | 17257 | 13657 | | | | 16504 | 13395 | | | | 10924 | 8322 | | | | 21254 | 16019 | | | |
| Age | Min | Mean | Max | | | Min | Mean | Max | | | Min | Mean | Max | | | Min | Mean | Max | | | Min | Mean | Max | | |
| | 40.00 | 56.20 | 70.30 | | | 40.20 | 56.51 | 72.50 | | | 40.10 | 56.92 | 72.90 | | | 40.20 | 56.09 | 70.40 | | | 38.90 | 56.68 | 70.60 | | |
| | Case F | Case M | Con F | Con M | Total | Case F | Case M | Con F | Con M | Total | Case F | Case M | Con F | Con M | Total | Case F | Case M | Con F | Con M | Total | Case F | Case M | Con F | Con M | Total |
| CIDI Depression | 3887 | 1866 | 7289 | 7612 | 20654 | 5145 | 2344 | 8808 | 9196 | 25493 | 5105 | 2345 | 7898 | 8934 | 24282 | 3141 | 1405 | 5740 | 5714 | 16000 | 5613 | 2411 | 11311 | 11220 | 30555 |
| Broad Depression | 5480 | 2740 | 7262 | 7549 | 23031 | 7031 | 3632 | 8777 | 9047 | 28487 | 6891 | 3457 | 8153 | 9024 | 27525 | 4091 | 2040 | 5920 | 5727 | 17778 | 7826 | 3698 | 11527 | 11248 | 34299 |
| Neuroticism | Mean F | Total F | Mean M | Total M | | Mean F | Total F | Mean M | Total M | | Mean F | Total F | Mean M | Total M | | Mean F | Total F | Mean M | Total M | | Mean F | Total F | Mean M | Total M | |
| | 4.30 | 11743 | 3.43 | 9522 | | 4.26 | 14225 | 3.41 | 11642 | | 4.35 | 13624 | 3.38 | 11500 | | 4.21 | 9117 | 3.33 | 7124 | | 4.08 | 17584 | 3.35 | 13833 | |

*Abbreviations.* Nind, number of individuals; Min, minimum; Max, maximum; F, M, Female and Male, respectively.

© 2022 Chuong M et al. *JAMA Psychiatry.*

**eTable 27.** Cluster Demographics Using All Participants With Catastrophic Trauma Exposure Responses

| DEMOGRPAHIC | NORTH CLUSTER | | | | |
|---|---|---|---|---|---|
| Total Nind | 25590 | | | | |
| Sex | Female | Male | | | |
| | 14262 | 11328 | | | |
| Age | Min | Mean | Max | | |
| | 40.00 | 56.27 | 70.30 | | |
| | Case F | Case M | Con F | Con M | Total |
| CIDI Depression | 3951 | 1881 | 7454 | 7792 | 21078 |
| Broad Depression | 5613 | 2776 | 7417 | 7729 | 23535 |
| Neuroticism | Mean F | Total F | Mean M | Total M | |
| | 4.29 | 11958 | 3.42 | 9704 | |

| DEMOGRPAHIC | MIDNORTH CLUSTER | | | | |
|---|---|---|---|---|---|
| Total Nind | 31563 | | | | |
| Sex | Female | Male | | | |
| | 17627 | 13936 | | | |
| Age | Min | Mean | Max | | |
| | 40.20 | 56.56 | 72.50 | | |
| | Case F | Case M | Con F | Con M | Total |
| CIDI Depression | 5208 | 2361 | 9012 | 9411 | 25992 |
| Broad Depression | 7206 | 3698 | 8967 | 9245 | 29116 |
| Neuroticism | Mean F | Total F | Mean M | Total M | |
| | 4.26 | 14494 | 3.40 | 11860 | |

| DEMOGRPAHIC | MIDSOUTH CLUSTER | | | | |
|---|---|---|---|---|---|
| Total Nind | 30543 | | | | |
| Sex | Female | Male | | | |
| | 16850 | 13693 | | | |
| Age | Min | Mean | Max | | |
| | 40.10 | 56.97 | 72.90 | | |
| | Case F | Case M | Con F | Con M | Total |
| CIDI Depression | 5166 | 2376 | 8115 | 9160 | 24817 |
| Broad Depression | 6990 | 3525 | 8377 | 9232 | 28124 |
| Neuroticism | Mean F | Total F | Mean M | Total M | |
| | 4.36 | 13854 | 3.38 | 11729 | |

| DEMOGRPAHIC | SOUTHWEST CLUSTER | | | | |
|---|---|---|---|---|---|
| Total Nind | 19694 | | | | |
| Sex | Female | Male | | | |
| | 11195 | 8499 | | | |
| Age | Min | Mean | Max | | |
| | 40.20 | 56.13 | 70.40 | | |
| | Case F | Case M | Con F | Con M | Total |
| CIDI Depression | 3176 | 1419 | 5898 | 5842 | 16335 |
| Broad Depression | 4201 | 2074 | 6062 | 5856 | 18193 |
| Neuroticism | Mean F | Total F | Mean M | Total M | |
| | 4.22 | 9298 | 3.31 | 7247 | |

| DEMOGRPAHIC | SOUTHEAST CLUSTER | | | | |
|---|---|---|---|---|---|
| Total Nind | 38009 | | | | |
| Sex | Female | Male | | | |
| | 21733 | 16276 | | | |
| Age | Min | Mean | Max | | |
| | 38.90 | 56.72 | 70.60 | | |
| | Case F | Case M | Con F | Con M | Total |
| CIDI Depression | 5692 | 2427 | 11593 | 11427 | 31139 |
| Broad Depression | 8023 | 3744 | 11794 | 11448 | 35009 |
| Neuroticism | Mean F | Total F | Mean M | Total M | |
| | 4.08 | 17926 | 3.35 | 14038 | |

*Abbreviations.* Nind, number of individuals; Min, minimum; Max, maximum; F, M, Female and Male, respectively.

© 2022 Chuong M et al. *JAMA Psychiatry.*

**eTables 28-31** present demographic information on individuals within each geographical cluster; North, Mid-North, Mid-South, South-West, South-East. Demographic information for each cluster was presented when clusters were formed using all individuals responding to ALL trauma exposure questions. eTables 28-31 were presented in separate sections for each sex.

**eTable 28.** Cluster Demographics Using All Female Participants With Complete Trauma Exposure Responses

| DEMOGRPAHIC | NORTH CLUSTER | | | MIDNORTH CLUSTER | | | MIDSOUTH CLUSTER | | | SOUTHWEST CLUSTER | | | SOUTHEAST CLUSTER | | |
|---|---|---|---|---|---|---|---|---|---|---|---|---|---|---|---|
| **Total Nind** | 13433 | | | 16595 | | | 15835 | | | 10533 | | | 20599 | | |
| **Age** | **Min** | **Mean** | **Max** | **Min** | **Mean** | **Max** | **Min** | **Mean** | **Max** | **Min** | **Mean** | **Max** | **Min** | **Mean** | **Max** |
| | 40.20 | 55.63 | 70.30 | 40.20 | 56.01 | 70.30 | 40.10 | 56.27 | 70.40 | 40.20 | 55.51 | 70.40 | 40.20 | 56.26 | 70.60 |
| | **Case** | **Control** | **Total** | **Case** | **Control** | **Total** | **Case** | **Control** | **Total** | **Case** | **Control** | **Total** | **Case** | **Control** | **Total** |
| **CIDI Depression** | 3726 | 7067 | 10793 | 4913 | 8534 | 13447 | 4876 | 7644 | 12520 | 3012 | 5565 | 8577 | 5402 | 11050 | 16452 |
| **Broad Depression** | 5225 | 7053 | 12278 | 6706 | 8513 | 15219 | 6563 | 7892 | 14455 | 3928 | 5729 | 9657 | 7530 | 11253 | 18783 |
| **Neuroticism** | **Mean** | **Total** | | **Mean** | **Total** | | **Mean** | **Total** | | **Mean** | **Total** | | **Mean** | **Total** | |
| | 4.28 | 11375 | | 4.24 | 13748 | | 4.33 | 13158 | | 4.20 | 8832 | | 4.06 | 17141 | |

*Abbreviations.* Nind, number of individuals; Min, minimum; Max, maximum.

**eTable 29.** Cluster Demographics Using Unrelated Female Participants With Complete Trauma Exposure Responses

| DEMOGRPAHIC | NORTH CLUSTER | | | MIDNORTH CLUSTER | | | MIDSOUTH CLUSTER | | | SOUTHWEST CLUSTER | | | SOUTHEAST CLUSTER | | |
|---|---|---|---|---|---|---|---|---|---|---|---|---|---|---|---|
| **Total Nind** | 11626 | | | 14729 | | | 14048 | | | 9473 | | | 18735 | | |
| **Age** | **Min** | **Mean** | **Max** | **Min** | **Mean** | **Max** | **Min** | **Mean** | **Max** | **Min** | **Mean** | **Max** | **Min** | **Mean** | **Max** |
| | 40.20 | 55.56 | 70.30 | 40.30 | 55.98 | 70.30 | 40.10 | 56.25 | 70.40 | 40.20 | 55.48 | 70.40 | 40.20 | 56.20 | 70.60 |
| | **Case** | **Control** | **Total** | **Case** | **Control** | **Total** | **Case** | **Control** | **Total** | **Case** | **Control** | **Total** | **Case** | **Control** | **Total** |
| **CIDI Depression** | 3226 | 6125 | 9351 | 4395 | 7552 | 11947 | 4346 | 6774 | 11120 | 2710 | 4988 | 7698 | 4974 | 10030 | 15004 |
| **Broad Depression** | 4494 | 6114 | 10608 | 5960 | 7538 | 13498 | 5828 | 6992 | 12820 | 3551 | 5122 | 8673 | 6852 | 10230 | 17082 |
| **Neuroticism** | **Mean** | **Total** | | **Mean** | **Total** | | **Mean** | **Total** | | **Mean** | **Total** | | **Mean** | **Total** | |
| | 4.25 | 9826 | | 4.24 | 12213 | | 4.32 | 11689 | | 4.20 | 7940 | | 4.06 | 15598 | |

*Abbreviations.* Nind, number of individuals; Min, minimum; Max, maximum.

**eTable 30.** Cluster Demographics Using All Male Participants With Complete Trauma Exposure Responses

| DEMOGRPAHIC | NORTH CLUSTER | | | MIDNORTH CLUSTER | | | MIDSOUTH CLUSTER | | | SOUTHWEST CLUSTER | | | SOUTHEAST CLUSTER | | |
|---|---|---|---|---|---|---|---|---|---|---|---|---|---|---|---|
| **Total Nind** | 10854 | | | 13386 | | | 13126 | | | 8163 | | | 15724 | | |
| **Age** | **Min** | **Mean** | **Max** | **Min** | **Mean** | **Max** | **Min** | **Mean** | **Max** | **Min** | **Mean** | **Max** | **Min** | **Mean** | **Max** |
| | 40.00 | 56.87 | 70.30 | 40.30 | 57.12 | 72.50 | 40.10 | 57.64 | 72.90 | 40.30 | 56.74 | 70.40 | 38.90 | 57.18 | 70.40 |
| | **Case** | **Control** | **Total** | **Case** | **Control** | **Total** | **Case** | **Control** | **Total** | **Case** | **Control** | **Total** | **Case** | **Control** | **Total** |
| **CIDI Depression** | 1814 | 7479 | 9293 | 2277 | 9054 | 11331 | 2282 | 8795 | 11077 | 1371 | 5623 | 6994 | 2342 | 11073 | 13415 |
| **Broad Depression** | 2654 | 7418 | 10072 | 3546 | 8897 | 12443 | 3365 | 8876 | 12241 | 1989 | 5634 | 7623 | 3587 | 11105 | 14692 |
| **Neuroticism** | **Mean** | **Total** | | **Mean** | **Total** | | **Mean** | **Total** | | **Mean** | **Total** | | **Mean** | **Total** | |
| | 3.41 | 9337 | | 3.38 | 11438 | | 3.36 | 11302 | | 3.31 | 7019 | | 3.32 | 13645 | |

*Abbreviations.* Nind, number of individuals; Min, minimum; Max, maximum.

**eTable 31.** Cluster Demographics Using Unrelated Male Participants With Complete Trauma Exposure Responses

| DEMOGRPAHIC | NORTH CLUSTER | | | MIDNORTH CLUSTER | | | MIDSOUTH CLUSTER | | | SOUTHWEST CLUSTER | | | SOUTHEAST CLUSTER | | |
|---|---|---|---|---|---|---|---|---|---|---|---|---|---|---|---|
| Total Nind | 9368 | | | 11837 | | | 11643 | | | 7368 | | | 14393 | | |
| Age | **Min** | **Mean** | **Max** | **Min** | **Mean** | **Max** | **Min** | **Mean** | **Max** | **Min** | **Mean** | **Max** | **Min** | **Mean** | **Max** |
| | 40.00 | 56.85 | 70.30 | 40.30 | 57.08 | 72.50 | 40.10 | 57.59 | 72.90 | 40.30 | 56.73 | 70.40 | 40.20 | 57.13 | 70.40 |
| | **Case** | **Control** | **Total** | **Case** | **Control** | **Total** | **Case** | **Control** | **Total** | **Case** | **Control** | **Total** | **Case** | **Control** | **Total** |
| CIDI Depression | 1575 | 6461 | 8036 | 2020 | 8007 | 10027 | 2065 | 7775 | 9840 | 1244 | 5066 | 6310 | 2131 | 10165 | 12296 |
| Broad Depression | 2287 | 6395 | 8682 | 3148 | 7855 | 11003 | 3002 | 7858 | 10860 | 1789 | 5090 | 6879 | 3253 | 10195 | 13448 |
| Neuroticism | **Mean** | **Total** | | **Mean** | **Total** | | **Mean** | **Total** | | **Mean** | **Total** | | **Mean** | **Total** | |
| | 3.41 | 8074 | | 3.38 | 10118 | | 3.36 | 10019 | | 3.31 | 6351 | | 3.31 | 12481 | |

*Abbreviations.* Nind, number of individuals; Min, minimum; Max, maximum.

© 2022 Chuong M et al. *JAMA Psychiatry.*

**eAppendix 1.**

OmicS-data-based Complex-trait Analysis OSCA v0.45 is a software used to analyse available omics (e.g. DNA methylation) data [17]. Three separate equations are provided to compute Omics Relationship Matrices (ORMs). In this study, we refer to ORMs as environmental relationship matrices (Es), as this is more appropriate for the data being used in this study (trauma principal components).

Algorithm 1 computes similarity by using standardised DNAm measures of all probes. Here it is important to note that the DNAm measures will be replaced with the principal component eigenvectors (PCs) of our trauma exposure measures.

$$\textbf{Equation 1}.\ A_{jk} = \Sigma_i(x_{ij} - \mu_i)(x_{ik} - \mu_i) / \sigma_i^2$$

With $A_{jk}$ being the environmental similarity between individuals $j$ and $k$. Where $x_{ij}$ and $x_{ik}$ is the **standardised** $\mathrm{PC}_i$ in individual $j$ and $k$, respectively. $\mu_i$ and $\sigma_i^2$ are the mean and variance of $\mathrm{PC}_i$ over all the individuals respectively.

Algorithm 2 computes similarity by using **unstandardised** eigenvectorss and factors in the number of eigenvectors utilised. This algorithm implicitly assumes eigenvectors with smaller variance tend to have larger effects on the phenotype, and that there is no relationship between the depression/neuroticism phenotypic variance captured by the eigenvector and the variance of the eigenvector. Here, we cannot confirm these assumptions, so equation 2, was deemed inappropriate for the data we have.

$$\textbf{Equation 2}.\ A_{jk} = \frac{1}{m}\Sigma_i(x_{ij} - \mu_i)(x_{ik} - \mu_i) / \sigma_i^2$$

With $A_{jk}$ being the environmental similarity between individuals $j$ and $k$. Where $x_{ij}$ and $x_{ik}$ is the **unstandardised** $\mathrm{PC}_i$ in individual $j$ and $k$, respectively. $\mu_i$ and $\sigma_i^2$ are the mean and variance of $\mathrm{PC}_i$ over all the individuals respectively and $m$ is the number of PCs utilised.

Algorithm 3 computes similarity by iteratively standardising eigenvectors and across individuals.

$$\textbf{Equation 3}.\ A_{jk} = \frac{1}{m}\Sigma_i(x_{ij} - \mu_i)(x_{ik} - \mu_i) / \Sigma_i\sigma_i^2$$

© 2022 Chuong M et al. *JAMA Psychiatry.*

With $A_{jk}$ being the environmental similarity between individuals $j$ and $k$. Where $x_{ij}$ and $x_{ik}$ is the **standardised** $\mathrm{PC}_i$ in individual $j$ and $k$, respectively. $\mu_i$ and $\sigma_i^2$ are the mean and variance of $\mathrm{PC}_i$ over all the individuals respectively.

For our study, all algorithms were separately used to compute Es from full trauma eigenvectors (PCs), algorithm 1 and 3 were deemed the most appropriate for our data. Subtle differences were observed in outputted relationship matrices (**Figure SA1**). Matrices computed using algorithm 1 present diagonal values with a mean of 1, whereas matrices computed using algorithm 3 present diagonal values of 1.

| Individuals | 1 | 2 | 3 | 4… |
|---|---|---|---|---|
| 1 | 0.989 | | | |
| 2 | 0.002 | 0.758 | | |
| 3 | 0.031 | 0.004 | 0.632 | |
| 4… | 0.013 | 0.003 | 0.031 | 0.944 |

| Individuals | 1 | 2 | 3 | 4… |
|---|---|---|---|---|
| 1 | 1.00 | | | |
| 2 | 0.002 | 1.00 | | |
| 3 | 0.301 | 0.104 | 1.00 | |
| 4… | 0.003 | 0.023 | 0.231 | 1.00 |

***Figure SA1.*** Trauma Exposure Relationship Matrices. Matrix diagonals represent similarity between an individual with themselves. Offdiagonals represent pairwise similarity between individuals within the sample. Here, similarity is computed using available trauma exposure principal components using OSCA software. Left, shows a representative similarity matrix where similarity is computed using Algorithm 1. Right, shows a representative similarity matrix where similarity is computed using Algorithm 3. Please note values specified are not obtained from real matrices.

Offdiagonal values of the computed Es and genomic relationship matrices (Gs) were plotted to visualise the relationship between values (**Figure SA2**).

© 2022 Chuong M et al. *JAMA Psychiatry.*

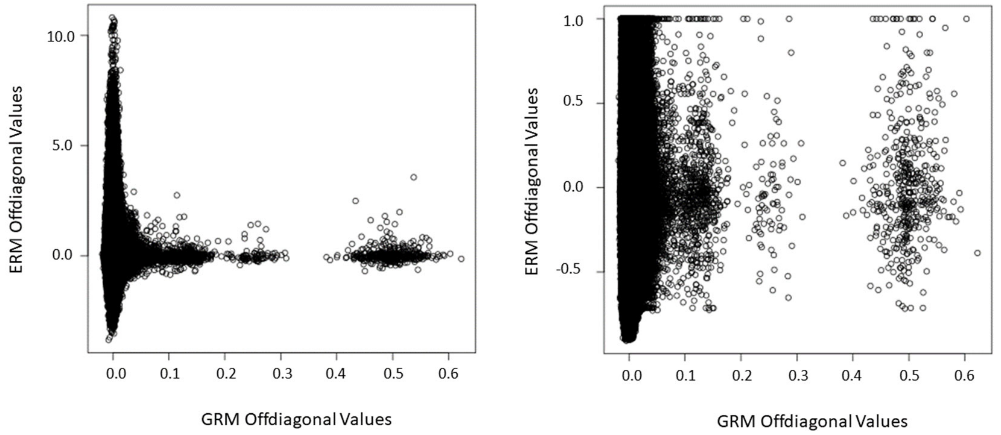


***Figure SA2*.** Offdiagonal Values of UKB North Geographical Cluster Genomic and Environmental Relationship Matrices. Left, Environmental Relationship Matrix (ERM) computed using Algorithm 1 of OSCA software. Right, ERM computed using Algorithm 3 of OSCA software. The x-axis represent offdiagonal genomic similarity values, cluster points surrounding points 0.2-0.3 and 0.5 show points of relatives. The y-axis represents offdiagonal trauma exposure similarity values.

Zhang and colleagues (2019) demonstrated, with simulation analyses, that mixed linear model anlaysis results obtained from matrices computed with algorithm 1 and 3 showed minimal differences. Similarly, we show that GREML outputs were negligibly different between the algorithms of interest (**eTable 32**).

Here, algorithm 1 is opted when computing the ERMs used for downstream analyses. The mixed linear model results are similar and the plots from **figure 2** suggest values in line with expectations.

**eTable 32** presents results of mixed linear models utilising genomic relationship matrices, trauma exposure relationship matrices and genome-by-trauma exposure relationship matrices as random effects. Results include estimates of CIDI depression variance attributable to each random effect, standard errors, log-ratio test values and log-ratio test p-values signifying model utility. Table 7 presents results of mixed linear models whereby trauma exposure relationship matrices were computed using the 3 different algorithms available within OmicS-data-based Complex-trait Analysis OSCA software.

© 2022 Chuong M et al. *JAMA Psychiatry.*

**eTable 32.** Mixed Linear Model Results of Proportion of CIDI Depression Variance Attributable to Environmental Relationship Matrices Computed Using Different OSCA Algorithms

| OSCA | Trait | Model | Source | NORTH | | | | MIDNORTH | | | | MIDSOUTH | | | | SOUTHWEST | | | | SOUTHEAST | | | |
|---|---|---|---|---|---|---|---|---|---|---|---|---|---|---|---|---|---|---|---|---|---|---|---|
| Algorithm | | | | Variance | SE | LRT | LRT-P | Variance | SE | LRT | LRT-P | Variance | SE | LRT | LRT-P | Variance | SE | LRT | LRT-P | Variance | SE | LRT | LRT-P |
| 1 | CIDI | G | G | 0.195 | 0.037 | 28.956 | 0.000 | 0.134 | 0.030 | 20.793 | 0.000 | 0.114 | 0.030 | 15.209 | 0.000 | 0.218 | 0.048 | 21.359 | 0.000 | 0.121 | 0.027 | 20.707 | 0.000 |
| | | E | E | 0.145 | 0.047 | 1801.636 | 0.000 | 0.159 | 0.050 | 2528.312 | 0.000 | 0.147 | 0.047 | 2299.555 | 0.000 | 0.140 | 0.046 | 2299.555 | 0.000 | 0.159 | 0.051 | 2969.008 | 0.000 |
| | | G + E | G | 0.146 | 0.034 | | | 0.084 | 0.027 | | | 0.070 | 0.027 | | | 0.141 | 0.043 | | | 0.063 | 0.024 | | |
| | | | E | 0.144 | 0.047 | 1792.306 | 0.000 | 0.159 | 0.050 | 2517.636 | 0.000 | 0.147 | 0.047 | 2291.178 | 0.000 | 0.140 | 0.045 | 1373.706 | 0.000 | 0.159 | 0.051 | 2955.386 | 0.000 |
| | | G x E | G | 0.148 | 0.033 | | | 0.082 | 0.026 | | | 0.065 | 0.027 | | | 0.135 | 0.043 | | | 0.044 | 0.044 | | |
| | | | E | 0.150 | 0.048 | | | 0.162 | 0.051 | | | 0.150 | 0.048 | | | 0.140 | 0.045 | | | 0.163 | 0.052 | | |
| | | | GxE | 0.205 | 0.018 | 159.722 | 0.000 | 0.141 | 0.015 | 106.471 | 0.000 | 0.126 | 0.014 | 90.449 | 0.000 | 0.166 | 0.019 | 80.735 | 0.000 | 0.259 | 0.023 | 155.156 | 0.000 |
| 3 | CIDI | G | G | 0.195 | 0.037 | 28.956 | 0.000 | 0.134 | 0.030 | 20.793 | 0.000 | 0.114 | 0.030 | 15.209 | 0.000 | 0.218 | 0.048 | 21.359 | 0.000 | 0.014 | 0.003 | 20.707 | 0.000 |
| | | E | E | 0.169 | 0.056 | 1362.035 | 0.000 | 0.175 | 0.057 | 1740.767 | 0.000 | 0.153 | 0.050 | 1599.097 | 0.000 | 0.136 | 0.046 | 883.480 | 0.000 | 0.144 | 0.048 | 1948.929 | 0.000 |
| | | G + E | G | 0.163 | 0.034 | | | 0.085 | 0.026 | | | 0.074 | 0.027 | | | 0.169 | 0.044 | | | 0.069 | 0.024 | | |
| | | | E | 0.168 | 0.055 | 1358.782 | 0.000 | 0.174 | 0.057 | 2517.636 | 0.000 | 0.152 | 0.050 | 1591.741 | 0.000 | 0.135 | 0.046 | 877.686 | 0.000 | 0.143 | 0.048 | 1936.461 | 0.000 |
| | | G x E | G | 0.164 | 0.034 | | | 0.084 | 0.026 | | | 0.074 | 0.027 | | | 0.169 | 0.044 | | | 0.060 | 0.046 | | |
| | | | E | 0.168 | 0.055 | | | 0.174 | 0.057 | | | 0.152 | 0.050 | | | 0.135 | 0.046 | | | 0.141 | 0.048 | | |
| | | | GxE | 0.065 | 0.092 | 0.445 | 0.252 | 0.119 | 0.075 | 2.387 | 0.061 | 0.025 | 0.073 | 0.111 | 0.370 | 0.021 | 0.120 | 0.028 | 0.434 | 0.077 | 0.132 | 0.335 | 0.281 |
| 2 | CIDI | G | G | 0.195 | 0.037 | 28.956 | 0.000 | 0.134 | 0.030 | 20.793 | 0.000 | 0.114 | 0.030 | 15.209 | 0.000 | 0.218 | 0.048 | 21.359 | 0.000 | 0.014 | 0.003 | 20.707 | 0.000 |
| | | E | E | 0.173 | 0.002 | 1811.054 | 0.000 | 0.176 | 0.002 | 2539.357 | 0.000 | 0.180 | 0.002 | 2310.492 | 0.000 | 0.176 | 0.002 | 1396.145 | 0.000 | 0.166 | 0.001 | 2980.101 | 0.000 |
| | | G + E | G | 0.151 | 0.035 | | | 0.088 | 0.028 | | | 0.073 | 0.028 | | | 0.148 | 0.045 | | | 0.066 | 0.025 | | |
| | | | E | 0.094 | 0.032 | 1801.649 | 0.000 | 0.095 | 0.032 | 2528.653 | 0.000 | 0.088 | 0.029 | 2302.109 | 0.000 | 0.078 | 0.027 | 1385.673 | 0.000 | 0.093 | 0.031 | 2966.475 | 0.000 |
| | | G x E | G | 0.154 | 0.035 | | | 0.087 | 0.028 | | | 0.068 | 0.028 | | | 0.142 | 0.045 | | | 0.048 | 0.046 | | |
| | | | E | 0.096 | 0.032 | | | 0.096 | 0.032 | | | 0.089 | 0.030 | | | 0.078 | 0.027 | | | 0.097 | 0.033 | | |
| | | | GxE | 0.164 | 0.017 | 110.361 | 0.000 | 0.093 | 0.013 | 49.731 | 0.000 | 0.092 | 0.013 | 51.669 | 0.000 | 0.125 | 0.019 | 45.357 | 0.000 | 0.212 | 0.022 | 103.159 | 0.000 |

*Abbreviations.* CIDI, Composite International Diagnostic Inventory Depression; OSCA, OmicS-based-data Complex-trait Analysis; G, Genomic Relationship Matrix; E, Environmental (trauma exposure) Relationship Matrix; GxE, Genome-by-Trauma Exposure Interaction Relationship Matrix; SE, standard error; LRT, log-ratio test; P, P-Value.

© 2022 Chuong M et al. *JAMA Psychiatry.*

**eTable 33** presents heritability estimates obtained for trauma exposure obtained from Haseman-Elston regression models. The table presents estimates of variance attributable to the genomic relationship matrix, standard errors and P-Values from analyses conducted on the whole and unrelated samples separately. Heritability estimates were obtained separately for full trauma, childhood, adult and catastrophic trauma exposure.

**eTable 33.** Trauma Exposure Heritability Estimates

| | | Full Sample | | | | Unrelated Sample | | | |
|---|---|---|---|---|---|---|---|---|---|
| **Trauma** | **Cluster** | **Variance** | **SE** | **P** | **N** | **Variance** | **SE** | **P** | **N** |
| Full Trauma | North | 0.173 | 0.021 | | 24287 | 0.174 | 0.024 | | 20994 |
| | Midnorth | 0.166 | 0.017 | | 29981 | 0.149 | 0.019 | | 26566 |
| | Midsouth | 0.163 | 0.017 | | 28961 | 0.160 | 0.020 | | 25691 |
| | Southwest | 0.159 | 0.026 | | 18696 | 0.149 | 0.030 | | 16841 |
| | Southeast | 0.177 | 0.014 | | 36323 | 0.168 | 0.016 | | 33128 |
| | meta-A | 0.169 | 0.008 | 0.000 | | 0.161 | 0.009 | 0.000 | |
| Childhood | North | 0.156 | 0.021 | | 24287 | 0.138 | 0.024 | | 20994 |
| | Midnorth | 0.125 | 0.017 | | 29981 | 0.108 | 0.019 | | 26566 |
| | Midsouth | 0.151 | 0.017 | | 28961 | 0.140 | 0.019 | | 25691 |
| | Southwest | 0.151 | 0.026 | | 18696 | 0.140 | 0.029 | | 16841 |
| | Southeast | 0.149 | 0.014 | | 36323 | 0.134 | 0.015 | | 33128 |
| | meta-A | 0.145 | 0.008 | 0.000 | | 0.131 | 0.009 | 0.000 | |
| Adult | North | 0.063 | 0.020 | | 24287 | 0.073 | 0.024 | | 20994 |
| | Midnorth | 0.068 | 0.017 | | 29981 | 0.061 | 0.019 | | 26566 |
| | Midsouth | 0.064 | 0.017 | | 28961 | 0.077 | 0.019 | | 25691 |
| | Southwest | 0.036 | 0.026 | | 18696 | 0.054 | 0.030 | | 16841 |
| | Southeast | 0.066 | 0.014 | | 36323 | 0.068 | 0.015 | | 33128 |
| | meta-A | 0.063 | 0.008 | 0.000 | | 0.068 | 0.009 | 0.000 | |
| Catastrophic | North | 0.100 | 0.020 | | 24287 | 0.099 | 0.024 | | 20994 |
| | Midnorth | 0.093 | 0.017 | | 29981 | 0.083 | 0.019 | | 26566 |
| | Midsouth | 0.119 | 0.017 | | 28961 | 0.120 | 0.019 | | 25691 |
| | Southwest | 0.135 | 0.026 | | 18696 | 0.129 | 0.029 | | 16841 |
| | Southeast | 0.098 | 0.014 | | 36323 | 0.093 | 0.015 | | 33128 |
| | meta-A | 0.105 | 0.008 | 0.000 | | 0.101 | 0.009 | 0.000 | |

*Abbreviations.* SE, standard error, P, P-Value; N, sample size; meta-A, Meta-analysed estimates of variance and SEs.

© 2022 Chuong M et al. *JAMA Psychiatry.*

**eTable 34** presents estimates of genetic correlations between trauma exposure and depression/neuroticism variables. Tables present estimates, standard errors and P-Values.

**eTable 34.** Trauma Exposure and Depression/Neuroticism Genetic Correlations

| | | CIDI | | | BROAD | | | NEUROTICISM | | |
|---|---|---|---|---|---|---|---|---|---|---|
| **TRAUMA** | **CLUSTER** | **Est** | **SE** | **P** | **Est** | **SE** | **P** | **Est** | **SE** | **P** |
| **TRAUMA** | North | 0.506 | 0.171 | | 0.475 | 0.216 | | 0.289 | 0.219 | |
| | Midnorth | 0.721 | 0.167 | | 0.340 | 0.156 | | 0.277 | 0.116 | |
| | Midsouth | 0.636 | 0.198 | | 0.413 | 0.187 | | 0.229 | 0.141 | |
| | Southwest | 0.614 | 0.276 | | 0.730 | 0.317 | | 0.408 | 0.276 | |
| | Southeast | 0.672 | 0.186 | | 0.307 | 0.147 | | 0.447 | 0.110 | |
| | Meta-A | 0.632 | 0.085 | 0.000 | 0.390 | 0.082 | 0.000 | 0.333 | 0.064 | 0.000 |
| **CHILDHOOD** | North | 0.430 | 0.181 | | 0.374 | 0.226 | | 0.229 | 0.230 | |
| | Midnorth | 0.765 | 0.187 | | 0.404 | 0.177 | | 0.369 | 0.131 | |
| | Midsouth | 0.668 | 0.229 | | 0.465 | 0.216 | | 0.282 | 0.155 | |
| | Southwest | 0.570 | 0.290 | | 0.621 | 0.331 | | 0.473 | 0.277 | |
| | Southeast | 0.604 | 0.184 | | 0.141 | 0.154 | | 0.332 | 0.120 | |
| | Meta-A | 0.605 | 0.091 | 0.000 | 0.337 | 0.090 | 0.000 | 0.332 | 0.071 | 0.000 |
| **ADULT** | North | 0.638 | 0.267 | | 0.567 | 0.330 | | 0.351 | 0.331 | |
| | Midnorth | 0.668 | 0.243 | | 0.234 | 0.230 | | 0.091 | 0.175 | |
| | Midsouth | 0.599 | 0.280 | | 0.451 | 0.269 | | 0.272 | 0.214 | |
| | Southwest | 0.956 | 0.748 | | 1.336 | 0.950 | | 0.416 | 0.658 | |
| | Southeast | 0.630 | 0.312 | | 0.221 | 0.266 | | 0.476 | 0.201 | |
| | Meta-A | 0.647 | 0.134 | 0.000 | 0.358 | 0.132 | 0.007 | 0.274 | 0.105 | 0.009 |
| **CATASTROPHIC** | North | 0.527 | 0.230 | | 0.553 | 0.295 | | 0.217 | 0.295 | |
| | Midnorth | 0.657 | 0.213 | | 0.224 | 0.202 | | 0.225 | 0.161 | |
| | Midsouth | 0.537 | 0.240 | | 0.092 | 0.215 | | -0.080 | 0.166 | |
| | Southwest | 0.307 | 0.306 | | 0.013 | 0.332 | | 0.225 | 0.285 | |
| | Southeast | 0.535 | 0.208 | | 0.489 | 0.163 | | 0.372 | 0.137 | |
| | Meta-A | 0.536 | 0.104 | 0.000 | 0.310 | 0.098 | 0.002 | 0.204 | 0.081 | 0.012 |

*Abbreviations.* CIDI, Composite International Diagnostic Inventory definition of depression; Est, estimate; SE, standard error; P, P-Value; Meta-A, meta analysed values of estimates and SEs.

© 2022 Chuong M et al. *JAMA Psychiatry.*

**eTables 35-46** present results of mixed linear models using genomic relationship matrices (Gs), environmental relationship matrices (Es) using trauma exposure eigenvectors (PCs), and genome-by-trauma exposure interaction relationship matrices (GxE) as random effects for each geographical cluster. Results include estimates of trait variance attributable to the G, E and GxE, standard errors and P-Values. Results also include meta-analysed estimates and standard errors. Each table presents results of models utilising either different samples (unrelated, female only, males only) or different Es (using full trauma exposure principal components PCs, childhood trauma exposure PCs, adult trauma exposure PCs etc.). Details of the samples and Es utilised are available in table headings.

© 2022 Chuong M et al. *JAMA Psychiatry.*

**eTable 35.** Mixed Linear Model Results Including Es of Full Trauma Exposure Principal Components

| Trait | Model | Source | META ANALYSIS | | | NORTH | | | | MIDNORTH | | | | MIDSOUTH | | | | SOUTHWEST | | | | SOUTHEAST | | | |
|---|---|---|---|---|---|---|---|---|---|---|---|---|---|---|---|---|---|---|---|---|---|---|---|---|---|
| | | | Variance | SE | P-Value | Variance | SE | LRT | LRT-P | Variance | SE | LRT | LRT-P | Variance | SE | LRT | LRT-P | Variance | SE | LRT | LRT-P | Variance | SE | LRT | LRT-P |
| CIDI | G | G | 0.167 | 0.017 | 0.000 | 0.229 | 0.044 | 28.956 | 0.000 | 0.158 | 0.035 | 20.793 | 0.000 | 0.134 | 0.035 | 15.209 | 0.000 | 0.256 | 0.056 | 21.359 | 0.000 | 0.142 | 0.032 | 20.707 | 0.000 |
| | E | E | 0.176 | 0.025 | 0.000 | 0.170 | 0.055 | 1801.636 | 0.000 | 0.187 | 0.059 | 2528.312 | 0.000 | 0.173 | 0.055 | 2299.555 | 0.000 | 0.165 | 0.053 | 1384.156 | 0.000 | 0.187 | 0.060 | 2969.008 | 0.000 |
| | G + E | G | 0.104 | 0.015 | 0.000 | 0.171 | 0.040 | | | 0.098 | 0.031 | | | 0.082 | 0.032 | | | 0.166 | 0.051 | | | 0.074 | 0.028 | | |
| | | E | 0.175 | 0.025 | 0.000 | 0.169 | 0.055 | 1792.306 | 0.000 | 0.186 | 0.059 | 2517.636 | 0.000 | 0.172 | 0.055 | 2291.178 | 0.000 | 0.164 | 0.053 | 1373.706 | 0.000 | 0.187 | 0.060 | 2955.386 | 0.000 |
| | G x E | G | 0.098 | 0.015 | 0.000 | 0.174 | 0.039 | | | 0.096 | 0.031 | | | 0.076 | 0.031 | | | 0.158 | 0.050 | | | 0.062 | 0.027 | | |
| | | E | 0.178 | 0.026 | 0.000 | 0.176 | 0.057 | | | 0.190 | 0.060 | | | 0.176 | 0.056 | | | 0.165 | 0.053 | | | 0.188 | 0.060 | | |
| | | GxE | 0.201 | 0.009 | 0.000 | 0.241 | 0.021 | 159.722 | 0.000 | 0.165 | 0.017 | 106.471 | 0.000 | 0.148 | 0.017 | 90.449 | 0.000 | 0.195 | 0.023 | 80.735 | 0.000 | 0.294 | 0.020 | 295.840 | 0.000 |
| BROAD | G | G | 0.135 | 0.014 | 0.000 | 0.154 | 0.036 | 19.061 | 0.000 | 0.160 | 0.029 | 32.247 | 0.000 | 0.110 | 0.030 | 14.595 | 0.000 | 0.159 | 0.048 | 11.076 | 0.000 | 0.118 | 0.026 | 21.365 | 0.000 |
| | E | E | 0.105 | 0.016 | 0.000 | 0.104 | 0.035 | 1301.673 | 0.000 | 0.106 | 0.035 | 1657.122 | 0.000 | 0.100 | 0.034 | 1537.483 | 0.000 | 0.107 | 0.036 | 1021.711 | 0.000 | 0.110 | 0.037 | 2094.855 | 0.000 |
| | G + E | G | 0.098 | 0.013 | 0.000 | 0.122 | 0.034 | | | 0.126 | 0.027 | | | 0.076 | 0.028 | | | 0.119 | 0.045 | | | 0.073 | 0.024 | | |
| | | E | 0.105 | 0.016 | 0.000 | 0.104 | 0.035 | 1296.487 | 0.000 | 0.106 | 0.035 | 1648.216 | 0.000 | 0.100 | 0.033 | 1531.056 | 0.000 | 0.107 | 0.036 | 1017.853 | 0.000 | 0.110 | 0.037 | 2083.518 | 0.000 |
| | G x E | G | 0.097 | 0.013 | 0.000 | 0.121 | 0.034 | | | 0.127 | 0.027 | | | 0.076 | 0.028 | | | 0.116 | 0.044 | | | 0.074 | 0.024 | | |
| | | E | 0.106 | 0.016 | 0.000 | 0.105 | 0.035 | | | 0.107 | 0.036 | | | 0.100 | 0.034 | | | 0.107 | 0.036 | | | 0.111 | 0.037 | | |
| | | GxE | 0.048 | 0.006 | 0.000 | 0.051 | 0.015 | 12.320 | 0.000 | 0.027 | 0.013 | 4.269 | 0.019 | 0.021 | 0.013 | 2.648 | 0.052 | 0.072 | 0.018 | 15.423 | 0.000 | 0.087 | 0.014 | 41.422 | 0.000 |
| NEURO | G | G | 0.150 | 0.009 | 0.000 | 0.102 | 0.024 | 19.868 | 0.000 | 0.174 | 0.020 | 81.415 | 0.000 | 0.147 | 0.021 | 54.107 | 0.000 | 0.148 | 0.031 | 24.168 | 0.000 | 0.160 | 0.017 | 94.024 | 0.000 |
| | E | E | 0.060 | 0.009 | 0.000 | 0.061 | 0.020 | 1221.472 | 0.000 | 0.066 | 0.022 | 1539.876 | 0.000 | 0.061 | 0.021 | 1420.245 | 0.000 | 0.054 | 0.018 | 789.554 | 0.000 | 0.061 | 0.020 | 1906.571 | 0.000 |
| | G + E | G | 0.132 | 0.009 | 0.000 | 0.092 | 0.022 | | | 0.144 | 0.019 | | | 0.131 | 0.019 | | | 0.129 | 0.029 | | | 0.144 | 0.016 | | |
| | | E | 0.060 | 0.009 | 0.000 | 0.061 | 0.020 | 1220.160 | 0.000 | 0.065 | 0.022 | 1522.604 | 0.000 | 0.061 | 0.020 | 1416.278 | 0.000 | 0.054 | 0.018 | 786.084 | 0.000 | 0.061 | 0.020 | 1899.179 | 0.000 |
| | G x E | G | 0.132 | 0.009 | 0.000 | 0.091 | 0.022 | | | 0.147 | 0.019 | | | 0.134 | 0.019 | | | 0.131 | 0.029 | | | 0.143 | 0.016 | | |
| | | E | 0.061 | 0.009 | 0.000 | 0.062 | 0.021 | | | 0.066 | 0.022 | | | 0.062 | 0.021 | | | 0.054 | 0.019 | | | 0.062 | 0.021 | | |
| | | GxE | 0.072 | 0.004 | 0.000 | 0.063 | 0.010 | 51.022 | 0.000 | 0.075 | 0.009 | 80.387 | 0.000 | 0.073 | 0.009 | 76.109 | 0.000 | 0.080 | 0.012 | 54.876 | 0.000 | 0.071 | 0.008 | 84.435 | 0.000 |

*Abbreviations.* E, environmental relationship matrix using full trauma principal components; SE, standard error; LRT, log-ratio test value; LRT-P, log-ratio test P-Value; G, genetic (Genomic Relationship Matrix); E, environmental (full trauma exposure ERM); GxE, genome-by-trauma exposure interaction.

© 2022 Chuong M et al. *JAMA Psychiatry.*

**eTable 36.** Mixed Linear Model Results Including Es of Childhood Trauma Exposure Principal Components

| Trait | Model | Source | META ANALYSIS | | | NORTH | | | | MIDNORTH | | | | MIDSOUTH | | | | SOUTHWEST | | | | SOUTHEAST | | | |
|---|---|---|---|---|---|---|---|---|---|---|---|---|---|---|---|---|---|---|---|---|---|---|---|---|---|
| | | | Variance | SE | P-Value | Variance | SE | LRT | LRT-P | Variance | SE | LRT | LRT-P | Variance | SE | LRT | LRT-P | Variance | SE | LRT | LRT-P | Variance | SE | LRT | LRT-P |
| CIDI | G | G | 0.163 | 0.016 | 0.000 | 0.219 | 0.042 | 28.960 | 0.000 | 0.140 | 0.033 | 17.998 | 0.000 | 0.144 | 0.034 | 18.766 | 0.000 | 0.243 | 0.054 | 21.238 | 0.000 | 0.144 | 0.030 | 23.045 | 0.000 |
| | E | E | 0.101 | 0.027 | 0.000 | 0.092 | 0.055 | 1015.673 | 0.000 | 0.104 | 0.062 | 1482.339 | 0.000 | 0.097 | 0.058 | 1372.832 | 0.000 | 0.102 | 0.061 | 918.242 | 0.000 | 0.112 | 0.067 | 1866.562 | 0.000 |
| | G + E | G | 0.113 | 0.015 | 0.000 | 0.174 | 0.040 | | | 0.095 | 0.031 | | | 0.099 | 0.032 | | | 0.171 | 0.051 | | | 0.090 | 0.029 | | |
| | | E | 0.100 | 0.027 | 0.000 | 0.091 | 0.055 | 1006.830 | 0.000 | 0.104 | 0.062 | 1473.776 | 0.000 | 0.097 | 0.058 | 1363.960 | 0.000 | 0.101 | 0.061 | 908.968 | 0.000 | 0.112 | 0.067 | 1853.858 | 0.000 |
| | G x E | G | 0.116 | 0.017 | 0.000 | 0.177 | 0.040 | | | 0.096 | 0.031 | | | 0.095 | 0.032 | | | 0.164 | 0.050 | | | 0.073 | 0.053 | | |
| | | E | 0.102 | 0.027 | 0.000 | 0.093 | 0.056 | | | 0.106 | 0.063 | | | 0.098 | 0.059 | | | 0.104 | 0.062 | | | 0.114 | 0.068 | | |
| | | GxE | 0.084 | 0.006 | 0.000 | 0.096 | 0.013 | 66.250 | 0.000 | 0.079 | 0.011 | 61.123 | 0.000 | 0.056 | 0.011 | 31.394 | 0.000 | 0.100 | 0.015 | 55.787 | 0.000 | 0.126 | 0.017 | 68.423 | 0.000 |
| BROAD | G | G | 0.135 | 0.014 | 0.000 | 0.149 | 0.034 | 19.829 | 0.000 | 0.142 | 0.028 | 27.752 | 0.000 | 0.115 | 0.028 | 17.267 | 0.000 | 0.167 | 0.046 | 13.392 | 0.000 | 0.128 | 0.025 | 27.266 | 0.000 |
| | E | E | 0.059 | 0.016 | 0.000 | 0.056 | 0.034 | 749.802 | 0.000 | 0.058 | 0.035 | 949.004 | 0.000 | 0.059 | 0.036 | 988.282 | 0.000 | 0.061 | 0.038 | 636.928 | 0.000 | 0.064 | 0.039 | 1308.214 | 0.000 |
| | G + E | G | 0.109 | 0.013 | 0.000 | 0.122 | 0.033 | | | 0.116 | 0.027 | | | 0.093 | 0.027 | | | 0.134 | 0.044 | | | 0.103 | 0.024 | | |
| | | E | 0.059 | 0.016 | 0.000 | 0.055 | 0.034 | 744.447 | 0.000 | 0.057 | 0.035 | 941.854 | 0.000 | 0.059 | 0.036 | 983.444 | 0.000 | 0.061 | 0.037 | 632.895 | 0.000 | 0.064 | 0.039 | 1300.637 | 0.000 |
| | G x E | G | 0.109 | 0.015 | 0.000 | 0.122 | 0.033 | | | 0.116 | 0.027 | | | 0.093 | 0.027 | | | 0.134 | 0.044 | | | 0.086 | 0.045 | | |
| | | E | 0.059 | 0.016 | 0.000 | 0.056 | 0.034 | | | 0.058 | 0.035 | | | 0.059 | 0.036 | | | 0.062 | 0.038 | | | 0.063 | 0.039 | | |
| | | GxE | 0.020 | 0.004 | 0.000 | 0.022 | 0.009 | 6.399 | 0.006 | 0.015 | 0.008 | 3.413 | 0.032 | 0.006 | 0.008 | 0.585 | 0.222 | 0.038 | 0.011 | 11.776 | 0.000 | 0.041 | 0.012 | 13.243 | 0.000 |
| NEURO | G | G | 0.150 | 0.009 | 0.000 | 0.099 | 0.023 | 20.378 | 0.000 | 0.174 | 0.019 | 89.340 | 0.000 | 0.153 | 0.020 | 62.963 | 0.000 | 0.141 | 0.030 | 23.680 | 0.000 | 0.159 | 0.016 | 98.967 | 0.000 |
| | E | E | 0.041 | 0.011 | 0.000 | 0.043 | 0.026 | 932.418 | 0.000 | 0.044 | 0.027 | 1119.272 | 0.000 | 0.042 | 0.025 | 1049.332 | 0.000 | 0.036 | 0.022 | 583.258 | 0.000 | 0.040 | 0.025 | 1326.980 | 0.000 |
| | G + E | G | 0.138 | 0.009 | 0.000 | 0.095 | 0.022 | | | 0.153 | 0.019 | | | 0.143 | 0.019 | | | 0.129 | 0.029 | | | 0.149 | 0.016 | | |
| | | E | 0.040 | 0.011 | 0.000 | 0.043 | 0.026 | 932.508 | 0.000 | 0.044 | 0.027 | 1105.278 | 0.000 | 0.042 | 0.025 | 1047.271 | 0.000 | 0.036 | 0.022 | 580.974 | 0.000 | 0.040 | 0.025 | 1322.971 | 0.000 |
| | G x E | G | 0.132 | 0.010 | 0.000 | 0.092 | 0.022 | | | 0.156 | 0.019 | | | 0.144 | 0.019 | | | 0.127 | 0.028 | | | 0.128 | 0.029 | | |
| | | E | 0.041 | 0.011 | 0.000 | 0.044 | 0.027 | | | 0.045 | 0.027 | | | 0.042 | 0.026 | | | 0.037 | 0.022 | | | 0.041 | 0.025 | | |
| | | GxE | 0.049 | 0.003 | 0.000 | 0.048 | 0.007 | 66.900 | 0.000 | 0.050 | 0.006 | 82.226 | 0.000 | 0.048 | 0.006 | 78.308 | 0.000 | 0.052 | 0.008 | 54.896 | 0.000 | 0.051 | 0.008 | 48.876 | 0.000 |

*Abbreviations.* E, environmental relationship matrix using full trauma principal components; SE, standard error; LRT, log-ratio test value; LRT-P, log-ratio test P-Value; G, genetic (Genomic Relationship Matrix); E, environmental (full trauma exposure ERM); GxE, genome-by-trauma exposure interaction.

© 2022 Chuong M et al. *JAMA Psychiatry.*

**eTable 37.** Mixed Linear Model Results Including Es of Adult Trauma Exposure Principal Components

| Trait | Model | Source | META ANALYSIS | | | NORTH | | | | MIDNORTH | | | | MIDSOUTH | | | | SOUTHWEST | | | | SOUTHEAST | | | |
|---|---|---|---|---|---|---|---|---|---|---|---|---|---|---|---|---|---|---|---|---|---|---|---|---|---|
| | | | Variance | SE | P-Value | Variance | SE | LRT | LRT-P | Variance | SE | LRT | LRT-P | Variance | SE | LRT | LRT-P | Variance | SE | LRT | LRT-P | Variance | SE | LRT | LRT-P |
| CIDI | G | G | 0.167 | 0.016 | 0.000 | 0.237 | 0.043 | 33.288 | 0.000 | 0.152 | 0.034 | 20.905 | 0.000 | 0.139 | 0.034 | 17.463 | 0.000 | 0.248 | 0.054 | 21.519 | 0.000 | 0.139 | 0.031 | 21.132 | 0.000 |
| | E | E | 0.113 | 0.030 | 0.000 | 0.111 | 0.066 | 1217.822 | 0.000 | 0.123 | 0.072 | 1667.859 | 0.000 | 0.112 | 0.066 | 1476.666 | 0.000 | 0.103 | 0.062 | 845.731 | 0.000 | 0.118 | 0.070 | 1872.734 | 0.000 |
| | G + E | G | 0.132 | 0.016 | 0.000 | 0.206 | 0.041 | | | 0.122 | 0.032 | | | 0.101 | 0.032 | | | 0.217 | 0.052 | | | 0.101 | 0.029 | | |
| | | E | 0.112 | 0.030 | 0.000 | 0.110 | 0.066 | 1212.851 | 0.000 | 0.122 | 0.072 | 1662.461 | 0.000 | 0.111 | 0.066 | 1469.484 | 0.000 | 0.103 | 0.062 | 842.647 | 0.000 | 0.118 | 0.070 | 1864.260 | 0.000 |
| | G x E | G | 0.129 | 0.015 | 0.000 | 0.203 | 0.040 | | | 0.122 | 0.032 | | | 0.100 | 0.032 | | | 0.208 | 0.051 | | | 0.097 | 0.028 | | |
| | | E | 0.114 | 0.030 | 0.000 | 0.113 | 0.067 | | | 0.125 | 0.073 | | | 0.115 | 0.068 | | | 0.104 | 0.062 | | | 0.120 | 0.071 | | |
| | | GxE | 0.081 | 0.005 | 0.000 | 0.088 | 0.013 | 59.266 | 0.000 | 0.056 | 0.011 | 31.401 | 0.000 | 0.066 | 0.011 | 44.934 | 0.000 | 0.075 | 0.014 | 29.602 | 0.000 | 0.142 | 0.014 | 149.724 | 0.000 |
| BROAD | G | G | 0.139 | 0.014 | 0.000 | 0.167 | 0.035 | 23.626 | 0.000 | 0.153 | 0.028 | 31.392 | 0.000 | 0.118 | 0.029 | 17.801 | 0.000 | 0.157 | 0.046 | 11.612 | 0.000 | 0.125 | 0.025 | 25.795 | 0.000 |
| | E | E | 0.069 | 0.019 | 0.000 | 0.068 | 0.042 | 911.063 | 0.000 | 0.071 | 0.043 | 1140.854 | 0.000 | 0.065 | 0.040 | 1025.708 | 0.000 | 0.069 | 0.042 | 669.480 | 0.000 | 0.070 | 0.043 | 1351.899 | 0.000 |
| | G + E | G | 0.115 | 0.013 | 0.000 | 0.144 | 0.034 | | | 0.141 | 0.027 | | | 0.095 | 0.028 | | | 0.138 | 0.045 | | | 0.090 | 0.024 | | |
| | | E | 0.068 | 0.019 | 0.000 | 0.068 | 0.041 | 906.831 | 0.000 | 0.071 | 0.043 | 1138.513 | 0.000 | 0.065 | 0.040 | 1020.536 | 0.000 | 0.069 | 0.042 | 667.662 | 0.000 | 0.070 | 0.042 | 1341.111 | 0.000 |
| | G x E | G | 0.115 | 0.013 | 0.000 | 0.142 | 0.034 | | | 0.141 | 0.027 | | | 0.096 | 0.028 | | | 0.139 | 0.045 | | | 0.090 | 0.024 | | |
| | | E | 0.069 | 0.019 | 0.000 | 0.069 | 0.042 | | | 0.071 | 0.043 | | | 0.066 | 0.040 | | | 0.069 | 0.042 | | | 0.071 | 0.043 | | |
| | | GxE | 0.021 | 0.004 | 0.000 | 0.028 | 0.009 | 10.439 | 0.001 | 0.002 | 0.007 | 0.056 | 0.407 | 0.015 | 0.008 | 3.822 | 0.025 | 0.026 | 0.011 | 5.972 | 0.007 | 0.042 | 0.009 | 24.719 | 0.000 |
| NEURO | G | G | 0.154 | 0.009 | 0.000 | 0.098 | 0.023 | 19.166 | 0.000 | 0.175 | 0.020 | 87.683 | 0.000 | 0.162 | 0.020 | 67.484 | 0.000 | 0.147 | 0.030 | 25.455 | 0.000 | 0.165 | 0.017 | 104.280 | 0.000 |
| | E | E | 0.035 | 0.010 | 0.000 | 0.033 | 0.020 | 676.810 | 0.000 | 0.037 | 0.023 | 898.821 | 0.000 | 0.038 | 0.023 | 886.390 | 0.000 | 0.031 | 0.019 | 466.142 | 0.000 | 0.037 | 0.023 | 1194.391 | 0.000 |
| | G + E | G | 0.141 | 0.009 | 0.000 | 0.091 | 0.022 | | | 0.159 | 0.019 | | | 0.148 | 0.020 | | | 0.139 | 0.029 | | | 0.152 | 0.016 | | |
| | | E | 0.035 | 0.010 | 0.000 | 0.033 | 0.020 | 675.491 | 0.000 | 0.037 | 0.023 | 888.207 | 0.000 | 0.037 | 0.023 | 880.641 | 0.000 | 0.031 | 0.019 | 464.832 | 0.000 | 0.037 | 0.023 | 1185.494 | 0.000 |
| | G x E | G | 0.142 | 0.009 | 0.000 | 0.092 | 0.022 | | | 0.160 | 0.019 | | | 0.147 | 0.020 | | | 0.142 | 0.029 | | | 0.151 | 0.016 | | |
| | | E | 0.035 | 0.010 | 0.000 | 0.033 | 0.020 | | | 0.038 | 0.023 | | | 0.038 | 0.023 | | | 0.032 | 0.020 | | | 0.038 | 0.023 | | |
| | | GxE | 0.038 | 0.003 | 0.000 | 0.035 | 0.006 | 41.298 | 0.000 | 0.043 | 0.006 | 64.331 | 0.000 | 0.035 | 0.006 | 41.759 | 0.000 | 0.039 | 0.008 | 31.966 | 0.000 | 0.039 | 0.005 | 61.135 | 0.000 |

*Abbreviations.* E, environmental relationship matrix using full trauma principal components; SE, standard error; LRT, log-ratio test value; LRT-P, log-ratio test P-Value; G, genetic (Genomic Relationship Matrix); E, environmental (full trauma exposure ERM); GxE, genome-by-trauma exposure interaction.

**eTable 38.** Mixed Linear Model Results Including Es of Full Catastrophic Trauma Exposure Principal Components

| Trait | Model | Source | META ANALYSIS | | | NORTH | | | | MIDNORTH | | | | MIDSOUTH | | | | SOUTHWEST | | | | SOUTHEAST | | | |
|---|---|---|---|---|---|---|---|---|---|---|---|---|---|---|---|---|---|---|---|---|---|---|---|---|---|
| | | | Variance | SE | P-Value | Variance | SE | LRT | LRT-P | Variance | SE | LRT | LRT-P | Variance | SE | LRT | LRT-P | Variance | SE | LRT | LRT-P | Variance | SE | LRT | LRT-P |
| CIDI | G | G | 0.163 | 0.016 | 0.000 | 0.223 | 0.042 | 30.295 | 0.000 | 0.135 | 0.033 | 16.870 | 0.000 | 0.143 | 0.034 | 18.819 | 0.000 | 0.239 | 0.053 | 20.615 | 0.000 | 0.148 | 0.030 | 24.511 | 0.000 |
| | E | E | 0.053 | 0.013 | 0.000 | 0.052 | 0.030 | 547.825 | 0.000 | 0.056 | 0.032 | 825.414 | 0.000 | 0.054 | 0.030 | 764.793 | 0.000 | 0.052 | 0.029 | 444.673 | 0.000 | 0.051 | 0.029 | 811.485 | 0.000 |
| | G + E | G | 0.134 | 0.016 | 0.000 | 0.202 | 0.041 | | | 0.098 | 0.032 | | | 0.117 | 0.033 | | | 0.213 | 0.052 | | | 0.117 | 0.029 | | |
| | | E | 0.053 | 0.013 | 0.000 | 0.052 | 0.029 | 544.327 | 0.000 | 0.056 | 0.031 | 818.099 | 0.000 | 0.053 | 0.030 | 759.110 | 0.000 | 0.051 | 0.029 | 441.317 | 0.000 | 0.051 | 0.029 | 803.426 | 0.000 |
| | G x E | G | 0.130 | 0.016 | 0.000 | 0.203 | 0.040 | | | 0.094 | 0.032 | | | 0.116 | 0.033 | | | 0.209 | 0.052 | | | 0.110 | 0.029 | | |
| | | E | 0.053 | 0.013 | 0.000 | 0.053 | 0.030 | | | 0.056 | 0.032 | | | 0.054 | 0.030 | | | 0.051 | 0.029 | | | 0.051 | 0.029 | | |
| | | GxE | 0.074 | 0.006 | 0.000 | 0.069 | 0.014 | 24.637 | 0.000 | 0.075 | 0.012 | 39.458 | 0.000 | 0.058 | 0.012 | 24.359 | 0.000 | 0.082 | 0.016 | 27.677 | 0.000 | 0.092 | 0.013 | 53.042 | 0.000 |
| BROAD | G | G | 0.137 | 0.013 | 0.000 | 0.148 | 0.034 | 19.739 | 0.000 | 0.140 | 0.028 | 27.559 | 0.000 | 0.123 | 0.028 | 19.773 | 0.000 | 0.162 | 0.046 | 12.620 | 0.000 | 0.133 | 0.025 | 30.092 | 0.000 |
| | E | E | 0.027 | 0.007 | 0.000 | 0.030 | 0.017 | 369.925 | 0.000 | 0.029 | 0.016 | 496.765 | 0.000 | 0.023 | 0.013 | 370.424 | 0.000 | 0.026 | 0.015 | 252.145 | 0.000 | 0.029 | 0.017 | 557.863 | 0.000 |
| | G + E | G | 0.126 | 0.013 | 0.000 | 0.144 | 0.034 | | | 0.126 | 0.027 | | | 0.114 | 0.028 | | | 0.155 | 0.045 | | | 0.117 | 0.024 | | |
| | | E | 0.026 | 0.007 | 0.000 | 0.030 | 0.017 | 369.656 | 0.000 | 0.028 | 0.016 | 492.455 | 0.000 | 0.023 | 0.013 | 368.628 | 0.000 | 0.026 | 0.015 | 251.554 | 0.000 | 0.029 | 0.016 | 552.287 | 0.000 |
| | G x E | G | 0.126 | 0.013 | 0.000 | 0.142 | 0.034 | | | 0.126 | 0.027 | | | 0.114 | 0.028 | | | 0.153 | 0.045 | | | 0.117 | 0.024 | | |
| | | E | 0.026 | 0.007 | 0.000 | 0.027 | 0.015 | | | 0.028 | 0.016 | | | 0.023 | 0.013 | | | 0.026 | 0.015 | | | 0.029 | 0.016 | | |
| | | GxE | 0.008 | 0.005 | 0.090 | 0.000 | 0.010 | 0.000 | 0.500 | 0.012 | 0.009 | 1.639 | 0.100 | 0.000 | 0.009 | 0.000 | 0.494 | 0.020 | 0.013 | 2.522 | 0.056 | 0.011 | 0.010 | 1.203 | 0.136 |
| NEURO | G | G | 0.152 | 0.009 | 0.000 | 0.104 | 0.023 | 22.301 | 0.000 | 0.174 | 0.019 | 89.921 | 0.000 | 0.155 | 0.020 | 65.198 | 0.000 | 0.140 | 0.030 | 23.527 | 0.000 | 0.161 | 0.016 | 102.537 | 0.000 |
| | E | E | 0.007 | 0.002 | 0.000 | 0.007 | 0.004 | 134.114 | 0.000 | 0.010 | 0.006 | 243.103 | 0.000 | 0.006 | 0.004 | 145.426 | 0.000 | 0.007 | 0.004 | 94.148 | 0.000 | 0.007 | 0.004 | 200.821 | 0.000 |
| | G + E | G | 0.149 | 0.009 | 0.000 | 0.106 | 0.022 | | | 0.168 | 0.019 | | | 0.158 | 0.020 | | | 0.135 | 0.029 | | | 0.156 | 0.016 | | |
| | | E | 0.007 | 0.002 | 0.000 | 0.008 | 0.004 | 135.479 | 0.000 | 0.010 | 0.006 | 238.450 | 0.000 | 0.006 | 0.004 | 149.255 | 0.000 | 0.007 | 0.004 | 92.922 | 0.000 | 0.007 | 0.004 | 195.251 | 0.000 |
| | G x E | G | 0.149 | 0.009 | 0.000 | 0.105 | 0.022 | | | 0.168 | 0.019 | | | 0.160 | 0.020 | | | 0.136 | 0.029 | | | 0.156 | 0.016 | | |
| | | E | 0.007 | 0.002 | 0.000 | 0.008 | 0.005 | | | 0.010 | 0.006 | | | 0.006 | 0.004 | | | 0.007 | 0.004 | | | 0.007 | 0.004 | | |
| | | GxE | 0.010 | 0.003 | 0.000 | 0.006 | 0.007 | 0.747 | 0.194 | 0.016 | 0.007 | 5.852 | 0.008 | 0.013 | 0.006 | 3.997 | 0.023 | 0.015 | 0.008 | 3.502 | 0.031 | 0.005 | 0.006 | 0.854 | 0.178 |

*Abbreviations.* E, environmental relationship matrix using full trauma principal components; SE, standard error; LRT, log-ratio test value; LRT-P, log-ratio test P-Value; G, genetic (Genomic Relationship Matrix); E, environmental (full trauma exposure ERM); GxE, genome-by-trauma exposure interaction.

© 2022 Chuong M et al. *JAMA Psychiatry.*

**eTable 39.** Mixed Linear Model Results Including Es of Full Trauma Exposure Principal Components Using Unrelated Individuals

| Trait | Model | Source | META ANALYSIS | | | NORTH | | | | MIDNORTH | | | | MIDSOUTH | | | | SOUTHWEST | | | | SOUTHEAST | | | |
|---|---|---|---|---|---|---|---|---|---|---|---|---|---|---|---|---|---|---|---|---|---|---|---|---|---|
| | | | Variance | SE | P-Value | Variance | SE | LRT | LRT-P | Variance | SE | LRT | LRT-P | Variance | SE | LRT | LRT-P | Variance | SE | LRT | LRT-P | Variance | SE | LRT | LRT-P |
| CIDI | G | G | 0.154 | 0.019 | 0.000 | 0.207 | 0.052 | 16.837 | 0.000 | 0.155 | 0.040 | 15.213 | 0.000 | 0.146 | 0.040 | 14.014 | 0.000 | 0.212 | 0.064 | 11.228 | 0.000 | 0.118 | 0.034 | 12.292 | 0.000 |
| | E | E | 0.148 | 0.021 | 0.000 | 0.142 | 0.046 | 1539.533 | 0.000 | 0.161 | 0.051 | 2282.157 | 0.000 | 0.148 | 0.047 | 2070.893 | 0.000 | 0.137 | 0.045 | 1218.759 | 0.000 | 0.160 | 0.051 | 2751.651 | 0.000 |
| | G + E | G | 0.094 | 0.017 | 0.000 | 0.159 | 0.047 | | | 0.098 | 0.036 | | | 0.087 | 0.036 | | | 0.137 | 0.058 | | | 0.056 | 0.031 | | |
| | | E | 0.174 | 0.025 | 0.000 | 0.167 | 0.054 | 1534.810 | 0.000 | 0.188 | 0.060 | 2274.722 | 0.000 | 0.173 | 0.055 | 2062.838 | 0.000 | 0.161 | 0.052 | 1213.166 | 0.000 | 0.188 | 0.060 | 2742.841 | 0.000 |
| | G x E | G | 0.089 | 0.017 | 0.000 | 0.164 | 0.046 | | | 0.098 | 0.035 | | | 0.085 | 0.036 | | | 0.128 | 0.057 | | | 0.043 | 0.029 | | |
| | | E | 0.177 | 0.025 | 0.000 | 0.172 | 0.056 | | | 0.192 | 0.061 | | | 0.177 | 0.056 | | | 0.161 | 0.053 | | | 0.189 | 0.061 | | |
| | | GxE | 0.199 | 0.009 | 0.000 | 0.243 | 0.023 | 139.913 | 0.000 | 0.161 | 0.018 | 90.888 | 0.000 | 0.148 | 0.018 | 81.912 | 0.000 | 0.182 | 0.024 | 64.257 | 0.000 | 0.299 | 0.021 | 285.257 | 0.000 |
| BROAD | G | G | 0.119 | 0.015 | 0.000 | 0.159 | 0.043 | 14.241 | 0.000 | 0.146 | 0.033 | 20.110 | 0.000 | 0.122 | 0.034 | 13.515 | 0.000 | 0.123 | 0.044 | 8.017 | 0.002 | 0.093 | 0.023 | 16.805 | 0.000 |
| | E | E | 0.086 | 0.013 | 0.000 | 0.083 | 0.028 | 1113.781 | 0.000 | 0.086 | 0.029 | 1478.493 | 0.000 | 0.083 | 0.028 | 1396.299 | 0.000 | 0.087 | 0.029 | 921.338 | 0.000 | 0.091 | 0.030 | 1960.973 | 0.000 |
| | G + E | G | 0.097 | 0.015 | 0.000 | 0.137 | 0.040 | | | 0.114 | 0.031 | | | 0.085 | 0.032 | | | 0.134 | 0.051 | | | 0.068 | 0.026 | | |
| | | E | 0.106 | 0.016 | 0.000 | 0.103 | 0.035 | 1111.684 | 0.000 | 0.106 | 0.036 | 1472.698 | 0.000 | 0.102 | 0.034 | 1390.426 | 0.000 | 0.107 | 0.036 | 920.553 | 0.000 | 0.112 | 0.037 | 1951.169 | 0.000 |
| | G x E | G | 0.097 | 0.015 | 0.000 | 0.136 | 0.040 | | | 0.115 | 0.031 | | | 0.085 | 0.032 | | | 0.132 | 0.050 | | | 0.068 | 0.026 | | |
| | | E | 0.107 | 0.016 | 0.000 | 0.104 | 0.035 | | | 0.107 | 0.036 | | | 0.102 | 0.034 | | | 0.108 | 0.036 | | | 0.113 | 0.038 | | |
| | | GxE | 0.049 | 0.007 | 0.000 | 0.053 | 0.016 | 11.372 | 0.000 | 0.025 | 0.013 | 3.156 | 0.038 | 0.025 | 0.014 | 3.204 | 0.037 | 0.070 | 0.019 | 12.984 | 0.000 | 0.091 | 0.014 | 41.230 | 0.000 |
| NEURO | G | G | 0.138 | 0.011 | 0.000 | 0.081 | 0.028 | 8.975 | 0.001 | 0.155 | 0.023 | 49.616 | 0.000 | 0.142 | 0.023 | 38.894 | 0.000 | 0.132 | 0.035 | 14.886 | 0.000 | 0.152 | 0.019 | 70.069 | 0.000 |
| | E | E | 0.059 | 0.009 | 0.000 | 0.059 | 0.020 | 1016.525 | 0.000 | 0.066 | 0.022 | 1364.508 | 0.000 | 0.063 | 0.021 | 1294.966 | 0.000 | 0.051 | 0.017 | 667.636 | 0.000 | 0.062 | 0.021 | 1776.746 | 0.000 |
| | G + E | G | 0.122 | 0.010 | 0.000 | 0.073 | 0.026 | | | 0.127 | 0.021 | | | 0.126 | 0.022 | | | 0.125 | 0.033 | | | 0.138 | 0.018 | | |
| | | E | 0.059 | 0.009 | 0.000 | 0.059 | 0.020 | 1015.899 | 0.000 | 0.065 | 0.022 | 1352.817 | 0.000 | 0.063 | 0.021 | 1291.776 | 0.000 | 0.051 | 0.017 | 667.619 | 0.000 | 0.062 | 0.021 | 1771.728 | 0.000 |
| | G x E | G | 0.123 | 0.010 | 0.000 | 0.072 | 0.026 | | | 0.129 | 0.021 | | | 0.129 | 0.022 | | | 0.131 | 0.033 | | | 0.136 | 0.018 | | |
| | | E | 0.060 | 0.009 | 0.000 | 0.060 | 0.020 | | | 0.067 | 0.022 | | | 0.064 | 0.021 | | | 0.052 | 0.018 | | | 0.063 | 0.021 | | |
| | | GxE | 0.074 | 0.004 | 0.000 | 0.065 | 0.010 | 48.482 | 0.000 | 0.076 | 0.010 | 72.565 | 0.000 | 0.073 | 0.010 | 68.513 | 0.000 | 0.076 | 0.012 | 45.650 | 0.000 | 0.077 | 0.009 | 88.384 | 0.000 |

*Abbreviations.* ERM, environmental relationship matrix using full trauma principal components; SE, standard error; LRT, log-ratio test value; LRT-P, log-ratio test P-Value; G, genetic (Genomic Relationship Matrix); E, environmental (full trauma exposure ERM); GxE, genome-by-trauma exposure interaction.

© 2022 Chuong M et al. *JAMA Psychiatry.*

**eTable 40.** Mixed Linear Model Results Including Es of Full Trauma Exposure Principal Components Precorrected for Genomic Relationship Matrix

| Trait | Model | Source | META ANALYSIS | | | NORTH | | | | MIDNORTH | | | | MIDSOUTH | | | | SOUTHWEST | | | | SOUTHEAST | | | |
|---|---|---|---|---|---|---|---|---|---|---|---|---|---|---|---|---|---|---|---|---|---|---|---|---|---|
| | | | Variance | SE | P-Value | Variance | SE | LRT | LRT-P | Variance | SE | LRT | LRT-P | Variance | SE | LRT | LRT-P | Variance | SE | LRT | LRT-P | Variance | SE | LRT | LRT-P |
| CIDI | G | G | 0.167 | 0.017 | 0.000 | 0.229 | 0.044 | 28.956 | 0.000 | 0.158 | 0.035 | 20.793 | 0.000 | 0.134 | 0.035 | 15.209 | 0.000 | 0.256 | 0.056 | 21.359 | 0.000 | 0.142 | 0.032 | 20.707 | 0.000 |
| | E | E | 0.170 | 0.024 | 0.000 | 0.165 | 0.053 | 1776.693 | 0.000 | 0.180 | 0.057 | 2490.465 | 0.000 | 0.167 | 0.054 | 2269.990 | 0.000 | 0.160 | 0.052 | 1369.547 | 0.000 | 0.180 | 0.058 | 2914.130 | 0.000 |
| | G + E | G | 0.099 | 0.014 | 0.000 | 0.179 | 0.040 | | | 0.087 | 0.027 | | | 0.074 | 0.027 | | | 0.170 | 0.051 | | | 0.078 | 0.028 | | |
| | | E | 0.157 | 0.023 | 0.000 | 0.163 | 0.053 | 1769.053 | 0.000 | 0.152 | 0.049 | 2480.528 | 0.000 | 0.141 | 0.045 | 2262.515 | 0.000 | 0.159 | 0.052 | 1359.598 | 0.000 | 0.179 | 0.058 | 2901.274 | 0.000 |
| | G x E | G | 0.103 | 0.015 | 0.000 | 0.182 | 0.039 | | | 0.101 | 0.031 | | | 0.082 | 0.031 | | | 0.162 | 0.050 | | | 0.066 | 0.027 | | |
| | | E | 0.171 | 0.025 | 0.000 | 0.169 | 0.055 | | | 0.182 | 0.058 | | | 0.169 | 0.054 | | | 0.159 | 0.052 | | | 0.179 | 0.058 | | |
| | | GxE | 0.205 | 0.009 | 0.000 | 0.243 | 0.021 | 158.523 | 0.000 | 0.168 | 0.018 | 107.173 | 0.000 | 0.152 | 0.017 | 92.227 | 0.000 | 0.201 | 0.023 | 83.884 | 0.000 | 0.300 | 0.021 | 300.562 | 0.000 |
| BROAD | G | G | 0.135 | 0.014 | 0.000 | 0.154 | 0.036 | 19.061 | 0.000 | 0.160 | 0.029 | 32.247 | 0.000 | 0.110 | 0.030 | 14.595 | 0.000 | 0.159 | 0.048 | 11.076 | 0.000 | 0.118 | 0.026 | 21.365 | 0.000 |
| | E | E | 0.102 | 0.015 | 0.000 | 0.101 | 0.034 | 1286.139 | 0.000 | 0.102 | 0.034 | 1635.383 | 0.000 | 0.097 | 0.032 | 1517.333 | 0.000 | 0.104 | 0.035 | 1013.179 | 0.000 | 0.105 | 0.035 | 2054.566 | 0.000 |
| | G + E | G | 0.088 | 0.012 | 0.000 | 0.124 | 0.034 | | | 0.104 | 0.022 | | | 0.061 | 0.022 | | | 0.118 | 0.045 | | | 0.072 | 0.024 | | |
| | | E | 0.091 | 0.014 | 0.000 | 0.100 | 0.034 | 1281.304 | 0.000 | 0.082 | 0.027 | 1626.904 | 0.000 | 0.078 | 0.026 | 1510.685 | 0.000 | 0.103 | 0.035 | 1009.162 | 0.000 | 0.105 | 0.035 | 2042.577 | 0.000 |
| | G x E | G | 0.098 | 0.013 | 0.000 | 0.123 | 0.034 | | | 0.128 | 0.027 | | | 0.076 | 0.028 | | | 0.116 | 0.045 | | | 0.072 | 0.024 | | |
| | | E | 0.102 | 0.015 | 0.000 | 0.101 | 0.034 | | | 0.102 | 0.034 | | | 0.096 | 0.032 | | | 0.104 | 0.035 | | | 0.106 | 0.035 | | |
| | | GxE | 0.050 | 0.006 | 0.000 | 0.052 | 0.015 | 12.608 | 0.000 | 0.028 | 0.013 | 4.625 | 0.016 | 0.022 | 0.013 | 2.913 | 0.044 | 0.075 | 0.018 | 16.759 | 0.000 | 0.090 | 0.014 | 42.857 | 0.000 |
| NEURO | G | G | 0.150 | 0.009 | 0.000 | 0.102 | 0.024 | 19.868 | 0.000 | 0.174 | 0.020 | 81.415 | 0.000 | 0.147 | 0.021 | 54.107 | 0.000 | 0.148 | 0.031 | 24.168 | 0.000 | 0.160 | 0.017 | 94.024 | 0.000 |
| | E | E | 0.058 | 0.009 | 0.000 | 0.059 | 0.020 | 1213.783 | 0.000 | 0.063 | 0.021 | 1518.197 | 0.000 | 0.059 | 0.020 | 1415.037 | 0.000 | 0.052 | 0.018 | 784.201 | 0.000 | 0.059 | 0.020 | 1884.675 | 0.000 |
| | G + E | G | 0.132 | 0.009 | 0.000 | 0.093 | 0.022 | | | 0.147 | 0.019 | | | 0.131 | 0.020 | | | 0.130 | 0.029 | | | 0.145 | 0.016 | | |
| | | E | 0.058 | 0.009 | 0.000 | 0.059 | 0.020 | 1212.593 | 0.000 | 0.062 | 0.021 | 1502.818 | 0.000 | 0.059 | 0.020 | 1410.217 | 0.000 | 0.052 | 0.018 | 780.807 | 0.000 | 0.058 | 0.020 | 1877.945 | 0.000 |
| | G x E | G | 0.133 | 0.009 | 0.000 | 0.092 | 0.022 | | | 0.150 | 0.019 | | | 0.133 | 0.019 | | | 0.132 | 0.029 | | | 0.144 | 0.016 | | |
| | | E | 0.059 | 0.009 | 0.000 | 0.060 | 0.020 | | | 0.064 | 0.021 | | | 0.060 | 0.020 | | | 0.053 | 0.018 | | | 0.059 | 0.020 | | |
| | | GxE | 0.073 | 0.004 | 0.000 | 0.063 | 0.010 | 51.355 | 0.000 | 0.076 | 0.009 | 80.835 | 0.000 | 0.074 | 0.009 | 75.554 | 0.000 | 0.081 | 0.012 | 55.533 | 0.000 | 0.073 | 0.008 | 86.113 | 0.000 |

*Abbreviations.* E, environmental relationship matrix using full trauma principal components; SE, standard error; LRT, log-ratio test value; LRT-P, log-ratio test P-Value; G, genetic (Genomic Relationship Matrix); E, environmental (full trauma exposure ERM); GxE, genome-by-trauma exposure interaction.

© 2022 Chuong M et al. *JAMA Psychiatry.*

**eTable 41.** Mixed Linear Model Results Including Es of Full Trauma Exposure Principal Component 1

| Trait | Model | Source | META ANALYSIS | | | NORTH | | | | MIDNORTH | | | | MIDSOUTH | | | | SOUTHWEST | | | | SOUTHEAST | | | |
|---|---|---|---|---|---|---|---|---|---|---|---|---|---|---|---|---|---|---|---|---|---|---|---|---|---|
| | | | Variance | SE | P-Value | Variance | SE | LRT | LRT-P | Variance | SE | LRT | LRT-P | Variance | SE | LRT | LRT-P | Variance | SE | LRT | LRT-P | Variance | SE | LRT | LRT-P |
| CIDI | G | G | 0.167 | 0.017 | 0.000 | 0.229 | 0.044 | 28.956 | 0.000 | 0.158 | 0.035 | 20.793 | 0.000 | 0.134 | 0.035 | 15.209 | 0.000 | 0.256 | 0.056 | 21.359 | 0.000 | 0.142 | 0.032 | 20.707 | 0.000 |
| | E | E | 0.146 | 0.085 | 0.086 | 0.140 | 0.183 | 1512.750 | 0.000 | 0.148 | 0.192 | 2107.698 | 0.000 | 0.138 | 0.180 | 1884.967 | 0.000 | 0.143 | 0.187 | 1223.926 | 0.000 | 0.166 | 0.214 | 2536.516 | 0.000 |
| | G + E | G | 0.096 | 0.015 | 0.000 | 0.140 | 0.038 | | | 0.098 | 0.034 | | | 0.081 | 0.034 | | | 0.147 | 0.047 | | | 0.068 | 0.026 | | |
| | | E | 0.131 | 0.077 | 0.086 | 0.118 | 0.155 | 1501.103 | 0.000 | 0.148 | 0.192 | 2096.645 | 0.000 | 0.138 | 0.179 | 1876.219 | 0.000 | 0.121 | 0.158 | 1214.063 | 0.000 | 0.141 | 0.182 | 2523.859 | 0.000 |
| | G x E | G | 0.103 | 0.016 | 0.000 | 0.164 | 0.044 | | | 0.098 | 0.034 | | | 0.079 | 0.034 | | | 0.173 | 0.055 | | | 0.079 | 0.030 | | |
| | | E | 0.151 | 0.088 | 0.085 | 0.152 | 0.197 | | | 0.150 | 0.194 | | | 0.144 | 0.186 | | | 0.143 | 0.186 | | | 0.174 | 0.224 | | |
| | | GxE | 0.014 | 0.003 | 0.000 | 0.040 | 0.010 | 31.069 | 0.000 | 0.004 | 0.005 | 0.937 | 0.166 | 0.016 | 0.006 | 8.815 | 0.001 | 0.003 | 0.008 | 0.115 | 0.367 | 0.043 | 0.010 | 40.008 | 0.000 |
| BROAD | G | G | 0.135 | 0.014 | 0.000 | 0.154 | 0.036 | 19.061 | 0.000 | 0.160 | 0.029 | 32.247 | 0.000 | 0.110 | 0.030 | 14.595 | 0.000 | 0.159 | 0.048 | 11.076 | 0.000 | 0.118 | 0.026 | 21.365 | 0.000 |
| | E | E | 0.088 | 0.053 | 0.096 | 0.086 | 0.116 | 1112.191 | 0.000 | 0.087 | 0.116 | 1418.563 | 0.000 | 0.081 | 0.109 | 1286.448 | 0.000 | 0.092 | 0.123 | 898.291 | 0.000 | 0.097 | 0.129 | 1794.423 | 0.000 |
| | G + E | G | 0.089 | 0.012 | 0.000 | 0.096 | 0.028 | | | 0.130 | 0.029 | | | 0.081 | 0.028 | | | 0.105 | 0.037 | | | 0.066 | 0.020 | | |
| | | E | 0.077 | 0.046 | 0.096 | 0.070 | 0.093 | 1105.984 | 0.000 | 0.086 | 0.116 | 1410.377 | 0.000 | 0.081 | 0.109 | 1280.978 | 0.000 | 0.074 | 0.099 | 895.655 | 0.000 | 0.078 | 0.104 | 1785.255 | 0.000 |
| | G x E | G | 0.100 | 0.014 | 0.000 | 0.118 | 0.035 | | | 0.125 | 0.029 | | | 0.078 | 0.028 | | | 0.126 | 0.046 | | | 0.081 | 0.025 | | |
| | | E | 0.083 | 0.049 | 0.089 | 0.089 | 0.119 | | | 0.078 | 0.099 | | | 0.076 | 0.099 | | | 0.085 | 0.110 | | | 0.096 | 0.129 | | |
| | | GxE | 0.002 | 0.002 | 0.471 | 0.010 | 0.006 | 3.457 | 0.031 | 0.000 | 0.005 | 0.000 | 0.500 | 0.000 | 0.005 | 0.000 | 0.500 | 0.000 | 0.007 | 0.000 | 0.500 | 0.000 | 0.005 | 0.001 | 0.488 |
| NEURO | G | G | 0.150 | 0.009 | 0.000 | 0.102 | 0.024 | 19.868 | 0.000 | 0.174 | 0.020 | 81.415 | 0.000 | 0.147 | 0.021 | 54.107 | 0.000 | 0.148 | 0.031 | 24.168 | 0.000 | 0.160 | 0.017 | 94.024 | 0.000 |
| | E | E | 0.039 | 0.024 | 0.100 | 0.039 | 0.053 | 813.621 | 0.000 | 0.041 | 0.056 | 1002.493 | 0.000 | 0.040 | 0.055 | 969.691 | 0.000 | 0.038 | 0.051 | 575.482 | 0.000 | 0.040 | 0.054 | 1199.364 | 0.000 |
| | G + E | G | 0.136 | 0.010 | 0.000 | 0.096 | 0.023 | | | 0.156 | 0.021 | | | 0.142 | 0.021 | | | 0.131 | 0.030 | | | 0.142 | 0.018 | | |
| | | E | 0.039 | 0.024 | 0.100 | 0.039 | 0.053 | 812.995 | 0.000 | 0.041 | 0.055 | 992.386 | 0.000 | 0.040 | 0.055 | 971.070 | 0.000 | 0.037 | 0.051 | 572.233 | 0.000 | 0.039 | 0.054 | 1186.482 | 0.000 |
| | G x E | G | 0.134 | 0.010 | 0.000 | 0.092 | 0.023 | | | 0.155 | 0.022 | | | 0.142 | 0.021 | | | 0.132 | 0.030 | | | 0.139 | 0.018 | | |
| | | E | 0.043 | 0.026 | 0.099 | 0.044 | 0.060 | | | 0.046 | 0.062 | | | 0.043 | 0.059 | | | 0.040 | 0.055 | | | 0.044 | 0.059 | | |
| | | GxE | 0.025 | 0.002 | 0.000 | 0.027 | 0.005 | 51.053 | 0.000 | 0.027 | 0.005 | 60.822 | 0.000 | 0.020 | 0.004 | 31.918 | 0.000 | 0.026 | 0.006 | 26.177 | 0.000 | 0.026 | 0.004 | 58.352 | 0.000 |

*Abbreviations.* E, environmental relationship matrix using full trauma principal components; SE, standard error; LRT, log-ratio test value; LRT-P, log-ratio test P-Value; G, genetic (Genomic Relationship Matrix); E, environmental (full trauma exposure ERM); GxE, genome-by-trauma exposure interaction.

© 2022 Chuong M et al. *JAMA Psychiatry.*

**eTable 42.** Mixed Linear Model Results Including Es of Female Only Full Trauma Exposure Principal Components

| Trait | Model | Source | META ANALYSIS | | | NORTH | | | | MIDNORTH | | | | MIDSOUTH | | | | SOUTHWEST | | | | SOUTHEAST | | | |
|---|---|---|---|---|---|---|---|---|---|---|---|---|---|---|---|---|---|---|---|---|---|---|---|---|---|
| | | | Variance | SE | P-Value | Variance | SE | LRT | LRT-P | Variance | SE | LRT | LRT-P | Variance | SE | LRT | LRT-P | Variance | SE | LRT | LRT-P | Variance | SE | LRT | LRT-P |
| CIDI | G | G | 0.198 | 0.029 | 0.000 | 0.322 | 0.074 | 20.003 | 0.000 | 0.231 | 0.059 | 15.459 | 0.000 | 0.182 | 0.062 | 8.956 | 0.001 | 0.323 | 0.094 | 12.044 | 0.000 | 0.088 | 0.051 | 2.962 | 0.043 |
| | E | E | 0.150 | 0.022 | 0.000 | 0.153 | 0.050 | 1164.678 | 0.000 | 0.155 | 0.050 | 1526.949 | 0.000 | 0.146 | 0.048 | 1371.836 | 0.000 | 0.140 | 0.046 | 840.290 | 0.000 | 0.156 | 0.051 | 1820.246 | 0.000 |
| | G + E | G | 0.125 | 0.026 | 0.000 | 0.265 | 0.068 | | | 0.161 | 0.054 | | | 0.114 | 0.056 | | | 0.223 | 0.086 | | | 0.015 | 0.046 | | |
| | | E | 0.149 | 0.022 | 0.000 | 0.153 | 0.050 | 1160.699 | 0.000 | 0.154 | 0.050 | 1520.678 | 0.000 | 0.146 | 0.047 | 1367.036 | 0.000 | 0.139 | 0.046 | 834.981 | 0.000 | 0.156 | 0.051 | 1817.387 | 0.000 |
| | G x E | G | 0.123 | 0.026 | 0.000 | 0.270 | 0.068 | | | 0.164 | 0.054 | | | 0.112 | 0.056 | | | 0.220 | 0.086 | | | 0.006 | 0.046 | | |
| | | E | 0.152 | 0.022 | 0.000 | 0.158 | 0.052 | | | 0.156 | 0.050 | | | 0.148 | 0.048 | | | 0.141 | 0.047 | | | 0.158 | 0.051 | | |
| | | GxE | 0.086 | 0.009 | 0.000 | 0.142 | 0.023 | 40.514 | 0.000 | 0.044 | 0.017 | 5.911 | 0.008 | 0.043 | 0.017 | 6.259 | 0.006 | 0.098 | 0.025 | 14.488 | 0.000 | 0.154 | 0.020 | 62.105 | 0.000 |
| BROAD | G | G | 0.162 | 0.024 | 0.000 | 0.127 | 0.062 | 4.326 | 0.019 | 0.249 | 0.051 | 25.049 | 0.000 | 0.068 | 0.052 | 1.733 | 0.094 | 0.201 | 0.081 | 6.085 | 0.007 | 0.169 | 0.043 | 16.118 | 0.000 |
| | E | E | 0.098 | 0.015 | 0.000 | 0.100 | 0.034 | 893.573 | 0.000 | 0.098 | 0.033 | 1079.739 | 0.000 | 0.094 | 0.032 | 991.726 | 0.000 | 0.099 | 0.034 | 673.226 | 0.000 | 0.099 | 0.033 | 1370.310 | 0.000 |
| | G + E | G | 0.119 | 0.023 | 0.000 | 0.082 | 0.058 | | | 0.180 | 0.048 | | | 0.041 | 0.049 | | | 0.143 | 0.076 | | | 0.140 | 0.040 | | |
| | | E | 0.098 | 0.015 | 0.000 | 0.100 | 0.034 | 891.270 | 0.000 | 0.097 | 0.033 | 1069.543 | 0.000 | 0.094 | 0.032 | 990.727 | 0.000 | 0.099 | 0.034 | 670.672 | 0.000 | 0.099 | 0.033 | 1367.076 | 0.000 |
| | G x E | G | 0.113 | 0.023 | 0.000 | 0.081 | 0.058 | | | 0.167 | 0.048 | | | 0.029 | 0.049 | | | 0.143 | 0.076 | | | 0.139 | 0.040 | | |
| | | E | 0.090 | 0.013 | 0.000 | 0.100 | 0.034 | | | 0.083 | 0.026 | | | 0.079 | 0.025 | | | 0.099 | 0.034 | | | 0.099 | 0.033 | | |
| | | GxE | 0.007 | 0.007 | 0.347 | 0.008 | 0.017 | 0.221 | 0.319 | 0.000 | 0.015 | 0.000 | 0.500 | 0.000 | 0.016 | 0.000 | 0.500 | 0.005 | 0.020 | 0.056 | 0.407 | 0.020 | 0.015 | 1.549 | 0.107 |
| NEURO | G | G | 0.154 | 0.017 | 0.000 | 0.164 | 0.043 | 15.340 | 0.000 | 0.147 | 0.036 | 17.099 | 0.000 | 0.161 | 0.037 | 20.109 | 0.000 | 0.159 | 0.054 | 8.932 | 0.001 | 0.150 | 0.030 | 26.136 | 0.000 |
| | E | E | 0.051 | 0.008 | 0.000 | 0.057 | 0.019 | 681.303 | 0.000 | 0.055 | 0.019 | 792.602 | 0.000 | 0.049 | 0.017 | 689.270 | 0.000 | 0.046 | 0.016 | 422.747 | 0.000 | 0.051 | 0.017 | 1003.343 | 0.000 |
| | G + E | G | 0.142 | 0.016 | 0.000 | 0.143 | 0.040 | | | 0.115 | 0.034 | | | 0.161 | 0.035 | | | 0.142 | 0.052 | | | 0.150 | 0.028 | | |
| | | E | 0.051 | 0.008 | 0.000 | 0.056 | 0.019 | 678.919 | 0.000 | 0.055 | 0.019 | 787.164 | 0.000 | 0.049 | 0.017 | 691.742 | 0.000 | 0.046 | 0.016 | 421.722 | 0.000 | 0.051 | 0.018 | 1006.301 | 0.000 |
| | G x E | G | 0.144 | 0.016 | 0.000 | 0.140 | 0.040 | | | 0.118 | 0.034 | | | 0.164 | 0.035 | | | 0.152 | 0.051 | | | 0.148 | 0.028 | | |
| | | E | 0.052 | 0.008 | 0.000 | 0.057 | 0.020 | | | 0.056 | 0.019 | | | 0.050 | 0.017 | | | 0.047 | 0.016 | | | 0.053 | 0.018 | | |
| | | GxE | 0.061 | 0.005 | 0.000 | 0.053 | 0.012 | 24.488 | 0.000 | 0.063 | 0.011 | 37.812 | 0.000 | 0.060 | 0.011 | 32.006 | 0.000 | 0.066 | 0.014 | 23.697 | 0.000 | 0.065 | 0.010 | 45.881 | 0.000 |

*Abbreviations.* E, environmental relationship matrix using full trauma principal components; SE, standard error; LRT, log-ratio test value; LRT-P, log-ratio test P-Value; G, genetic (Genomic Relationship Matrix); E, environmental (full trauma exposure ERM); GxE, genome-by-trauma exposure interaction.

© 2022 Chuong M et al. *JAMA Psychiatry.*

**eTable 43.** Mixed Linear Model Results Including Es of Male Only Full Trauma Exposure Principal Components

| Trait | Model | Source | META ANALYSIS | | | NORTH | | | | MIDNORTH | | | | MIDSOUTH | | | | SOUTHWEST | | | | SOUTHEAST | | | |
|---|---|---|---|---|---|---|---|---|---|---|---|---|---|---|---|---|---|---|---|---|---|---|---|---|---|
| | | | Variance | SE | P-Value | Variance | SE | LRT | LRT-P | Variance | SE | LRT | LRT-P | Variance | SE | LRT | LRT-P | Variance | SE | LRT | LRT-P | Variance | SE | LRT | LRT-P |
| CIDI | G | G | 0.184 | 0.043 | 0.000 | 0.251 | 0.107 | 5.616 | 0.009 | 0.297 | 0.088 | 11.781 | 0.000 | 0.107 | 0.086 | 1.587 | 0.104 | 0.223 | 0.140 | 2.617 | 0.053 | 0.099 | 0.083 | 1.382 | 0.120 |
| | E | E | 0.247 | 0.036 | 0.000 | 0.205 | 0.068 | 502.832 | 0.000 | 0.258 | 0.081 | 872.405 | 0.000 | 0.248 | 0.078 | 829.870 | 0.000 | 0.252 | 0.082 | 462.914 | 0.000 | 0.301 | 0.094 | 1021.302 | 0.000 |
| | G + E | G | 0.143 | 0.038 | 0.000 | 0.199 | 0.097 | | | 0.240 | 0.077 | | | 0.079 | 0.075 | | | 0.118 | 0.123 | | | 0.094 | 0.072 | | |
| | | E | 0.246 | 0.035 | 0.000 | 0.205 | 0.068 | 501.577 | 0.000 | 0.258 | 0.081 | 870.559 | 0.000 | 0.248 | 0.078 | 829.409 | 0.000 | 0.251 | 0.082 | 461.228 | 0.000 | 0.302 | 0.094 | 1021.584 | 0.000 |
| | G x E | G | 0.116 | 0.035 | 0.001 | 0.187 | 0.092 | | | 0.200 | 0.073 | | | 0.058 | 0.072 | | | 0.121 | 0.118 | | | 0.056 | 0.066 | | |
| | | E | 0.235 | 0.034 | 0.000 | 0.195 | 0.066 | | | 0.255 | 0.081 | | | 0.238 | 0.076 | | | 0.237 | 0.079 | | | 0.281 | 0.089 | | |
| | | GxE | 0.441 | 0.018 | 0.000 | 0.462 | 0.040 | 163.444 | 0.000 | 0.436 | 0.038 | 189.634 | 0.000 | 0.371 | 0.035 | 147.682 | 0.000 | 0.389 | 0.045 | 95.051 | 0.000 | 0.575 | 0.043 | 296.618 | 0.000 |
| BROAD | G | G | 0.086 | 0.034 | 0.012 | 0.000 | 0.086 | 0.000 | 0.500 | 0.134 | 0.068 | 4.086 | 0.022 | 0.096 | 0.070 | 1.952 | 0.081 | 0.134 | 0.117 | 1.318 | 0.126 | 0.067 | 0.066 | 1.012 | 0.157 |
| | E | E | 0.132 | 0.020 | 0.000 | 0.128 | 0.045 | 331.562 | 0.000 | 0.130 | 0.044 | 491.207 | 0.000 | 0.126 | 0.043 | 481.217 | 0.000 | 0.132 | 0.046 | 292.452 | 0.000 | 0.149 | 0.050 | 622.781 | 0.000 |
| | G + E | G | 0.056 | 0.031 | 0.074 | 0.000 | 0.080 | | | 0.098 | 0.062 | | | 0.081 | 0.065 | | | 0.075 | 0.108 | | | 0.022 | 0.060 | | |
| | | E | 0.131 | 0.020 | 0.000 | 0.124 | 0.043 | 331.531 | 0.000 | 0.130 | 0.044 | 489.710 | 0.000 | 0.126 | 0.043 | 480.871 | 0.000 | 0.132 | 0.046 | 291.614 | 0.000 | 0.149 | 0.050 | 621.903 | 0.000 |
| | G x E | G | 0.057 | 0.031 | 0.067 | 0.000 | 0.079 | | | 0.097 | 0.061 | | | 0.078 | 0.064 | | | 0.057 | 0.106 | | | 0.033 | 0.059 | | |
| | | E | 0.130 | 0.020 | 0.000 | 0.120 | 0.042 | | | 0.132 | 0.045 | | | 0.124 | 0.043 | | | 0.132 | 0.046 | | | 0.145 | 0.049 | | |
| | | GxE | 0.165 | 0.013 | 0.000 | 0.132 | 0.029 | 24.632 | 0.000 | 0.162 | 0.026 | 43.114 | 0.000 | 0.145 | 0.026 | 32.739 | 0.000 | 0.189 | 0.035 | 32.356 | 0.000 | 0.207 | 0.027 | 64.694 | 0.000 |
| NEURO | G | G | 0.160 | 0.020 | 0.000 | 0.000 | 0.051 | 0.000 | 0.500 | 0.188 | 0.043 | 20.845 | 0.000 | 0.196 | 0.044 | 20.402 | 0.000 | 0.232 | 0.068 | 12.041 | 0.000 | 0.179 | 0.037 | 23.435 | 0.000 |
| | E | E | 0.092 | 0.014 | 0.000 | 0.082 | 0.028 | 514.331 | 0.000 | 0.105 | 0.034 | 749.093 | 0.000 | 0.102 | 0.033 | 752.985 | 0.000 | 0.087 | 0.029 | 364.535 | 0.000 | 0.093 | 0.030 | 915.529 | 0.000 |
| | G + E | G | 0.135 | 0.019 | 0.000 | 0.000 | 0.047 | | | 0.166 | 0.039 | | | 0.168 | 0.040 | | | 0.196 | 0.063 | | | 0.140 | 0.034 | | |
| | | E | 0.087 | 0.013 | 0.000 | 0.070 | 0.022 | 513.589 | 0.000 | 0.105 | 0.034 | 748.589 | 0.000 | 0.102 | 0.033 | 751.243 | 0.000 | 0.086 | 0.029 | 362.562 | 0.000 | 0.092 | 0.030 | 909.666 | 0.000 |
| | G x E | G | 0.135 | 0.018 | 0.000 | 0.000 | 0.046 | | | 0.169 | 0.038 | | | 0.165 | 0.039 | | | 0.193 | 0.062 | | | 0.142 | 0.034 | | |
| | | E | 0.090 | 0.013 | 0.000 | 0.076 | 0.025 | | | 0.107 | 0.035 | | | 0.102 | 0.033 | | | 0.084 | 0.029 | | | 0.093 | 0.031 | | |
| | | GxE | 0.080 | 0.007 | 0.000 | 0.068 | 0.016 | 25.530 | 0.000 | 0.082 | 0.015 | 34.015 | 0.000 | 0.084 | 0.015 | 38.918 | 0.000 | 0.093 | 0.019 | 28.750 | 0.000 | 0.077 | 0.014 | 33.111 | 0.000 |

*Abbreviations.* E, environmental relationship matrix using full trauma principal components; SE, standard error; LRT, log-ratio test value; LRT-P, log-ratio test P-Value; G, genetic (Genomic Relationship Matrix); E, environmental (full trauma exposure ERM); GxE, genome-by-trauma exposure interaction.

© 2022 Chuong M et al. *JAMA Psychiatry.*

**eTable 44.** Mixed Linear Model Results Including Es of Female Only Full Trauma Exposure Principal Components Using Unrelated Individuals

| Trait | Model | Source | META ANALYSIS | | | NORTH | | | | MIDNORTH | | | | MIDSOUTH | | | | SOUTHWEST | | | | SOUTHEAST | | | |
|---|---|---|---|---|---|---|---|---|---|---|---|---|---|---|---|---|---|---|---|---|---|---|---|---|---|
| | | | Variance | SE | P-Value | Variance | SE | LRT | LRT-P | Variance | SE | LRT | LRT-P | Variance | SE | LRT | LRT-P | Variance | SE | LRT | LRT-P | Variance | SE | LRT | LRT-P |
| CIDI | G | G | 0.175 | 0.033 | 0.000 | 0.264 | 0.088 | 9.476 | 0.001 | 0.220 | 0.068 | 10.649 | 0.001 | 0.223 | 0.071 | 10.338 | 0.001 | 0.181 | 0.107 | 2.920 | 0.044 | 0.076 | 0.057 | 1.831 | 0.088 |
| | E | E | 0.151 | 0.022 | 0.000 | 0.154 | 0.050 | 1013.563 | 0.000 | 0.159 | 0.051 | 1393.347 | 0.000 | 0.150 | 0.049 | 1240.789 | 0.000 | 0.141 | 0.047 | 757.497 | 0.000 | 0.156 | 0.051 | 1685.144 | 0.000 |
| | G + E | G | 0.103 | 0.029 | 0.000 | 0.211 | 0.081 | | | 0.155 | 0.061 | | | 0.134 | 0.064 | | | 0.102 | 0.098 | | | 0.005 | 0.051 | | |
| | | E | 0.151 | 0.022 | 0.000 | 0.154 | 0.050 | 1011.203 | 0.000 | 0.158 | 0.051 | 1389.332 | 0.000 | 0.149 | 0.048 | 1234.951 | 0.000 | 0.140 | 0.046 | 755.646 | 0.000 | 0.156 | 0.051 | 1683.321 | 0.000 |
| | G x E | G | 0.104 | 0.029 | 0.000 | 0.230 | 0.080 | | | 0.159 | 0.061 | | | 0.132 | 0.064 | | | 0.105 | 0.098 | | | 0.000 | 0.050 | | |
| | | E | 0.154 | 0.022 | 0.000 | 0.159 | 0.052 | | | 0.160 | 0.052 | | | 0.151 | 0.049 | | | 0.142 | 0.047 | | | 0.159 | 0.052 | | |
| | | GxE | 0.084 | 0.009 | 0.000 | 0.138 | 0.024 | 33.391 | 0.000 | 0.040 | 0.018 | 4.614 | 0.016 | 0.042 | 0.018 | 5.540 | 0.009 | 0.093 | 0.026 | 12.002 | 0.000 | 0.155 | 0.021 | 59.515 | 0.000 |
| BROAD | G | G | 0.165 | 0.028 | 0.000 | 0.138 | 0.074 | 3.519 | 0.030 | 0.209 | 0.059 | 13.106 | 0.000 | 0.092 | 0.061 | 2.354 | 0.062 | 0.195 | 0.093 | 4.321 | 0.019 | 0.184 | 0.048 | 15.538 | 0.000 |
| | E | E | 0.099 | 0.015 | 0.000 | 0.100 | 0.034 | 767.453 | 0.000 | 0.100 | 0.034 | 971.030 | 0.000 | 0.095 | 0.032 | 885.712 | 0.000 | 0.102 | 0.035 | 621.371 | 0.000 | 0.100 | 0.034 | 1271.425 | 0.000 |
| | G + E | G | 0.123 | 0.026 | 0.000 | 0.105 | 0.070 | | | 0.135 | 0.055 | | | 0.059 | 0.057 | | | 0.165 | 0.088 | | | 0.150 | 0.045 | | |
| | | E | 0.099 | 0.015 | 0.000 | 0.100 | 0.034 | 766.253 | 0.000 | 0.099 | 0.033 | 964.132 | 0.000 | 0.095 | 0.032 | 884.473 | 0.000 | 0.101 | 0.035 | 620.524 | 0.000 | 0.100 | 0.034 | 1268.129 | 0.000 |
| | G x E | G | 0.117 | 0.026 | 0.000 | 0.105 | 0.070 | | | 0.120 | 0.055 | | | 0.047 | 0.057 | | | 0.165 | 0.088 | | | 0.150 | 0.045 | | |
| | | E | 0.091 | 0.013 | 0.000 | 0.100 | 0.034 | | | 0.084 | 0.026 | | | 0.082 | 0.026 | | | 0.102 | 0.035 | | | 0.100 | 0.034 | | |
| | | GxE | 0.007 | 0.008 | 0.335 | 0.009 | 0.018 | 0.220 | 0.320 | 0.000 | 0.016 | 0.000 | 0.500 | 0.000 | 0.016 | 0.000 | 0.500 | 0.006 | 0.021 | 0.072 | 0.394 | 0.021 | 0.016 | 1.580 | 0.104 |
| NEURO | G | G | 0.142 | 0.019 | 0.000 | 0.152 | 0.051 | 9.364 | 0.001 | 0.119 | 0.041 | 8.698 | 0.002 | 0.164 | 0.043 | 15.792 | 0.000 | 0.137 | 0.063 | 4.891 | 0.013 | 0.140 | 0.033 | 18.760 | 0.000 |
| | E | E | 0.051 | 0.008 | 0.000 | 0.055 | 0.019 | 569.485 | 0.000 | 0.056 | 0.019 | 707.839 | 0.000 | 0.050 | 0.017 | 628.199 | 0.000 | 0.044 | 0.016 | 361.696 | 0.000 | 0.053 | 0.018 | 952.272 | 0.000 |
| | G + E | G | 0.130 | 0.018 | 0.000 | 0.124 | 0.048 | | | 0.090 | 0.039 | | | 0.162 | 0.040 | | | 0.129 | 0.060 | | | 0.141 | 0.031 | | |
| | | E | 0.051 | 0.008 | 0.000 | 0.055 | 0.019 | 566.936 | 0.000 | 0.056 | 0.019 | 704.666 | 0.000 | 0.050 | 0.017 | 629.758 | 0.000 | 0.044 | 0.016 | 361.632 | 0.000 | 0.053 | 0.018 | 954.773 | 0.000 |
| | G x E | G | 0.132 | 0.018 | 0.000 | 0.125 | 0.048 | | | 0.093 | 0.038 | | | 0.164 | 0.040 | | | 0.145 | 0.060 | | | 0.138 | 0.031 | | |
| | | E | 0.052 | 0.008 | 0.000 | 0.056 | 0.019 | | | 0.057 | 0.019 | | | 0.051 | 0.018 | | | 0.045 | 0.016 | | | 0.055 | 0.019 | | |
| | | GxE | 0.063 | 0.006 | 0.000 | 0.057 | 0.013 | 24.349 | 0.000 | 0.066 | 0.012 | 35.395 | 0.000 | 0.056 | 0.012 | 26.064 | 0.000 | 0.060 | 0.015 | 17.931 | 0.000 | 0.073 | 0.011 | 52.091 | 0.000 |

*Abbreviations.* E, environmental relationship matrix using full trauma principal components; SE, standard error; LRT, log-ratio test value; LRT-P, log-ratio test P-Value; G, genetic (Genomic Relationship Matrix); E, environmental (full trauma exposure ERM); GxE, genome-by-trauma exposure interaction.

© 2022 Chuong M et al. *JAMA Psychiatry.*

**eTable 45.** Mixed Linear Model Results Including Es of Male Only Trauma Exposure Principal Components Using Unrelated Individuals

| Trait | Model | Source | META ANALYSIS | | | NORTH | | | | MIDNORTH | | | | MIDSOUTH | | | | SOUTHWEST | | | | SOUTHEAST | | | |
|---|---|---|---|---|---|---|---|---|---|---|---|---|---|---|---|---|---|---|---|---|---|---|---|---|---|
| | | | Variance | SE | P-Value | Variance | SE | LRT | LRT-P | Variance | SE | LRT | LRT-P | Variance | SE | LRT | LRT-P | Variance | SE | LRT | LRT-P | Variance | SE | LRT | LRT-P |
| CIDI | G | G | 0.197 | 0.048 | 0.000 | 0.224 | 0.124 | 3.377 | 0.033 | 0.369 | 0.100 | 13.950 | 0.000 | 0.129 | 0.097 | 1.781 | 0.091 | 0.230 | 0.159 | 2.115 | 0.073 | 0.089 | 0.092 | 0.921 | 0.169 |
| | E | E | 0.238 | 0.035 | 0.000 | 0.186 | 0.063 | 406.679 | 0.000 | 0.261 | 0.082 | 762.730 | 0.000 | 0.241 | 0.076 | 738.421 | 0.000 | 0.247 | 0.081 | 381.466 | 0.000 | 0.306 | 0.095 | 940.638 | 0.000 |
| | G + E | G | 0.157 | 0.043 | 0.000 | 0.193 | 0.113 | | | 0.298 | 0.089 | | | 0.103 | 0.086 | | | 0.127 | 0.140 | | | 0.081 | 0.080 | | |
| | | E | 0.237 | 0.035 | 0.000 | 0.186 | 0.063 | 406.324 | 0.000 | 0.260 | 0.082 | 760.648 | 0.000 | 0.241 | 0.076 | 738.121 | 0.000 | 0.247 | 0.081 | 380.185 | 0.000 | 0.306 | 0.095 | 940.730 | 0.000 |
| | G x E | G | 0.129 | 0.040 | 0.001 | 0.162 | 0.107 | | | 0.263 | 0.084 | | | 0.096 | 0.082 | | | 0.147 | 0.135 | | | 0.034 | 0.073 | | |
| | | E | 0.227 | 0.034 | 0.000 | 0.180 | 0.062 | | | 0.255 | 0.081 | | | 0.228 | 0.074 | | | 0.231 | 0.078 | | | 0.286 | 0.091 | | |
| | | GxE | 0.436 | 0.018 | 0.000 | 0.481 | 0.043 | 148.300 | 0.000 | 0.423 | 0.039 | 162.732 | 0.000 | 0.365 | 0.036 | 131.491 | 0.000 | 0.353 | 0.046 | 71.293 | 0.000 | 0.587 | 0.044 | 286.036 | 0.000 |
| BROAD | G | G | 0.100 | 0.039 | 0.010 | 0.005 | 0.102 | 0.002 | 0.481 | 0.146 | 0.078 | 3.661 | 0.028 | 0.125 | 0.081 | 2.503 | 0.057 | 0.219 | 0.133 | 2.763 | 0.048 | 0.054 | 0.072 | 0.553 | 0.229 |
| | E | E | 0.132 | 0.020 | 0.000 | 0.122 | 0.043 | 273.926 | 0.000 | 0.131 | 0.045 | 428.852 | 0.000 | 0.131 | 0.045 | 452.356 | 0.000 | 0.125 | 0.044 | 246.017 | 0.000 | 0.157 | 0.053 | 596.314 | 0.000 |
| | G + E | G | 0.077 | 0.036 | 0.030 | 0.019 | 0.095 | | | 0.119 | 0.072 | | | 0.118 | 0.074 | | | 0.177 | 0.124 | | | 0.010 | 0.066 | | |
| | | E | 0.132 | 0.020 | 0.000 | 0.122 | 0.043 | 273.966 | 0.000 | 0.131 | 0.044 | 428.086 | 0.000 | 0.131 | 0.045 | 452.484 | 0.000 | 0.125 | 0.044 | 245.337 | 0.000 | 0.157 | 0.053 | 595.783 | 0.000 |
| | G x E | G | 0.075 | 0.035 | 0.034 | 0.008 | 0.094 | | | 0.118 | 0.071 | | | 0.117 | 0.074 | | | 0.158 | 0.122 | | | 0.015 | 0.065 | | |
| | | E | 0.131 | 0.020 | 0.000 | 0.120 | 0.043 | | | 0.132 | 0.045 | | | 0.130 | 0.044 | | | 0.125 | 0.044 | | | 0.153 | 0.052 | | |
| | | GxE | 0.167 | 0.013 | 0.000 | 0.141 | 0.031 | 22.859 | 0.000 | 0.154 | 0.027 | 34.660 | 0.000 | 0.148 | 0.028 | 30.851 | 0.000 | 0.183 | 0.037 | 26.761 | 0.000 | 0.216 | 0.029 | 64.430 | 0.000 |
| NEURO | G | G | 0.149 | 0.023 | 0.000 | | | | | 0.192 | 0.049 | 16.366 | 0.000 | 0.180 | 0.050 | 13.491 | 0.000 | 0.169 | 0.077 | 5.099 | 0.012 | 0.162 | 0.041 | 16.004 | 0.000 |
| | E | E | 0.091 | 0.014 | 0.000 | | | | | 0.103 | 0.034 | 647.931 | 0.000 | 0.104 | 0.034 | 678.551 | 0.000 | 0.085 | 0.029 | 296.973 | 0.000 | 0.093 | 0.031 | 825.527 | 0.000 |
| | G + E | G | 0.150 | 0.023 | 0.000 | | | | | 0.174 | 0.044 | | | 0.162 | 0.045 | | | 0.156 | 0.071 | | | 0.123 | 0.037 | | |
| | | E | 0.095 | 0.016 | 0.000 | **DID NOT CONVERGE** | | | | 0.103 | 0.034 | 648.313 | 0.000 | 0.103 | 0.034 | 678.791 | 0.000 | 0.085 | 0.029 | 297.011 | 0.000 | 0.093 | 0.030 | 820.723 | 0.000 |
| | G x E | G | 0.151 | 0.023 | 0.000 | | | | | 0.175 | 0.044 | | | 0.161 | 0.044 | | | 0.156 | 0.070 | | | 0.126 | 0.037 | | |
| | | E | 0.095 | 0.016 | 0.000 | | | | | 0.105 | 0.034 | | | 0.104 | 0.034 | | | 0.083 | 0.029 | | | 0.094 | 0.031 | | |
| | | GxE | 0.084 | 0.008 | 0.000 | | | | | 0.081 | 0.016 | 29.516 | 0.000 | 0.090 | 0.016 | 39.224 | 0.000 | 0.091 | 0.020 | 25.450 | 0.000 | 0.078 | 0.014 | 31.479 | 0.000 |

*Abbreviations.* E, environmental relationship matrix using full trauma principal components; SE, standard error; LRT, log-ratio test value; LRT-P, log-ratio test P-Value; G, genetic (Genomic Relationship Matrix); E, environmental (full trauma exposure ERM); GxE, genome-by-trauma exposure interaction.

© 2022 Chuong M et al. *JAMA Psychiatry.*

**eTable 46.** Mixed Linear Model Results Including Es of Full Trauma Exposure Principal Components; Varying CIDI Depression Prevalence Rates

| Prevalence | Trait | Model | Source | NORTH | | | | MIDNORTH | | | | MIDSOUTH | | | | SOUTHWEST | | | | SOUTHEAST | | | |
|---|---|---|---|---|---|---|---|---|---|---|---|---|---|---|---|---|---|---|---|---|---|---|---|
| | | | | Variance | SE | LRT | LRT-P | Variance | SE | LRT | LRT-P | Variance | SE | LRT | LRT-P | Variance | SE | LRT | LRT-P | Variance | SE | LRT | LRT-P |
| 0.16 | CIDI | G | G | 0.195 | 0.037 | 28.956 | 0.000 | 0.134 | 0.030 | 20.793 | 0.000 | 0.114 | 0.030 | 15.209 | 0.000 | 0.218 | 0.048 | 21.359 | 0.000 | 0.121 | 0.027 | 20.707 | 0.000 |
| | | E | E | 0.145 | 0.047 | 1801.636 | 0.000 | 0.159 | 0.050 | 2528.312 | 0.000 | 0.147 | 0.047 | 2299.555 | 0.000 | 0.140 | 0.046 | 2299.555 | 0.000 | 0.159 | 0.051 | 2969.008 | 0.000 |
| | | G + E | G | 0.146 | 0.034 | | | 0.084 | 0.027 | | | 0.070 | 0.027 | | | 0.141 | 0.043 | | | 0.063 | 0.024 | | |
| | | | E | 0.144 | 0.047 | 1792.306 | 0.000 | 0.159 | 0.050 | 2517.636 | 0.000 | 0.147 | 0.047 | 2291.178 | 0.000 | 0.140 | 0.045 | 1373.706 | 0.000 | 0.159 | 0.051 | 2955.386 | 0.000 |
| | | G x E | G | 0.148 | 0.033 | | | 0.082 | 0.026 | | | 0.065 | 0.027 | | | 0.135 | 0.043 | | | 0.044 | 0.044 | | |
| | | | E | 0.150 | 0.048 | | | 0.162 | 0.051 | | | 0.150 | 0.048 | | | 0.140 | 0.045 | | | 0.163 | 0.052 | | |
| | | | GxE | 0.205 | 0.018 | 159.722 | 0.000 | 0.141 | 0.015 | 106.471 | 0.000 | 0.126 | 0.014 | 90.449 | 0.000 | 0.166 | 0.019 | 80.735 | 0.000 | 0.259 | 0.023 | 155.156 | 0.000 |
| 0.2 | CIDI | G | G | 0.208 | 0.040 | 28.956 | 0.000 | 0.144 | 0.032 | 20.793 | 0.000 | 0.122 | 0.032 | 15.209 | 0.000 | 0.234 | 0.051 | 21.359 | 0.000 | 0.129 | 0.029 | 20.707 | 0.000 |
| | | E | E | 0.155 | 0.050 | 1801.636 | 0.000 | 0.170 | 0.054 | 2528.312 | 0.000 | 0.157 | 0.050 | 2299.555 | 0.000 | 0.150 | 0.049 | 1384.156 | 0.000 | 0.171 | 0.055 | 2969.008 | 0.000 |
| | | G + E | G | 0.154 | 0.050 | | | 0.170 | 0.054 | | | 0.157 | 0.050 | | | 0.149 | 0.048 | | | 0.170 | 0.054 | | |
| | | | E | 0.156 | 0.037 | 1792.306 | | 0.090 | 0.029 | 2517.636 | 0.000 | 0.075 | 0.029 | 2291.178 | 0.000 | 0.151 | 0.046 | 1373.706 | 0.000 | 0.067 | 0.026 | 2955.386 | 0.000 |
| | | G x E | G | 0.159 | 0.036 | | | 0.087 | 0.028 | | | 0.069 | 0.029 | | | 0.144 | 0.046 | | | 0.056 | 0.025 | | |
| | | | E | 0.160 | 0.052 | | | 0.173 | 0.055 | | | 0.160 | 0.051 | | | 0.150 | 0.049 | | | 0.171 | 0.055 | | |
| | | | GxE | 0.219 | 0.019 | 159.722 | 0.000 | 0.151 | 0.411 | 106.471 | 0.000 | 0.135 | 0.015 | 90.449 | 0.000 | 0.177 | 0.021 | 80.735 | 0.000 | 0.267 | 0.019 | 295.840 | 0.000 |
| 0.28 | CIDI | G | G | 0.229 | 0.044 | 28.956 | 0.000 | 0.158 | 0.035 | 20.793 | 0.000 | 0.134 | 0.035 | 15.209 | 0.000 | 0.256 | 0.056 | 21.359 | 0.000 | 0.142 | 0.032 | 20.707 | 0.000 |
| | | E | E | 0.170 | 0.055 | 1801.636 | 0.000 | 0.187 | 0.059 | 2528.312 | 0.000 | 0.173 | 0.055 | 2299.555 | 0.000 | 0.165 | 0.053 | 1384.156 | 0.000 | 0.187 | 0.060 | 2969.008 | 0.000 |
| | | G + E | G | 0.171 | 0.040 | | | 0.098 | 0.031 | | | 0.082 | 0.032 | | | 0.166 | 0.051 | | | 0.074 | 0.028 | | |
| | | | E | 0.169 | 0.055 | 1792.306 | 0.000 | 0.186 | 0.059 | 2517.636 | 0.000 | 0.172 | 0.055 | 2291.178 | 0.000 | 0.164 | 0.053 | 1373.706 | 0.000 | 0.187 | 0.060 | 2955.386 | 0.000 |
| | | G x E | G | 0.174 | 0.039 | | | 0.096 | 0.031 | | | 0.076 | 0.031 | | | 0.158 | 0.050 | | | 0.062 | 0.027 | | |
| | | | E | 0.176 | 0.057 | | | 0.190 | 0.060 | | | 0.176 | 0.056 | | | 0.165 | 0.053 | | | 0.188 | 0.060 | | |
| | | | GxE | 0.241 | 0.021 | 159.722 | 0.000 | 0.165 | 0.017 | 106.471 | 0.000 | 0.148 | 0.017 | 90.449 | 0.000 | 0.195 | 0.023 | 80.735 | 0.000 | 0.294 | 0.020 | 295.840 | 0.000 |

*Abbreviations.* E, environmental relationship matrix using full trauma principal components; SE, standard error; LRT, log-ratio test value; LRT-P, log-ratio test P-Value; G, genetic (Genomic Relationship Matrix); E, environmental (full trauma exposure ERM); GxE, genome-by-trauma exposure interaction.

© 2022 Chuong M et al. *JAMA Psychiatry.*

**eAppendix 2.**

A consistent pattern observed in results were large LRT values when including environmental relationship matrices (Es) into the mixed linear models (see **Table SB1**). This suggests a substantial improvement in model fit when including the Es. Whilst the estimates and standard errors observed for the proportion of variance explained by the Es suggest statistical significance, they do not suggest very *strong* significance, which is discrepant with what the LRT values suggest.

**Table SB1.** North Cluster Mixed Linear Model Results Including E of Full Trauma Exposure Principal Components.

| | | | NORTH CLUSTER | | | |
|---|---|---|---|---|---|---|
| **Trait** | **Model** | **Source** | **Variance** | **SE** | **LRT** | **LRT-P** |
| CIDI | G | G | 0.229 | 0.044 | 28.956 | 0.000 |
| | E | E | 0.170 | 0.055 | 1801.636 | 0.000 |
| | G + E | G | 0.171 | 0.040 | | |
| | | E | 0.169 | 0.055 | 1792.306 | 0.000 |
| | G x E | G | 0.174 | 0.039 | | |
| | | E | 0.176 | 0.057 | | |
| | | GxE | 0.241 | 0.021 | 159.722 | 0.000 |

*Abbreviations.* CIDI, Composite International Diagnostic Interview Depression; G, Genomic Relationship Matrix; E, Environmental Relationship Matrix; GxE, Genome-by-Trauma Exposure Relationship Matrix; LRT, Log-Likelihood Ratio Test; P, P-value.

To explore this discrepancy the log-likelihood distribution was examined. A log-likelihood function aims to identify and fit the most appropriate distribution to the data available (e.g. a normal distribution). A value from the observed data will be tested as the mean of the explored distribution. A log-likelihood value, signifying the likelihood of observing the data is obtained. A range of values are tested, and the resulting log-likelihood values are plotted to form the log-likelihood distribution. The highest value i.e. the maximum likelihood estimate, signifies the optimal position of the distribution explored to the data at hand.

Here, the discrepancy suggests a potentially unusual log-likelihood distribution which may be due to the nature of the Es used. Large LRT values would suggest the distribution to have a steep increase, whereas, the smaller standard errors would suggest a plateau surrounding the maximum estimate of the log-likelihood. To plot the log-likelihood distribution, a range of E values and their corresponding log-likelihood value was plotted (**Figure SB1**).

© 2022 Chuong M et al. *JAMA Psychiatry.*

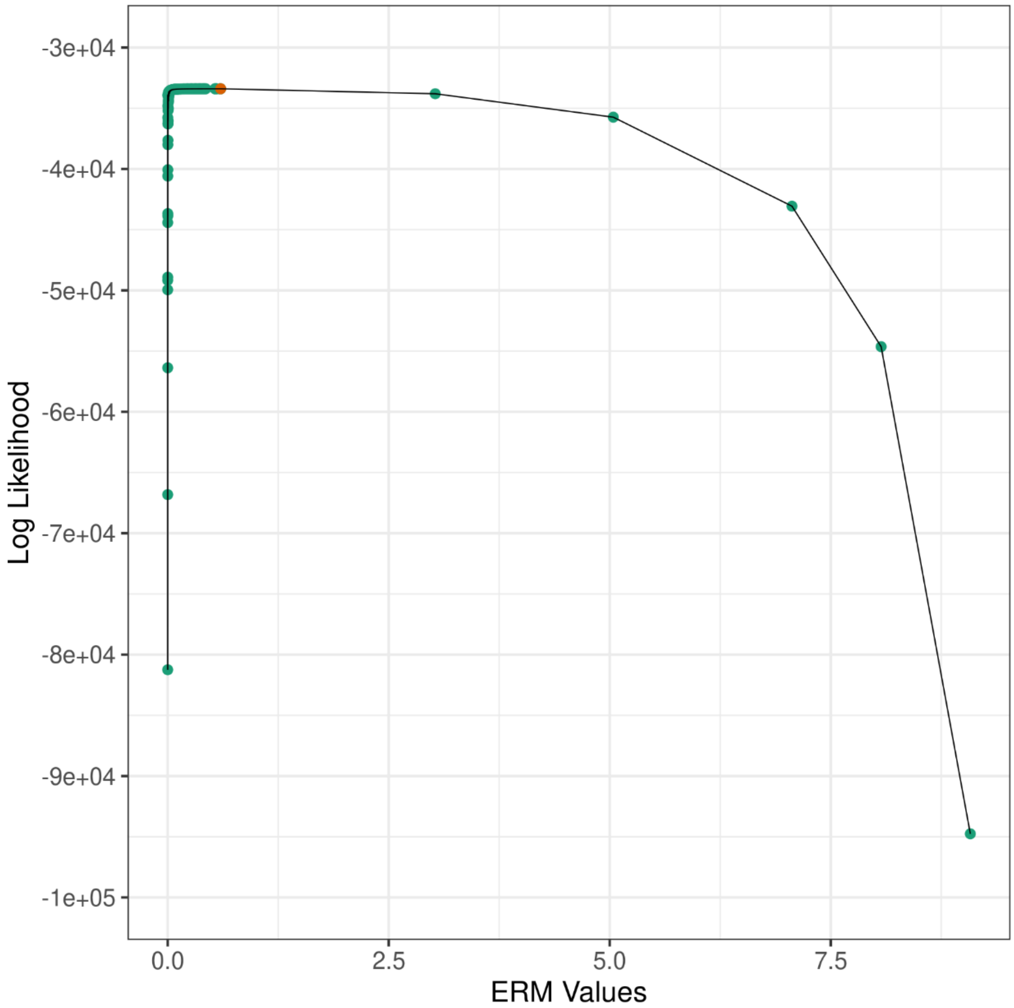


***Figure SB1***. Log-Likelihood Distribution of Mixed Linear Model Including the Trauma Exposure Environmental Relationship Matrix (ERM) for the UK Biobank North Geographical Cluster and Neuroticism Phenotype. The x-axis represents values of the ERM. The y-axis represents log-likelihood values. The orange point is the maximum likelihood estimate.

The distribution observed explains the discrepancy observed between the LRT values and the estimates/standard errors of the variance attributable to the E.

© 2022 Chuong M et al. *JAMA Psychiatry.*

## eReferences


1. Allen NE, Sudlow C, Peakman T, Collins R. UK biobank data: Come and get it. 2014.
2. Sudlow C, Gallacher J, Allen N, et al. UK biobank: an open access resource for identifying the causes of a wide range of complex diseases of middle and old age. *PLoS Med*. Mar 2015;12(3):e1001779. doi:10.1371/journal.pmed.1001779
3. Davis K, Hotopf M. Mental health phenotyping in UK Biobank. 2019.
4. Bycroft C, Freeman C, Petkova D, et al. The UK Biobank resource with deep phenotyping and genomic data. *Nature*. 2018;doi:10.1038/s41586-018-0579-z
5. Kessler RC, Ustun TB. The World Mental Health (WMH) Survey Initiative Version of the World Health Organization (WHO) Composite International Diagnostic Interview (CIDI). *Int J Methods Psychiatr Res*. 2004;13(2):93-121. doi:10.1002/mpr.168
6. American Psychiatric Association DSaAPA. Diagnostic and statistical manual of mental disorders: DSM-5. 2013;
7. Cai N, Revez JA, Adams MJ, et al. Minimal phenotyping yields genome-wide association signals of low specificity for major depression. *Nature Genetics*. 2020;doi:10.1038/s41588-020-0594-5
8. Eysenck SB, Eysenckm H. J., Barrett P. A revised version of the psychoticism scale. *Personality and individual differences*. 1985;6(1):pp.21-29.
9. Glaesmer H, Brähler E, Schulz A, Freyberger H, Grabe HJ, Häuser W. The childhood trauma screener (CTS) - Development and validation of cut-off-scores for classificatory diagnostics. *Psychiatrische Praxis*. 2013;doi:10.1055/s-0033-1343116
10. Grabe HJ, Schulz A, Schmidt CO, et al. [A brief instrument for the assessment of childhood abuse and neglect: the childhood trauma screener (CTS)]. *Psychiatr Prax*. Apr 2012;39(3):109-15. Ein Screeninginstrument fur Missbrauch und Vernachlassigung in der Kindheit: der Childhood Trauma Screener (CTS). doi:10.1055/s-0031-1298984
11. Davis KAS, Coleman JRI, Adams M, et al. Mental health in UK Biobank - development, implementation and results from an online questionnaire completed by 157 366 participants: a reanalysis. *BJPsych Open*. Feb 6 2020;6(2):e18. doi:10.1192/bjo.2019.100
12. Coleman JRI, Peyrot WJ, Purves KL, et al. Genome-wide gene-environment analyses of major depressive disorder and reported lifetime traumatic experiences in UK Biobank. *Molecular Psychiatry*. 2020;doi:10.1038/s41380-019-0546-6
13. Hoppen TH, Chalder T. Childhood adversity as a transdiagnostic risk factor for affective disorders in adulthood: A systematic review focusing on biopsychosocial moderating and mediating variables. *Clin Psychol Rev*. Nov 2018;65:81-151. doi:10.1016/j.cpr.2018.08.002
14. LeMoult J, Humphreys KL, Tracy A, Hoffmeister JA, Ip E, Gotlib IH. Meta-analysis: Exposure to Early Life Stress and Risk for Depression in Childhood and Adolescence. *J Am Acad Child Adolesc Psychiatry*. Jul 2020;59(7):842-855. doi:10.1016/j.jaac.2019.10.011
15. de Oliveira IR, Matos-Ragazzo AC, Zhang YN, et al. Disentangling the mental health impact of childhood abuse and neglect: A replication and extension study in a Brazilian sample of high-risk youth. *Child Abuse Neglect*. Jun 2018;80:312-323. doi:10.1016/j.chiabu.2018.03.021
16. Team RC. R: A language and environment for statistical computing. 2020.
17. Zhang F, Chen W, Zhu Z, et al. OSCA: a tool for omic-data-based complex trait analysis. *Genome Biol*. May 28 2019;20(1):107. doi:10.1186/s13059-019-1718-z


© 2022 Chuong M et al. *JAMA Psychiatry.*
